# *Ex vivo* HIV entry into blood CD4+ T cells does not predict heterosexual HIV acquisition in women

**DOI:** 10.1371/journal.pone.0200359

**Published:** 2018-07-09

**Authors:** Vineet Joag, Aida Sivro, Nonhlanhla Yende-Zuma, Hajra Imam, Natasha Samsunder, Quarraisha Abdool Karim, Salim Abdool Karim, Lyle McKinnon, Rupert Kaul

**Affiliations:** 1 Department of Immunology, University of Toronto, Toronto, Ontario, Canada; 2 Centre for the AIDS Program of Research in South Africa, University of KwaZulu-Natal, Durban, South Africa; 3 Department of Microbiology, University of Winnipeg, Manitoba, Canada; 4 Department of Epidemiology, Mailman School of Public Health, Columbia University, New York, New York, United States of America; 5 Department of Medicine, University of Toronto, Ontario, Canada; Fred Hutchinson Cancer Research Center, UNITED STATES

## Abstract

**Background:**

A blood-based assay that could quantify HIV susceptibility would be very valuable for HIV prevention research. Previously, we developed and validated an *ex vivo*, flow-based, HIV entry assay to assess genital HIV susceptibility in endocervical CD4+ T cells.

**Methods:**

Here we assessed whether this tool could be used to predict HIV risk using blood-derived CD4+ T cells in a rigorously-blinded, nested case-control study using blood samples collected from high-risk, HIV-uninfected South African women enrolled in the CAPRISA 004 clinical trial. Cases, subsequently acquiring HIV were sampled prior to HIV infection and compared with controls, who remained HIV-uninfected. The primary endpoint was *ex vivo* entry of a CCR5-tropic HIV founder virus into blood CD4+ T cells. Secondary endpoints included HIV entry into CD4+ central (T_CM_) and effector (T_EM_) memory T cells, and into CD4+ T cell subsets expressing CCR5, CD69, CCR6, α4β1 or α4β7.

**Results:**

Compared to bulk CD4+ T cells (4.9% virus entry), CD4+ T cells expressing CCR5, CCR6 or α4β1 and T_EM_ were highly susceptible (15.5%, 8.8%, 8.2% and 10.8% entry, respectively, all p<0.0001), while T_CM_, CD69+ or α4β7+ CD4+ cells were moderately susceptible (6.4%, 6.0% and 5.8% respectively, p ≤ 0.003). While the proportion of the aforementioned highly susceptible cells correlated with overall virus entry into CD4+ T cells within an individual (r = 0.68, 0.47, 0.67, and 0.60 respectively, p<0.0001), blood virus entry did not predict subsequent mucosal HIV acquisition after controlling for sexual behaviour and condom use (OR 0.92, 95% CI 0.77–1.11, p = 0.40).

**Conclusions:**

Although virus entry identified several previously known highly susceptible cellular HIV targets, blood HIV entry did not predict subsequent heterosexual HIV acquisition. Assessment of mucosal HIV susceptibility may require sampling at the site of HIV exposure.

## Introduction

There were approximately 1.4 million new HIV infections in Sub-Saharan Africa (SSA) in 2015, most of which were acquired in women through receptive vaginal sex [1]. Heterosexual vaginal HIV acquisition is generally regarded to be inefficient, with per-contact risk of HIV infection ranging from 1/200-1/2000 sex acts [2]. This inefficiency likely reflects the effectiveness of the mucosal host defenses including an intact epithelium, cervical mucus, immune cells (neutrophils, macrophages, T cells, dendritic cells, and others), and innate antimicrobial peptides (AMPs) such as alpha defensins and LL-37 [3].

The risk of HIV acquisition is enhanced by various factors including the HIV viral load of the transmitting partner, sexually transmitted infections (STIs), alterations in the vaginal flora and use of the injectable hormonal contraceptive Depo-Provera [4–6]. While host mucosal immune defenses may be protective, an over-exuberant immune response can be deleterious, as genital inflammation and/or an increased level of several AMPs were associated with an increased risk of HIV infection [7,8].

Genital inflammation can increase HIV risk through several mechanisms. Inflammation directly impairs the genital epithelial barrier [9,10] and also recruits HIV-susceptible CD4+ T cells to the genital mucosa [10–12]. An *ex vivo* HIV entry assay that directly quantifies virus entry into unstimulated cervical CD4+ T cells recently characterized genital and blood HIV target cells [12]. These included CD4+ T cells expressing CCR5 (HIV co-receptor), CD69 (early immune activation), α4β7 or α4β1 integrins (T cell homing) [12]. Other putative correlates include CCR6+ (Th17 cells) [13–15], T_EM_ (CD45RA-CCR7-), T_CM_ (CD45RA- CCR7+), and T_NAIVE_ (CD45RA+ CCR7+) CD4+ T cells.

Since the expression of some of these parameters in blood may correlate with those in the mucosa [12], we hypothesized that *ex vivo* HIV entry into blood CD4+ T cells would be an appropriate surrogate of subsequent heterosexual (mucosal) HIV acquisition, a finding that could have broad applicability for clinical monitoring and/or recruitment into future HIV prevention studies. To test our hypothesis, we conducted a nested case-control study to compare HIV entry into blood CD4+ T cells between HIV-uninfected women enrolled in the CAPRISA 004 clinical trial who subsequently acquired HIV infection (cases) and participants who remained HIV uninfected (controls) [16].

## Materials and methods

### Ethics statement

All clinical investigation was conducted in accordance with the principles expressed in the Declaration of Helsinki. The protocol for the CAPRISA 004 clinical trial (clinical trials NCT 00441298) and informed consent forms were approved by the University of KwaZulu-Natal, Ref: E111/06, the Protection of Human Subject Committee in the Office of International Research Ethics at FHI Ref: 9946, and the South African Medicines Control Council (MCC), Ref: 20060835.

### Study participants

The objective of this study was to conduct a retrospective case-control analysis to determine whether *ex vivo* HIV entry into blood CD4+ T cells obtained from HIV-uninfected women would be greater in participants that subsequently acquired HIV (cases) during the CAPRISA 004 clinical trial compared to controls that remained HIV-uninfected. We hypothesized that *ex vivo* HIV entry into blood CD4+ T cells obtained from HIV-uninfected women would be elevated in participants that subsequently acquired HIV. CAPRISA 004 was a randomized, placebo-controlled clinical trial that demonstrated 39% efficacy of a tenofovir 1% vaginal gel in preventing heterosexual HIV acquisition [16]. Participant eligibility criteria for the CAPRISA 004 clinical trial and methods of recruitment have been described in detail elsewhere (S1 File) [16]. Peripheral blood mononuclear cells (PBMCs) were cryopreserved at routine intervals after enrollment in CAPRISA 004, and HIV testing was performed monthly throughout the trial. Case and control samples were chosen based on availability of matched samples. Matching of cases and controls was performed for age (within a 5 year window), study arm (tenofovir gel versus placebo gel), the calendar date (month) of enrolment, time enrolled in the study, and also for the duration of cryopreservation (in months) prior to analysis of HIV entry and other flow-based analysis. These measures ensured that the sampling time point and length of follow-up had minimal effect, if any on CD4+ T cells and various subsets.

### BlaM-Vpr HIV pseudovirus production and infection assay

β-lactamase (BlaM)-Vpr HIV pseudovirions were prepared by transfection of HEK 293T cells with 20μg Q23ΔEnv, 10μg CCR5-tropic Clade C CAP45.2.00.G3 *env* [17], 10μg BlaM-Vpr and 5μg pAdvantage (Promega), as described elsewhere [12]. To maximize the dynamic range of the assay, input virus used in infections was equivalent to 50% of maximum infection assessed using reference PBMCs. To minimize day-to-day variation in sample processing, matched case-control samples were thawed on the same day. Twenty-two cases and 44 controls were matched at a 1:2 ratio, and due to limited sample availability, 11 cases and controls were matched at a 1:1 ratio.

*Ex vivo* HIV infections and flow cytometry acquisition and analysis (gating) were performed by study staff blinded to HIV acquisition status. After complete entry of immune and virus entry results, the dataset was sealed and only then was the case-control code broken to permit analysis of the immune associations of HIV acquisition.

### Flow cytometry

Surface staining of PBMCs was performed with fluorescently-labelled monoclonal antibodies that included CD3 Brilliant Violet (BV) 786, CD4 BV650, CCR5-PE-CF594, CCR7 BV605, CCR6 BV711, CD49d (α4) PE, β7 PE-Cy5, and CD29 (β1) PerCP-eFluor 710 (BD Biosciences), CD69 PE-Cy7 (EBioscience), CD45RA APC-Cy7 (Biolegend) and LIVE DEAD far red (Invitrogen). Flow cytometry sample acquisition was conducted on the LSR Fortessa (BD Biosciences).

### Statistical analysis

The sample size was calculated based on our previously reported data from a cohort of HIV-uninfected women from Nairobi, Kenya [12]. Based on those data, a standard deviation in % HIV entry into blood CD4+ T cells of 2.98% along with a Type I error of 0.05% and Type II error of 0.2 was used to estimate sample size. Since the difference in virus entry between cases and controls that is clinically meaningful was not known, we opted to detect a 40% difference in virus entry between the study groups, hence a sample size of 33 cases and 55 matched controls was chosen. The primary study endpoint was *ex vivo* entry of a CCR5-tropic HIV founder virus into blood CD4+ T cells. Secondary endpoints included HIV entry into CD4+ central (T_CM_) and effector (T_EM_) memory T cells, and into CD4+ T cell subsets expressing CCR5, CD69, CCR6, α4β1 or α4β7. The impact of HIV entry on subsequent HIV acquisition risk was assessed using univariate and multivariate conditional logistic regression models. Models incorporated previously defined HIV risk factors such as age, HSV-2 infection status, relationship status, history of vaginal discharge, DMPA use, sex acts in the past month, and condom use. If demographic data for aforementioned HIV risk factors was missing for any participants, these participants were excluded from the case/control analysis of *ex vivo* HIV entry into blood CD4+ T cells to allow for the adjusting for potential confounders. However, these data were included for assessment of the relative HIV susceptibility of various CD4+ T cell subsets.

Comparison of HIV entry into CD4+ T cells and various subsets was performed using the Wilcoxon matched-pairs signed ranks test. Within an individual, the association between the proportion of various CD4+ T cell subsets and overall HIV entry into CD4+ T cells was performed using spearman correlations. Flow cytometry data was analyzed using FlowJo X, exported into Microsoft Excel and further analyzed using PRISM6, SPSS (IBM) or SAS Version 9.4 on Mac OS.

## Results

### Study participants

The study included 33 cases and 55 controls (S1 Appendix). Participant demographic data was missing for 1 case and two matched controls, hence this set was excluded from the case/control analysis of *ex vivo* HIV entry into blood CD4+ T cells; however, these data were included for assessment of HIV target cells.

PBMCs from cases had been collected at a median of 110 days (IQR 65–182 days) prior to HIV acquisition. Cases reported a higher number of sex acts during the past month (median 8, IQR 4–12) than controls (median 6, IQR 3–9, OR 1.16, 95% CI (1.01–1.33), p = 0.03), but other demographic parameters were similar between groups, including age, HSV-2 infection status, relationship status, vaginal discharge, DMPA use and condom use (Table 1).

**Table 1:** Participant characteristics.

Characteristic	HIV sero-converters	HIV non sero-converters	OR, 95% CI	p value
**Median age in years, (IQR)**	21, IQR (20–24)	22, IQR (19–25)	0.98 (0.59–1.60)	0.92
Relationship status (stable)*	28 (88%)	51 (93%)	0.31 (0.06–1.72)	0.18
**HSV-2 infected**	19 (58%)	29 (53%)	1.32 (0.54–3.27)	0.99
Median number of sex acts/last month (IQR)*	8 (3–9)	6 (4–12)	1.16 (1.01–1.33)	0.03
**DMPA use**	22 (67%)	45 (81%)	0.68 (0.26–1.83)	0.45
**Vaginal discharge**	12 (36%)	17 (31%)	1.31 (0.47–3.65)	0.60
**Condom use (always)**	3 (9%)	17 (31%)	2.40 (0.85–6.73)	0.10
**Tenofovir use**	10/33 (30%)	16/55 (30%)	N/A	0.9

Univariate analysis depicting the number (percentage) of participants that were HIV-uninfected at enrollment but subsequently acquired HIV infection (HIV sero converters or cases, n = 33) and those that remained uninfected during the course of the CAPRISA 004 clinical trial (HIV non sero-converters or controls, n = 55). IQR = interquartile range, DMPA = depo-medroxy progesterone acetate, HSV = herpes simplex virus. N/A, not applicable.

* indicates parameters for which data was not available for 1 case and 2 controls.

### No association of HIV entry into blood CD4+ T cells with sexual HIV acquisition

**Fig 1:**
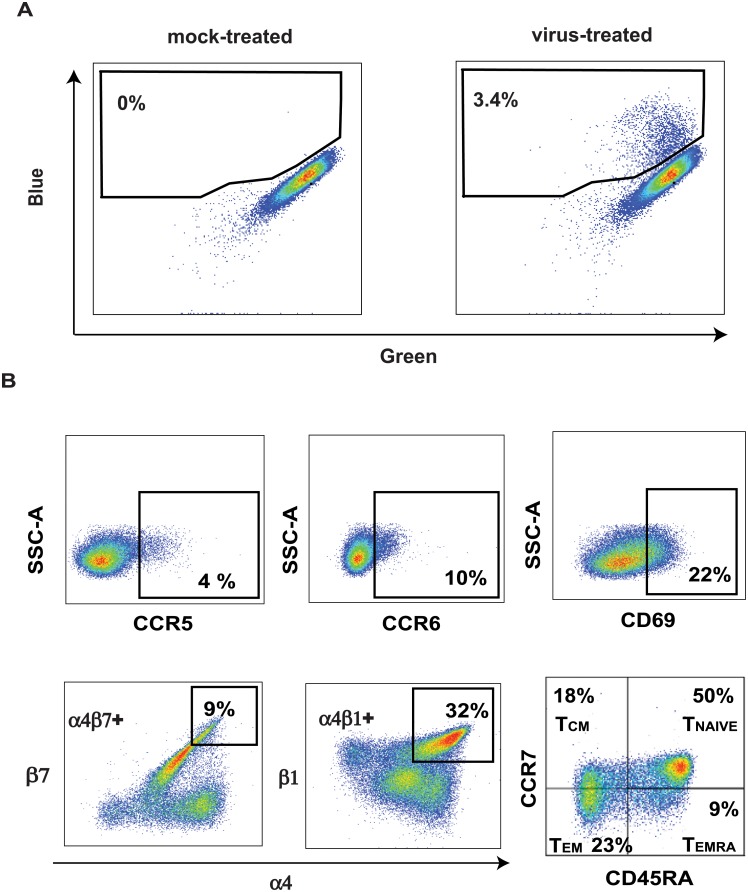
Gating strategy for analysis of HIV entry and various blood-derived CD4 T+ cell subsets. Representative plots of virus entry (**A**) gated on singlets, live cells, lymphocytes, and CD4+ T cells. Blue and green refer to cleaved and uncleaved CCF2-AM dye respectively. In (**B**), representative plots show gating strategy for various CD4+ T cell subsets as indicated.

**Fig 2:**
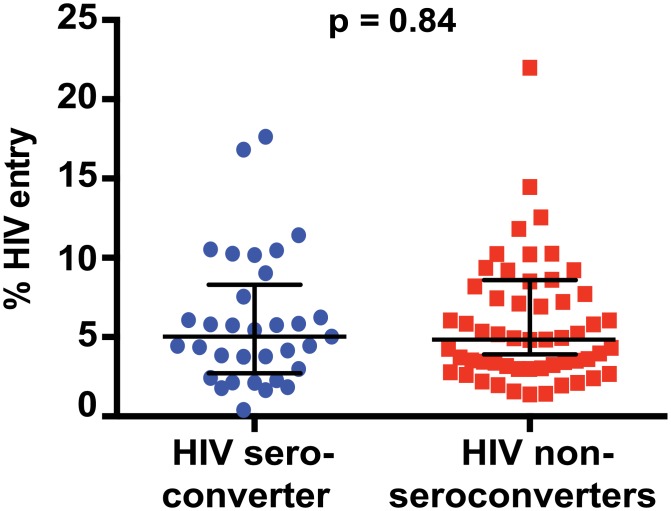
HIV entry assay identifies previously known HIV target cells, but virus entry into blood CD4+ T cells does not predict subsequent HIV acquisition. Comparison of percent HIV entry into blood CD4+ T cells of HIV-uninfected participants who subsequently acquired HIV (HIV sero-converters) or did not acquire HIV (non sero-converters).

*Ex vivo* HIV entry into blood CD4+ T cells did not differ between cases (median 5.0%; IQR 2.7%-8.3%) and controls (median 4.8%; IQR 3.0%-7.7%) in univariate analysis (OR 1.02, 95% CI 0.8–1.16, p = 0.82, Figs 1A and 2). This was also the case when the model incorporated previously defined risk factors such as age, HSV-2 infection status, relationship status, history of vaginal discharge, DMPA use, sex acts in the past month, and condom use (OR 0.92, 95% CI (0.77–1.11), p = 0.40). It is possible that a potential association between virus entry and HIV acquisition was diluted by the preventative effect of tenofovir in our case-control analysis. However, when we limited our analysis to participants that received placebo, we still did not observe an association between virus entry and HIV acquisition (OR 1.03, 95% CI, 0.83–1.28, p = 0.8.) We also did not observe an association between HIV acquisition status and the expression of various cell surface markers (Fig 1) or virus entry into CCR5+ or various memory CD4+ T cell subsets before or after controlling for aforementioned risk factors (p>0.05 for all). Overall, we found no evidence that *ex vivo* HIV entry into bulk blood CD4+ T cells was predictive of subsequent heterosexual HIV acquisition.

### Cellular correlates of *ex vivo* HIV entry

Next, we sought to validate that the virus entry assay was correctly identifying cell subsets expected to be highly susceptible to HIV. As expected from our use of a CCR5-tropic pseudovirus, CCR5+ CD4+ T cells were most susceptible to HIV entry compared to total CD4+ T cells (3.2 fold more susceptible, p<0.0001); T_EM_, CCR6+, and α4β1+ CD4+ T cell subsets also demonstrated enhanced cellular virus entry (2.2, 1.8, and 1.7 fold higher respectively, all p<0.0001, Fig 1B, Table 2). In addition, virus entry was significantly but more modestly elevated into T_CM_ cells, CD69+ CD4+ T cells, and α4β7+ CD4+ T cells (1.05–1.3 fold increase, p<0.05, Table 2). Naïve CD4+ T cells demonstrated substantially reduced susceptibility (5.4 fold lower virus entry, p<0.0001).

**Table 2:** HIV entry into various blood CD4+ T cell subsets.

CD4+ T cell subset	% HIV entry	p-value
Bulk	4.9% (3.0–7.7%)	
CCR5+	15.5% (12.0%-19.4%)	< 0.0001
TEM	10.8% (7.4%-14.4%)	< 0.0001
TCM	5.8% (3.3%-9.1%)	0.0004
Tnaive	0.9% (0.2%-2.4%)	< 0.0001
α4β1+	8.2% (5.2%-11.1%)	< 0.0001
α4β7+	6.0% (3.4%-9.4%)	0.003
CCR6+	8.8% (5.2%-12.2%)	< 0.0001
CD69+	6.4% (4.0%-10.8%)	< 0.0001

Wilcoxon matched-pairs signed rank test is used to compare virus entry into bulk CD4+ T cells and various T cell subsets.

**Fig 3:**
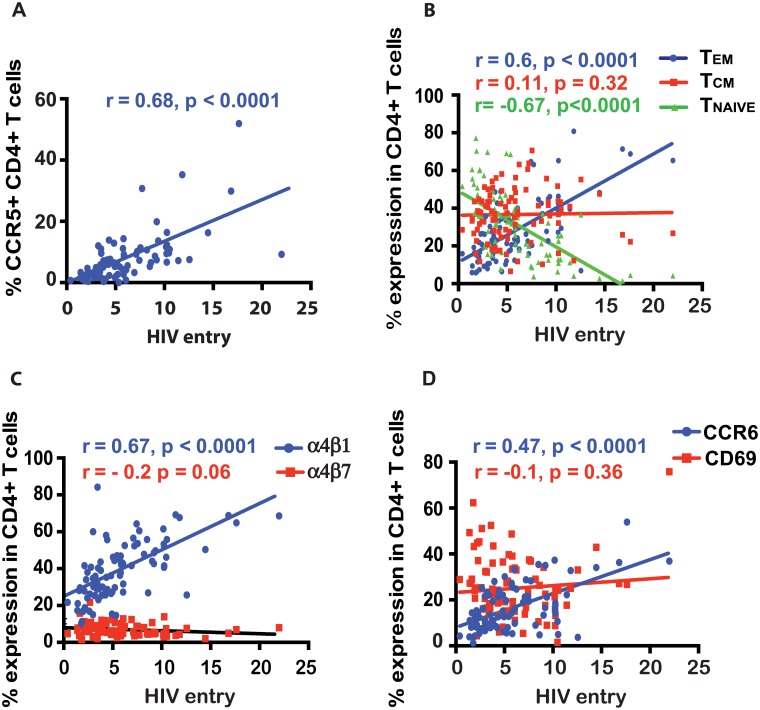
Correlation between bulk HIV entry into CD4+ T cells and the frequency of various blood CD4+ T cell subsets within an individual. Various blood CD4+ T cells are indicated in the figure legend (**A-D**). *, **, and *** indicate p values <0.01, <0.001 and <0.0001 respectively. TCM and TEM refer to central and effector memory CD4+ T cells respectively.

Within an individual, the proportion of CD4+ T cells expressing markers of high HIV susceptibility (CCR5+, α4β1+, CCR6+ CD4+ T cells or T_EM_ cells) was strongly correlated with overall HIV entry into bulk CD4+ T cells (r>0.47, p<0.0001 for all, Fig 3A–3D). Bulk virus entry did not correlate with the frequency of moderately susceptible cells (T_CM_, α4β7+ or CD69+ CD4+ T cells; all p>0.05, Fig 3B–3D), and was inversely correlated with the frequency of T_NAIVE_ cells (r = -0.67, p<0.0001, Fig 3B). Next we assessed whether the relationships between the frequency of highly susceptible cells and overall virus entry in CD4+ T cells in a blood sample were different between cases and controls. We found no major differences in the analysis stratified for HIV acquisition status, as the frequency of TEM and of CCR5+, CCR6+, or α4β1+ CD4+ T cells correlated strongly with HIV entry in cases and controls (r>0.38, p<0.0001 for all). On the contrary, the frequency of cells with moderate virus entry on a per cell basis (TCM, CD69+, or α4β7+ CD4+ T cells) was not associated with overall virus entry into bulk CD4+ T cells in either cases or controls (p>0.05 for all). Therefore, the HIV pseudovirus entry assay accurately identified several highly susceptible cell subsets, and the overall virus entry into blood CD4+ T cells within an individual was specifically driven by their frequency.

## Discussion

An improved understanding of the correlates of HIV acquisition would inform HIV prevention efforts. In particular, an assay that could rapidly assess HIV susceptibility using blood would permit the identification of participants at a high risk of HIV acquisition for subsequent provision of HIV pre-exposure prophylaxis (PrEP), or for enrollment in HIV clinical or vaccine trials. Therefore, we assessed the ability of an HIV entry assay previously validated in cervix-derived CD4+ T cells [12] to predict heterosexual HIV acquisition in high-risk South African women using blood samples collected and stored prior to HIV acquisition. Although expected CD4+ T cell subsets were identified as preferential HIV targets, we found no association between virus entry levels in blood-derived CD4+ T cells and the subsequent risk of sexual HIV acquisition.

The lack of association between blood virus entry and HIV risk can be interpreted in several ways. First and foremost, compartmentalization of biological parameters that alter HIV susceptibility in the female genital tract may render virus entry into blood CD4+ cells a poor proxy of heterosexual HIV risk. Some of these compartmentalized factors include the vaginal microbiota [4], genital infections [18–20] and the use of Depo Provera [5,21,22], which may either reduce epithelial barrier integrity, enhance cell-cell HIV transmission and/or alter post-HIV entry events [23]. If this is the case, then determination of HIV risk may require genital tract sampling to determine susceptibility in the compartment where HIV exposure will occur, even though the collection of such samples is more difficult in a field setting. Alternatively, assaying cellular virus entry/viral fusion with CD4+ T cells may not be an adequate proxy for HIV susceptibility, due to the multivariate nature of HIV transmission. Measurement of viral fusion alone does not consider downstream events in the HIV life cycle, the involvement of host barrier defenses such as the mucus or the epithelium, other infection-enhancing cell types such as dendritic cells or macrophages. For instance, the level of expression of CD69 in blood CD4+ T cells did not correlate with virus entry into bulk CD4+ T cells in our study, however, it did correlate with *ex vivo* HIV replication elsewhere [24]. The most likely explanation for this difference is that we assessed CCR5-dependent virus entry, while Card et al. measured viral replication. While CCR5 expression is the key determinant of cytoplasmic HIV entry of an R5-tropic HIV pseudovirus, virus replication is also dependent on a plethora of post-fusion factors including host restriction factors and the level of cellular immune activation (often measured by CD69 expression in blood). Cellular activation regulates processes critical to the HIV life cycle such as reverse transcription, viral integration and transcription. Hence, the difference in study endpoints may account for the difference in the findings from the two studies. However, the identification by our assay of T_EM_ or CCR5+, α4β1+, or CCR6+ CD4 T cells as most susceptible is consistent with previous findings by us and others [12,25]; this suggests that while our assay accurately reflected host susceptibility to HIV fusion of CD4+ T cells, virus entry in blood CD4+ T cells is not a proxy of mucosal HIV risk.

There are several potential limitations to our study. Our assay uses a higher viral inoculum than that occurs during *in vivo* vaginal virus exposure, where infection generally involves the expansion of a single founder virus [26,27]. While the higher inoculum allows robust detection of virus entry into CD4+ T cell subsets, we carefully titrated our pseudovirus and used sub-saturating levels in infectivity assays (input virus was ~ 50% of maximum infectivity of reference blood CD4+ T cells). Therefore, the overall virus infectivity within an individual was driven by host factors that influence viral fusion, enabling an assessment of host susceptibility to HIV. Another limitation is that the cryopreservation and subsequent thawing of clinical samples used in this study may have preferentially depleted certain cell subsets that may be important correlates of HIV risk. However, when we compared cellular frequencies from cryopreserved samples from this study with their frequencies in fresh samples from an independent study of HIV-uninfected women from Toronto, Canada (data not shown) we did not observe any notable differences. This suggested that at least ostensibly, depletion of particular cell subsets did not occur in our cryopreserved samples. Moreover, cases and controls in our clinical samples from the CAPRISA 004 cohort were matched based on calendar month of enrolment and cryopreserved for the same duration before analysis. This ensured that cryopreservation had similar effects (if any) on cases and controls. Lastly, this study had a relatively small sample size. While it is not known what difference in virus entry is clinically significant, we aimed to detect a 40% difference; since no trend to differential virus entry between cases and controls was apparent, it is unlikely that a larger sample size would have altered our conclusions.

## Conclusions

In summary, an *ex vivo* HIV entry assay identified highly-susceptible CD4+ T cell subsets in the blood of high-risk South African women. However, the level of virus entry into blood CD4+ T cells did not predict subsequent risk of heterosexual HIV acquisition, suggesting that sexual acquisition might be driven by compartmentalized mucosal parameters that can only be captured by mucosal sampling.

## Supporting information

S1 Appendix Study metadata. (XLSX)

S1 File CAPRISA 004 study protocol. Protocol for the CAPRISA 004 clinical trial.
(PDF)
