# Supplementary material for: Ex vivo HIV entry into blood CD4+ T cells does not predict heterosexual HIV acquisition in women
Source: PLoS One. 2018 Jul 9;13(7):e0200359. doi: 10.1371/journal.pone.0200359 (PMC6037376; doi:10.1371/journal.pone.0200359)
Supplement: S1 File — Protocol for the CAPRISA 004 clinical trial. (PDF) [file pone.0200359.s002.pdf]

CAPRISA 004

Phase IIb Trial to Assess the Safety and Effectiveness of the Vaginal Microbicide 1% Tenofovir Gel for the Prevention of HIV Infection in Women in South Africa

Trial design and conduct:
Centre for the AIDS Programme of Research in South Africa (CAPRISA), University of KwaZulu-Natal, Durban, South Africa,

Coordination of Product manufacture and packaging:
CONRAD, Virginia, USA

Statistical, regulatory and operational support:
Family Health International, North Carolina, USA

Co-Principal Investigators
Quarraisha Abdool Karim, PhD, and Salim S. Abdool Karim, MBChB, PhD
CAPRISA, University of KwaZulu-Natal

Version 1.3, 29 October 2010

Phase IIb trial to assess the safety and effectiveness of the vaginal 1% tenofovir gel for the prevention of HIV infection in women in South Africa

PROTOCOL TEAM ROSTER

Quarraisha Abdool Karim, PhD
Co-Principal Investigator
CAPRISA, Nelson R Mandela School of Medicine,
University of KwaZulu-Natal , Private Bag 7,
Congella 4013, Durban, South Africa
Abdoolq2@ukzn.ac.za
T: 27-31-260 4208
F: 27-31-260 4307

Salim S. Abdool Karim, MBChB, FFPHM, PhD
Co-Principal Investigator
CAPRISA, Nelson R Mandela School of Medicine,
University of KwaZulu-Natal, Private Bag 7,
Congella 4013, Durban, South Africa
karims1@ukzn.ac.za
T: 27-31-260 2381
F: 27-31-260 3093

Cheryl Baxter, MSc
Research Associate
CAPRISA , Nelson R Mandela School of Medicine,
University of KwaZulu-Natal , Private Bag 7,
Congella 4013, Durban, South Africa
Baxterc1@ukzn.ac.za
T: 27-31-260 4559
F: 27-31-260 4566

Janet Frohlich, DipMGC, DCurVulindlela Site
Operations ManagerCAPRISA Vulindlela Clinical
Research Site, Mafakatini Primary Health Care
Clinic, Mafakatini, Vulindlela, South Africa
frohlichj@ukzn.ac.za
T: 27-31-260 6851
F: 27-33-997 1425

Andy Gray, MSc (Pharm)
Senior Protocol Pharmacist
Department of Therapeutics and Medicines
Management and CAPRISA, University of
KwaZulu-Natal, Private Bag 7, Congella 4013,
Durban, SA
graya1@ukzn.ac.za
T: 27-31-260 4334
F: 27-31-260 4566

Anneke Grobler, MSc
Protocol Statistician
CAPRISA, Nelson R Mandela School of Medicine,
University of KwaZulu-Natal, Private Bag 7,
Congella 4013, Durban, South Africa
grobler@ukzn.ac.za
T: 27-31-260 4392
F: 27-31-260 4566

Ayesha BM Kharsany, PhD
Co investigator
University of KwaZulu-Natal, Private Bag 7,
Congella 4013, Durban, South Africa
kharsany@ukzn.ac.za
T: 27-31-260 4558
F: 27-31-260 4566

Leila Mansoor, PhD
Study Co ordinator
CAPRISA , Nelson R Mandela School of Medicine,
University of KwaZulu-Natal , Private Bag 7,
Congella 4013, Durban, South Africa
Mansoor@ukzn.ac.za
T: 27-31-260 4641
F: 27-31-260 4566

Koleka Mlisana, MBChB
Project Clinical Operations Director (eThekwini)
CAPRISA eThekwini Clinical Research Site,
University of KwaZulu-Natal, Private Bag 7,
Congella 4013, Durban, South Africa
mlisanak@ukzn.ac.za
T: 27-31-260 4562
F: 27-31-260 4566

Luvuyo Mtongana, MBChB
Study Clinician
CAPRISA, Nelson R Mandela School of Medicine,
University of KwaZulu-Natal, Private Bag 7,
Congella 4013, Durban, South Africa
mtongana@ukzn.ac.za
T: 27-31-260 4555
F: 27-31-260 4566

Sengeziwe Sibeko, MBChB, FCOG
Project Gynaecologist
CAPRISA, Nelson R Mandela School of Medicine,
University of KwaZulu-Natal, Private Bag 7,
Congella 4013, Durban, South Africa
sibekos@ukzn.ac.za
T: 27-31-260 4555
F: 27-31-260 4566

Douglas Taylor, PhD
Senior Protocol Statistician
Family Health International, PO Box 13950,
Research Triangle Park, NC 27709, USA
dtaylor@fhi.org
T: 1-919-544 7040 ext. 327
F: 1-919-544 0207

Phase IIb trial to assess the safety and effectiveness of the vaginal microbicide 1% tenofovir gel for the prevention of HIV infection in women in South Africa

ROSTER OF ORGANISATIONS:

CONRAD
Henry Gabelnick, PhD
Executive Director,
CONRAD, 1911 Fort Myer Drive., Ste. 900, Arlington, VA 22209,
hgabelnick@conrad.org
T: 1-703-276 3904
F: 1-703-524 4744

Family Health International
Willard Cates, Jr., MD, MPH
President: FHI
Family Health International, PO Box 13950, Research Triangle Park, NC 27709, USA
wcates@fhi.org
T: 1-919-544 7040
F: 1-919-544 0207

STUDY FUNDED BY:

United States Agency for International Development
***Attn:* Jeff Spieler, PhD**
Chief, Research, Technology and Utilization Division, Office of Population and Reproductive Health, Bureau for Global Health, USAID, 1300 Pennsylvania Ave, NW, Washington, DC 20523-3601
jspieler@usaid.gov
T: 1-202-712 1402
F: 1-202-216 3404

LIFELab
***Attn:* Blessed Okole, PhD**
Chief Executive Officer
LIFELab
PO Box 30603
Mayville, 4058
Durban, South Africa
Blessed.Okole@LifeLab.org.za T: 27-31-261 6427
F: 27-31-261 4581

PRODUCT SUPPLIES:

Packaged Tenofovir & Placebo gel supplies provided by:
CONRAD
***Attn:* Henry Gabelnick, PhD**
Executive Director, CONRAD
1611 North Kent St., Suite. 806
Arlington, VA 22209,
hgabelnick@conrad.org
T: 1-703-276 3904
F: 1-703-524 4744

Tenofovir Active Product Ingredient provided by:
Gilead Sciences
***Attn:* James F. Rooney, MD**
Vice President, Medical Affairs
Gilead Sciences
333 Lakeside Drive
Foster City, CA 94404
jrooney@gilead.com
T: 650-522-5708
F: 650-522-5854

STUDY MONITOR:
Family Health International
***Attn:* Amanda Troxler**
PO Box 13950
Research Triangle Park
NC 27709, USA
atroxler@fhi.org
T: 1-919-544-7040 ext. 11301
F: 1-919-544-7261

STUDY SPONSOR
Family Health International
***Attn:* David Borasky(Director, Regulatory Affairs and Quality Assurance)**
PO Box 13950
Research Triangle Park
NC 27709, USA
dborasky@fhi.org
T: 1-919-544 7040
F: 1-919-544 7261

ETHICS COMMITTEES:

University of KwaZulu-Natal Biomedical Research Ethics Committee
***Attn:* Douglas Wassenaar**
Faculty of Health Sciences Ethics Committee of the Nelson R Mandela School of Medicine, University of KwaZulu-Natal
Ethicsmed@ukzn.ac.za
T: 27-31-260 4495
F: 27-31-260 4410

Protection of Human Subject Committee
Family Health International
***Attn:* David Borasky**
Director, Office of International Research Ethics
2224 E Highway 54
Durham, NC 27713, USA
T: 1-919-544-7040 ext. 295
F: 1-919-544-0207

Phase IIb trial to assess the safety and effectiveness of the vaginal microbicide 1% tenofovir gel for the prevention of HIV infection in women in South Africa

CAPRISA 004

Signatures of Approval:

Co-Principal Investigator<br>Quarraisha Abdool Karim, PhD	29 October 2010<br>Date of Signature
Co-Principal Investigator<br>Salim S. Abdool Karim, MBChB, PhD	29 October 2010<br>Date of Signature
CONRAD<br>Henry Gabelnick, PhD	29 October 2010<br>Date of Signature
Family Health International<br>Willard Cates Jr, MD, MPH	29 October 2010<br>Date of Signature

Phase IIb trial to assess the safety and effectiveness of the vaginal microbicide 1% tenofovir gel for the prevention of HIV infection in women in South Africa

TABLE OF CONTENTS

PROTOCOL TEAM ROSTER 2
ROSTER OF ORGANISATIONS: 3
TABLE OF CONTENTS 5
ABBREVIATIONS AND ACRONYMS 8
SCHEMA 9
1 INTRODUCTION 10
1.1 Epidemiology of HIV 10
1.2 Microbicides and Developmental Pipeline 10
1.3 Study Product 11
1.3.1 Prior Research 11
1.4 Dosing strategy of tenofovir gel 15
2 STUDY SETTING 16
2.1 CAPRISA Clinical Research Site: Vulindlela 16
2.2 CAPRISA Clinical Research Site: eThekwini 17
3. STUDY OBJECTIVES AND DESIGN 18
3.1 Primary Objective 18
3.2 Secondary Objectives 18
3.3 Ancillary Objectives 18
3.4 Study Overview 18
3.4.1 Design 18
3.4.2 Accrual and Follow-up 18
3.4.3 Enrolment and Randomisation 19
3.4.4 Summary of Assessments 19
3.4.5 Safety Assessments 19
3.4.6 Outcome Assessment 19
3.4.7 Clinical management of non-study conditions 20
4 STUDY POPULATION 20
4.1 Inclusion Criteria 20
4.2 Exclusion Criteria 21
4.3 Recruitment, Screening, and Enrolment 21
4.3.1 Sources for study participants 21
4.3.2 Cohort recruitment and accrual 22
4.3.3 Screening and enrolment 22
4.4 Co-Enrolment Guidelines 23
4.5 Participant Retention 23
4.6 Participant Withdrawal 23
5 STUDY TREATMENT CONSIDERATIONS 23
5.1 Product Formulation 23
5.1.1 Tenofovir gel 23
5.1.2 Placebo Gel 23
5.2 Product Use Regimen 24
5.3 Product Management 24
5.3.1 Supply 24
5.3.2 Storage 24
5.3.3 Dispensing 25
5.3.4 Accountability 25
5.4 Adherence Counselling 25
5.5 Adherence Assessment 25
5.6 Discontinuation of Product 25
5.7 Concomitant Medications 26

6 STUDY PROCEDURES (SEE APPENDIX I: SCHEDULE OF EVALUATIONS) 26
6.1 Targeted |Recruitment 26
6.2 Screening Part 1 Visit (up to day -30) 26
6.2.1 Administrative, Behavioural, and Regulatory Procedures 26
6.2.2 Clinical Procedures 26
6.2.3 Laboratory Procedures 26
6.3 Screening Part 2 27
6.3.1 Administrative, Behavioural, and Regulatory Procedures 27
6.3.2 Clinical Procedures 27
6.4 Enrolment Visit (day 0) 27
6.4.1 Administrative, Behavioural, and Regulatory Procedures 27
6.4.2 Clinical Procedures 27
6.4.3 Laboratory Procedures 27
6.4.4 Pharmacy Procedures 27
6.5 Follow-up Visits 27
6.5.1 Administrative, Behavioural, and Regulatory Procedures 28
6.5.2 Pharmacy Procedures 28
6.5.3 Clinical Procedures 28
6.5.4 Laboratory Procedures 29
6.6 Interim Contacts and Visits 29
6.7 Study Exit Visit 29
6.7.1 Administrative and Behavioural Procedures 29
6.7.2 Clinical Procedures 29
6.7.3 Pharmacy Procedures 29
6.7.4 Laboratory Procedures 30
6.8 Post trial HIV test Visit 30
6.8.1 Clinical Procedures 30
6.8.2 Laboratory Procedures 30
6.9 Final Contact 30
7 SAFETY MONITORING AND ADVERSE EVENT REPORTING 30
7.1 Adverse Events and Reporting Requirements 30
7.2 Adverse Event Reporting 30
7.2 Serious Adverse Event (SAE) Reporting 31
7.3 Safety Monitoring 31
7.4 Data, Safety, Monitoring Board 32
8 STATISTICAL CONSIDERATIONS 32
8.1 Review of Study Design 32
8.2 Endpoints 32
8.2.1 Primary Endpoints 32
8.2.2 Secondary endpoints 32
8.2.3 Ancillary endpoint 33
8.3 Accrual, follow-up, and sample size 33
8.4 Random Assignment and allocation concealment 34
8.5 Blinding 35
8.6 Data Analysis 35
8.7 Data Management 36
9 HUMAN SUBJECTS CONSIDERATIONS 36
9.1 Regulatory and Ethical Review 36
9.2 Informed Consent 36
9.3 Risks 37
9.4 Benefits 38
9.5 Access to HIV-related care 38
9.5.1 HIV counselling and testing 38
9.5.2 Care for participants identified as HIV-infected 38
9.6 Community involvement and consultation: 38
9.7 Confidentiality 39
9.8 Study Discontinuation 39

10 LABORATORY CONSIDERATIONS 39
10.1 Laboratory Specimens 39
10.2 On site testing 39
10.3 Collection and shipping of specimens 39
10.4 Specimen Storage for Quality Assurance and Potential Future Research Testing 40
10.5 Laboratory Quality Control and Quality Assurance Procedures 40
11 ADMINISTRATIVE PROCEDURES 40
11.1 Protocol Compliance 40
11.2 Protocol violations 40
11.3 Quality Assurance and Study Monitoring 40
11.3.1 Study Monitoring 41
11.3.2 Auditing 41
11.4 Study Records 41
11.5 Use of Information and Publications 41
12 REFERENCES 42

APPENDICES 44
Appendix II: Safety Laboratory Evaluations 45
Appendix III: HIV Antibody Testing Algorithm 46
Appendix Iva: Informed Consent Form for Screening Participants (separate document) 47
Appendix IVb: Informed Consent Form for Enrolling Participants (separate document) 48
Appendix IVc: Informed Consent Form for Specimen Storage and Possible Future Research Testing (Separate document) 49

ABBREVIATIONS AND ACRONYMS

AE	adverse event
AIDS	Acquired Immunodeficiency Syndrome
ANC	Antenatal clinic
ALT	Alanine aminotransferase
AUC	Area Under the Curve
BID	Twice a day
CAPRISA	Centre for the AIDS Programme of Research in South Africa
CDC	Communicable Disease Centre
CI	confidence interval
C_{max}	maximum concentration
CP	Community Programme
CRSG	Community Research Support Group
CRF	Case Report Form
DSMB	Data and Safety Monitoring Board
ELISA	enzyme-linked immunosorbent assay
FDA	(United States) Food and Drug Administration
FHI	Family Health International
FIV	Feline Immunodeficiency Virus
FPC	Family Planning clinic
GCP	Good Clinical Practice
GMP	Good Manufacturing Practice
HEC	hydroxyethylcellulose
HIV	Human Immunodeficiency Virus
HBV	Hepatitis B Virus
HPTN	HIV Prevention Trials Network
HPV	human papillomavirus
HSV-2	herpes simplex virus-2
ICH	International Conference on Harmonization
ITT	Intention to treat
IV	intravenous
LLOQ	lower limit of quantitation
MCC	Medicines Control Council
MOP	Manual of Procedures
N-9	nonoxynol-9
PBMC	Peripheral blood mononuclear cells
PCR	Polymerase chain reaction
PHC	Primary Health Care
PID	Participant identification
PK	Pharmacokinetics
PSRT	Protocol Safety Review Team
RNA	Ribonucleic Acid
SAE	serious adverse event
SAP	Statistical Analysis Plan
SC	subcutaneous
SIV	Simian Immunodeficiency Virus
SOP	Standard Operating Procedures
STI	sexually transmitted infection
TDF	Tenofovir disoproxil fumarate
T_{max}	Time to maximum concentration
UNAIDS	Joint United Nations Programme on HIV/AIDS
USA	United States of America
VCT	Voluntary counselling and testing
w/w	weight per weight
w/v	weight per volume

Phase IIb trial to assess the safety and effectiveness of the vaginal microbicide 1% tenofovir gel for the prevention of HIV infection in women in South Africa

SCHEMA

Purpose: To assess the safety and effectiveness of tenofovir gel, a candidate vaginal microbicide, in sexually active women at risk for human immunodeficiency virus (HIV) infection in South Africa.

Design: Phase IIb, two-arm, double-blind, randomised, controlled trial comparing 1% tenofovir gel with a placebo gel.

Study Population: Sexually active, HIV-uninfected women aged 18 to 40 years in South Africa

Study Size: Up to 1250 women

Treatment Regimen: Participants will be provided with a supply of single-use, pre-filled applicators according to their randomisation. While in the study, participants will be asked to apply a first dose of the assigned study product, 1% tenofovir gel or placebo gel, within 12 hours prior to coitus and insert a second dose as soon as possible within 12 hours after coitus. They will be advised to use only two doses of gel in a 24-hour period.

Study Duration: Approximately 30 months in total. Accrual will require approximately 18 months and follow-up will continue until 92 incident HIV infections are observed in the study, which is expected to occur approximately 12 months after the end of the accrual period.

Primary Objective:
To evaluate the effectiveness and safety of a candidate vaginal microbicide, tenofovir gel, when applied intravaginally by women, in preventing sexually transmitted HIV infection.

Secondary Objectives:
- To assess the impact, if any, of tenofovir gel on the incidence rate of deep epithelial disruption
- To assess the impact, if any, of tenofovir gel on viral load in women who become infected with HIV during the trial.
- To assess tenofovir resistance in HIV seroconvertors in the trial
- To ascertain the impact, if any, of tenofovir gel on pregnancy rates and outcomes
- To assess the impact, if any, of product hold at study exit on HIV infection and tenofovir resistance

Ancillary Objective
- To assess the impact, if any, of tenofovir gel in preventing sexually transmitted infections, including. herpes simplex virus type 2 (HSV-2) and human papillomavirus (HPV) infections.

Study sites:
- CAPRISA Vulindlela Clinical Research Site, KwaZulu-Natal, South Africa
- CAPRISA eThekwini Clinical Research Site, Durban, South Africa

1 INTRODUCTION

1.1 Epidemiology of HIV

South Africa is experiencing one of the largest and fastest growing HIV epidemics in Sub-Saharan Africa and the world[1] About 6 million people are living with HIV/AIDS in South Africa[2]. There are many factors that contribute to the unprecedented, explosive spread of HIV in South Africa. The economically-motivated population migration with its associated disruption of conjugal stability and family units is a key factor driving the epidemic in Southern Africa[3]. Sixty percent of all infected adults acquire their infection before age 25, and young women between the ages of 20-24 years have the highest HIV prevalence and incidence rates[4]. The national HIV prevalence in prenatal women continues to rise, and in 2004, the most recent national estimate available, show that these rates have reached a new high of 29.5% (95% Confidence Interval (CI) 28.5 – 30.5). These national rates mask variations within South Africa as the prevalence of HIV varies by province, and is highest in KwaZulu Natal with a prevalence of 40.7% (95% CI 38.8 – 42.7) in 2004. This rate is matched by the HIV rates we have observed [42.7% (95% CI 38.5 - 47.0)] by replicating this survey among pregnant women at our rural clinical research site in Vulindlela, KwaZulu Natal. These trends have been increasing annually at all three levels (national; provincial; local). The majority of new infections are heterosexually transmitted with the highest incidence rates in women. Some indication of the importance of the heterosexual component of the global burden of HIV infection can be gleaned from the Joint United Nations Program on HIV/AIDS (UNAIDS) estimates at the end of 2003[5]. Of the estimated 37.8 million prevalent HIV infections, about 87% were acquired through heterosexual transmission. Further, of the 4.8 million new HIV infections and 2.9 million Acquired Immune Deficiency Syndrome (AIDS) deaths in 2003, over 85% were in persons who most likely acquired HIV infection heterosexually. These data underscore the importance of this mode of transmission.

Not only is heterosexual transmission important in driving the current major epidemic in sub-Saharan Africa but is also a major factor driving the emerging epidemics in India and China. The geographical distribution of the modes of HIV transmission, indicates that heterosexual transmission of HIV is an important mechanism of transmission beyond sub-Saharan Africa to most countries around the world and particularly in south and south-east Asia where a quarter of the new infections in 2003 occurred.

This mode of spread, which predominates in the global epidemic, is influenced by several key epidemiological factors such as age, gender, mobility, sexual partner profile, and the presence of other sexually transmitted infections (STIs). A striking characteristic of heterosexual transmission is the disproportionate burden of HIV infection in women compared to men[6]. In addition to an estimated 7-fold greater efficiency of transmission from men to women compared to women to men, social, cultural and economic factors also contribute to the excess infection observed in women compared to men[7]. Further, women acquire HIV infection at a younger age, at least 5-10 years earlier than men. Young boys aged 15-19 years have lower rates of HIV while teenage girls are already close to peak prevalence[8, 9]. In most African countries where heterosexual transmission is the major mode of transmission, a key factor in the epidemic is the high incidence rates in young women between the ages of 14 and 24 years as a result of sexual coupling with older men[10].

Notwithstanding the greater vulnerability of women, current options to reduce transmission and acquisition of HIV infection remain limited for women. There is a clear need for new technologies to prevent the sexual transmission of HIV in women. Correct and consistent use of male condoms has been shown to prevent HIV transmission[11], but women often are unable to negotiate use of condoms by their male partners[12-14]. The female condom has been marketed as an alternative barrier method, but this device is relatively costly. It requires a certain level of skill to use and acceptance by the male partner. There is clearly a need for new technologies to prevent the sexual transmission of HIV in women.

1.2 Microbicides and Developmental Pipeline

Topical microbicides are products designed to prevent the sexual transmission of HIV and other disease pathogens[12-15]. Potentially, they can be applied vaginally to prevent both male-to-female and female-to-male transmission. They also offer a female-controlled option in cases where neither male or female condom use can be negotiated. Marketed chemical spermicides, which have shown some activity against HIV and STI pathogens in vitro, have been evaluated as topical microbicides. The most notable of these was nonoxynol-

9 (N-9), which has been tested in various doses and formulations, but was shown to be ineffective in preventing HIV and possibly harmful[16-18].

Table 1 is a summary of the current stage of testing for microbicides in the developmental pipeline. Microbicides currently under development operate in one of four ways.

1) Surfactants operate by disrupting cell membranes and have a wide spectrum of activity against several microbes, spermatozoa and cell membranes. Since the testing of N-9, a newer agent, C31G (Savvy), which has a better safety profile, has been developed.
2) Vaginal defence enhancers boost the body's natural defences against infection by maintaining the naturally acidic environment of the vagina by increasing lactobacilli or by rapidly acidifying alkaline ejaculate. This process inactivates both sperm and STIs. BufferGel™ operates in this manner.
3) Entry and fusion inhibitors bind to pathogens or to healthy cells before pathogens can attach or invade them. Carraguard®, PRO 2000, and Cellulose sulfate (UsherCell™) operate in this manner. Cellulose sulfate inhibits sperm function and has recently demonstrated its contraceptive effectiveness in a 200 patient Phase II clinical study in the U.S..
4) Replication inhibitors, inhibit normal reverse transcription of viral RNA into mammalian DNA in host (infected) cells. Reverse transcriptase inhibitors are highly specific for a virus species, One such product is tenofovir gel, developed by Gilead Sciences. Tenofovir gel is designed to protect against HIV infection by inhibiting viral replication in susceptible cells.

Table 1: Developmental Pipeline of Microbicides under Evaluation

Phase				
**I**	**I / II**	**II**	**IIb**	**III**
• Ethanol in Emollient Gel<br>• Dapivirine<br>• TMC120<br>• UC-781<br>• VivaGel/SPL7013™<br>• CAP vaginal soft tablet (planned)<br>• PC 815 (planned)	• Invisible Condom ™<br>• VivaGel®†	• 1% Tenofovir gel<br>• Invisible Condom™ (Planned)	• BufferGel™ & PRO2000 (0.5%)<br>• Tenofovir gel (1%) in CAPRISA 004	• PRO 2000 (0.5%;2%)<br>• BufferGel®‡ (planned)

Table adapted from Alliance for Microbicide Development website at www.microbicide.org accessed December 17, 2008 which included the following disclaimer. "This Update is a brief overview and summary of the microbicide pipeline, produced and disseminated at the beginning of every month. As changes occur frequently, please visit www.microbicide.org for the most recent information or details, and/or contact Alliance Research Associate Stephanie Tillman (stillman@microbicide.org) for further information.".

1.3 Study Product

The study product is a gel formulation of tenofovir (PMPA, 9- [(R)-2-phosphonomethoxy)propyl]adenine monohydrate), an antiviral manufactured by Gilead Sciences, Inc., and licensed to CONRAD. Tenofovir is an adenosine nucleoside monophosphate (nucleotide) analog with potent activity against retroviruses. An orally bioavailable form of tenofovir (tenofovir disoproxil fumarate, tenofovir DF, Viread), has been approved for the treatment of HIV-1 infection since 2001 in the US and 2002 in Europe. It is estimated that over 200,000 HIV infected patients have received treatment with tenofovir DF. An intravenous (IV) formulation of this antiviral compound and an oral prodrug (Tenofovir Disoproxil Fumarate - TDF), has also been developed, approved and actively marketed by Gilead Sciences for the treatment of human immunodeficiency virus type-1 (HIV-1) infection in combination with other antiretroviral agents. Given the established safety and efficacy profile of TDF and the protective efficacy with tenofovir gel in monkey models, evaluation of tenofovir gel for prevention of HIV-1 in clinical studies is warranted.

1.3.1 Prior Research

The novel selective broad-spectrum anti-DNA virus agent, (S)-9-(3-hydroxy-2-phosphonylmethoxypropyl) adenine (HPMPA) [19-22], served as the prototype compound for the development of a series of widely used

therapeutic acyclic nucleoside phosphonates. These include cidofovir (HPMPC) in the treatment of papilloma-, herpes-, adeno- and poxvirus infections, adefovir (PMEA) in the treatment of chronic hepatitis B virus (HBV) infections, and tenofovir (PMPA) in the treatment of acquired immunodeficiency syndrome (AIDS)[23].

Tenofovir demonstrated potent activity against a wide range of retroviruses, i.e. simian immunodeficiency virus (SIV), feline immunodeficiency virus (FIV), visna-maedi virus, and murine leukaemia/sarcoma viruses and hepadnaviruses, Given that the precursors of tenofovir are active against other STIs, it is biologically plausible that tenofovir could have activity against other STIs, but further research is required.

Antiviral effects

The *in vitro* antiviral activity of tenofovir against laboratory and clinical isolates of HIV-1 was assessed in lymphoblastoid cell lines, primary monocyte/macrophage cells and peripheral blood lymphocytes. Tenofovir displayed antiviral activity *in vitro* against HIV-1 clades A, B, C, D, E, F, G and O (IC50 values ranged from 0.5 µM to 2.2 µM). In drug combination studies of tenofovir with nucleoside reverse transcriptase inhibitors (abacavir, didanosine, lamivudine, stavudine, zalcitabine, zidovudine), non-nucleoside reverse transcriptase inhibitors (delavirdine, efavirenz, nevirapine), and protease inhibitors (amprenavir, indinavir, nelfinavir, ritonavir, saquinavir), additive to synergistic effects were observed. Most of these drug combinations have not been studied in humans.

Prevention of Simian Immunodeficiency Virus transmission in animal models

Multiple studies have demonstrated the efficacy of tenofovir in preventing transmission of SIV in simian models[24]. Tenofovir has been successfully administered as pre-exposure or post-exposure prophylaxis[25, 26]. These studies have established tenofovir as a promising antiretroviral agent for the prevention of SIV infection. In one study, subcutaneous (SC) injection of tenofovir daily for 4 weeks in macaques resulted in 100% protection against acute SIV infection in a total of 25 treated animals without toxicity whether administration began 48 hours prior to IV inoculation (dose of tenofovir, 20 mg/kg in five animals, 30 mg/kg in 10 animals), 4 hours after inoculation (dose of tenofovir 30 mg/kg in five animals), or 24 hours post-inoculation (dose of tenofovir, 30 mg/kg in five animals). Evidence of SIV infection was not present in any of the treated animals monitored for up to 52 weeks, including viral load in plasma and peripheral blood mononucleocytes (PBMCs), SIV DNA in PBMCs, SIV-specific antibody, and lymph node biopsy. In contrast, each of 10 animals receiving placebo 48 hours prior to inoculation became infected [26].

The ability of tenofovir to prevent the establishment of persistent infection was investigated in a study on cynomolgus macaques (*Macaca fascicularis*). Daily SC dosing with tenofovir was initiated at varying times, as well as different durations of treatment, following intravenous inoculation with SIV. Twenty-four macaques were studied for 46 weeks after inoculation with SIV. All mock-treated control macaques showed evidence of infection within 2 weeks post-inoculation. All macaques that were treated with tenofovir for 28 days beginning 24 hours post-inoculation showed no evidence of viral replication following discontinuation of tenofovir treatment. However, extending the time to initiation of treatment from 24 to 48 or 72 hours post-inoculation or decreasing the duration of treatment reduced effectiveness in preventing establishment of persistent infection. Only half of the macaques treated for 10 days, and none of those treated for 3 days, were completely protected when treatment was initiated at 24 hours. Despite the reduced efficacy of delayed and shortened treatment, all tenofovir-treated macaques that were not protected showed delays in the onset of cell-associated and plasma viremia and antibody responses compared with mock controls[27].

In a study investigating the efficacy of tenofovir gel to prevent SIV infection, intravaginal application of 10% tenofovir gel weight per weight (w/w) administered 24 hours before, 0 hours, 24 hours after and 48 hours after intravaginal inoculation of SIV infection at 0 and 24 hours resulted in 100% protection in four female rhesus macaques, compared with evidence of infection in each of the two animals receiving placebo (vehicle only) [28]. In another study, 1% tenofovir gel (w/w) administered at three time points; 24 hours before, 15 minutes before, and 24 hours after a single intravaginal inoculation of SIV, resulted in 80% protection in five female rhesus macaques, comparable to that of the 10% tenofovir gel (w/w) group. Sixty percent protection was achieved in a third group of macaques which only received a single application of 1% tenofovir gel (w/w) 15 minutes before SIV challenge. Treated macaques were monitored for a total of 20 weeks, by virus

isolation from PBMCs. Further evidence of the efficacy of tenofovir in preventing infection following SIV exposure is provided by a study of pig-tailed macaques. Tenofovir was initiated 12, 36 and 72 hours following HIV exposure. Systemic infection was not evident in the 12 and 36 hour post exposure group (n=8) as defined by plasma viremia, cell-associated provirus, antibody response, and lymph node virus. However, breakthrough infection in one animal in the 72 hour post HIV exposure group was detected at 16 weeks [(29)].

A study investigating the protection of macaques against rectal SIV challenge showed that rectal pre-dosing with tenofovir gel has potential as a microbicide strategy. Mucosally-applied tenofovir gel was given rectally as a single dose 15 minutes or 2 hours prior to, or 2 hours after, intrarectal challenge. In the 2 control groups of macaques, 4 of 4 untreated macaques and 3 of 4 macaques given placebo gel became infected. Virus was recovered from only 1 of 6 animals receiving tenofovir gel 15 minutes prior to virus challenge. In 1 other animal in this group virus was recovered only at weeks 2 and 6. Virus was not recovered from any of the other 4 animals. Two of 3 animals receiving the drug 2 hours prior to virus challenge showed no evidence of circulating virus and in the third animal virus isolation was delayed until week 12. In the third intervention group where gel was administered 2 hours after virus challenge, 2 out of the 3 animals became infected. Interestingly, gag-specific interferon-gamma secreting T cells were detected by ELISpot in 4 of 7 animals in which virus was unrecoverable from PBMC. These T-cell responses confirm exposure to challenge virus antigens and suggest that infection did not become established despite the virus having triggered an immune response[(30)].

Such *in vivo* activity of an antiviral compound makes tenofovir a promising agent for prevention of HIV infection and available evidence shows that tenofovir gel can provide potent protection against SIV infection even, in some instances, when applied several hours after SIV challenge.

Safety of tenofovir gel

In animal efficacy and toxicity studies, tenofovir was found to be well tolerated even at high doses administered parenterally (subcutaneously or via IV infusion) or orally over prolonged periods. In rat and rabbit models, tenofovir gel (0.3-10.0%) caused minimal to mild local irritation following intravaginal administration. In rats, daily intravaginal administration of tenofovir gel (1-10% for 14 days) produced no evidence for local irritation or systemic toxicity as evidenced by no gross alterations in tissues and organs within the thoracic and abdominal cavities, and no histological lesions in the reproductive tissues (cervix, ovaries, uterine horns, vagina, vulva) or kidneys. In a 10-day rabbit vaginal irritation study, irritation was minimal in animals treated with 0.3-1.0% tenofovir gel. At higher doses (3-10%), tenofovir gel caused mild local irritation (increased leucocytic infiltrates, congestion and, in some cases, slightly increased edema) and produced average irritation scores similar to the control (Conceptrol). However, unlike Conceptrol, no animals treated with tenofovir had epithelial erosion or ulceration.

Clinical data on the safety of tenofovir gel comes from HPTN 050, a phase I safety and tolerability study among 84 low risk women who applied either 0.3% or 1% tenofovir gel once or twice daily for 14 days. The 1% tenofovir gel formulation was well tolerated in both HIV negative and HIV positive women. The majority of adverse events (AEs) reported in HPTN 050 were mild (87%) and limited to the genitourinary tract (77%). Four severe AEs were reported, but only one, lower abdominal, pain was thought to be product-related. No clinically significant systemic toxicity was observed and the tenofovir gel in this study showed a beneficial effect on vaginal microflora [(31)]. A recently completed study of 200 women from the USA and India (HPTN 059) reported that both daily and coitally dependent use of tenofovir gel was acceptable and safe[(32)].

Oral TDF is a Food and Drug Administration (FDA) category B drug. Reproduction studies have been performed in rats and rabbits at doses up to 14 and 19 times the human dose based on body surface area comparisons and revealed no evidence of impaired fertility or harm to the foetus due to tenofovir. However, there are no specific studies of the oral formulation of tenofovir in pregnant women, Therefore, while pregnancy is not a specific contraindication to use, tenofovir is not recommended for unqualified use in pregnant women. Use in pregnancy is cautioned due to the lack of human safety data in pregnancy. An Antiretroviral Pregnancy Registry has been established to monitor foetal outcomes of pregnant women exposed to oral TDF. However, this registry has very limited data relating to tenofovir which may indicate

that it is not being used sufficiently widely in pregnancy yet or it may indicate that the drug's good safety profile has resulted in few entries.

Pharmacokinetics and systemic absorption

The pharmacokinetics (PK) of intravaginal tenofovir were examined in female rabbits following administration of a single dose of 0.5 mL of tenofovir gel containing 1% weight in volume (w/v) tenofovir (5 mg tenofovir per animal; 50 µCi/kg). Concentrations of radioactivity in plasma were highest (0.010 µg-eq/mL) at the first sample time point (30 minutes post-dose) and below quantifiable limits at 24 hours [33].

In a second study, 18 female rabbits received a single intravaginal dose of 0.5 mL of tenofovir gel containing 1% w/v tenofovir (5 mg tenofovir per animal; 50 µCi/kg), while a further 18 rabbits received a single intravaginal dose of 0.5 mL of tenofovir gel containing 3% w/v 9- [(R)-2-(phosphonomethoxy)propyl]adenine monohydrate (PMPA) (15 mg PMPA per animal; 50 µCi/kg). Six animals from each group were sacrificed at each of 0.5, 4, and 24 hours post dose. The majority of the administered dose was recovered in urine (15 - 38%) or cagewash (28 - 36%), suggesting that the formulation leaked out of the vagina. Tissue concentrations of radioactivity were highest in vaginal tissue at 30 minutes post-dose, with substantial variability in actual tissue levels available (0.65 - 98.3 µg-eq/g for 1% tenofovir; 10.3 - 274 µg-eq/g for 3% tenofovir). Relatively high concentrations of radioactivity in intestinal tissues were attributed to ingestion of the formulation during grooming and poor total recovery in many animals was attributed to loss of formulation on fur and paws.

In addition, a non-blinded PK study evaluating the potential use of tenofovir in pre- and post-exposure prophylaxis[34], performed in HIV-1 infected subjects (nine men/13 women) using a 300 mg oral daily dose, showed that high extracellular and intracellular concentrations of tenofovir are achieved in the genital tract of men and women and provides encouraging data in support of tenofovir in pre- and post-exposure prophylaxis trials. After 24 hours, intracellular tenofovir genital tract to blood ratios in males for day 1 and 7 were 3.1±5.4 and 7.0±14.8, respectively. Tenofovir levels were significantly greater ($p<0.05$) in genital washes than blood at both day 1 and steady state in men and women. Tenofovir has a long half-life with intracellular levels sustained beyond 36 hours.

The systemic pharmacokinetics of 1% tenofovir gel has been evaluated in the HPTN 050 study among 25 women who applied tenofovir gel once or twice daily for 14 days. Serum plasma measurements were taken at enrolment at 0.5, 1, 2, 4, 6, 8 and 12 hours post dosing and again on day 14, 24 hours following the day 13 dose. The analysis of PK data was performed by measuring the area under the plasma concentration curve of tenofovir (AUC). Tenofovir accumulation was determined by comparing the median AUC on day 13 to the median AUC on day 0. In addition tenofovir AUC in sexually active versus sexually inactive cohorts was compared. Women on oral tenofovir prodrug (TDF) were excluded. Fourteen of 25 women (56%) had low, but detectable, serum tenofovir levels (lower limit of quantitation (LLOQ): 3.0 ng/mL) after 14 days of study gel use (Figure 1). The maximum serum tenofovir concentrations (C_{max}) ranged from 3.1 to 25.8ng/mL. For the woman with the highest observed peak level, 25.8ng/mL, this peak level occurred 2 hours following the dose; the level rapidly declined to 10.8ng/mL at 4 hours and was undetectable at 12 hours following the dose. Besides the outlier with the highest tenofovir level, the next highest C_{max} was 7.1ng/mL. No drug was detected in serum of any women at the 24 hour sampling point.

Levels for all women with measurable tenofovir levels in the blood are shown in figure 1 (14 of 25; LLOQ approximately 3.0 ng/mL [dotted line]). For reference the tenofovir level associated with the median 24 hour post-dose blood concentration following an oral 300 mg TDF dose is indicated with dashed line

Considering all women in the PK cohort, the median tenofovir C_{max} was 3.4 ng/mL (interquartile range: below LLOQ [3.0ng/ML] to 4.7ng/mL). The median C_{max} for all subjects (3.4ng/mL) corresponds to approximately 2.5% of the maximum (C_{max} at steady state = 135 ng/mL) and 7.2% of the minimum (C_{24} single dose median is approximately 47 ng/mL) blood concentrations at steady-state with 300 mg daily oral TDF dosing Figure 1.

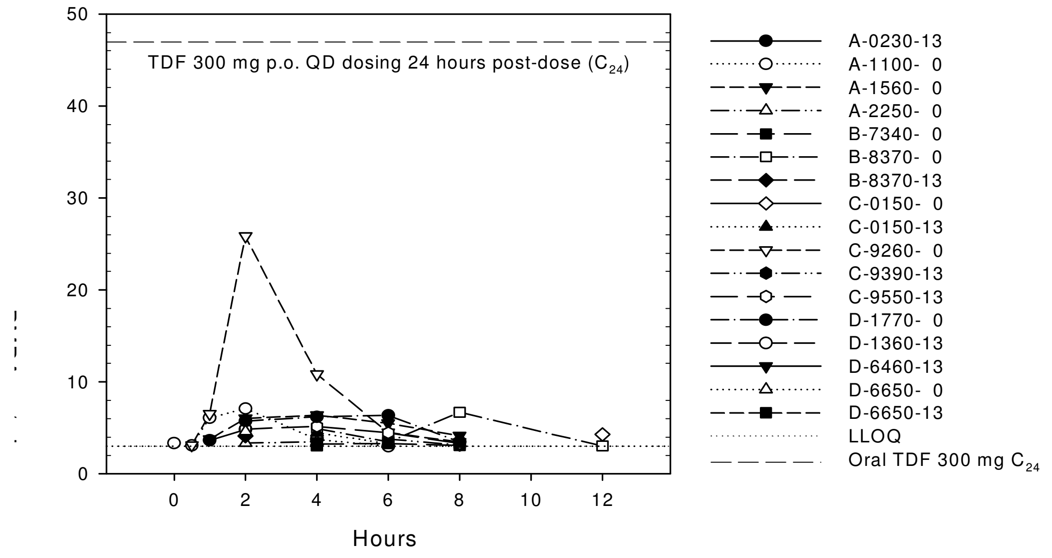

Figure 1: Tenofovir Blood Concentrations vs. Time after Vaginal Administration

Legend: First letter = Cohort; 4 digits = 4 digits of participant ID; Last digit = day of study; Cohort A – HIV uninfected/sexually abstinent; Cohort B - HIV uninfected/sexually active; Cohort C - HIV infected/sexually abstinent; Cohort D - HIV infected/sexually active

1.4 Dosing strategy of tenofovir gel

This is a Phase IIb, two-arm, randomised, double-blind, controlled trial comparing 1% tenofovir gel and a placebo gel. Despite significant advances in the microbicide field, the issue of timing of application for microbicides remains inconclusive in the absence of surrogate markers of protection or biological activity. Additionally, the timing of product insertion related to efficacy is affected by each product's mechanism of action. Hence, the definitive answers on timing of product insertion will only become available when a trial shows an efficacious product.

Two broad approaches have been proposed for the timing of insertion of tenofovir gel; daily dosing or coitally dependent dosing. There are groups of women who may benefit from each of these strategies. Daily dosing would require substantially more doses than coitally dependent use (with the concomitant increased costs), but it may be associated with higher adherence in some women. It has also been argued that daily dosing may have the converse effect of lower adherence in some women depending on various behavioural factors. With daily dosing, there may be no specific benefit to be gained from using a vaginal gel formulation as opposed to the standard oral formulation which would be more convenient and less expensive.

In the South African context, there is a specific need for a coitally-dependent microbicide (e.g. in certain young women and women with migrant partners due to the long periods without sex) to impact on high-risk sexual activity and the future course of the HIV pandemic.

Based on the available data, this trial proposes coitally-dependent application of tenofovir gel. The women will be encouraged to use the first dose of gel within 12 hours prior to coitus and insert a second dose of gel as soon as possible after coitus within 12 hours. Irrespective of the number of coital acts in a day, the women will be advised not to use more than two gel applications in a 24 hour period. The rationale for this decision, given the clear need for a coitally dependent efficacious microbicide, is based on the following::

- We hypothesize that a key mechanism of action of tenofovir gel is through its action of inhibiting viral replication in the CD4+ target cells in the lumen and in the layers of cells that make up the genital tract. Hence the achievement of adequate cellular (and tissue) concentrations of drug locally in the genital tract is considered important.
- The hypothesis that a single dose of tenofovir can prevent infection is supported by data from monkey models:
    - In macaques, 1% tenofovir gel used 24 hours before and up to 36 hours after challenge, in various dosing strategies, suggests protection.

- A single dose of 1% tenofovir given 15 minutes before challenge suggests protection. This has been shown in macaques with both vaginal and rectal dosing followed by vaginal and rectal challenge respectively

- Tissue levels in a monkey study were elevated early as 15 minutes following administration of 1% tenofovir gel. Peak concentrations were reached between 8 and 12 hours. At 24 hours post dose, the tissue concentrations observed were well above those seen in the first 4 hours post-dose but the levels are starting to wane. At 48 hours, tissue levels were still above the level of detection.
- Based on the above data, we selected the window of up to 12 hours pre-exposure in order to maximize tissue concentrations at the time of first exposure to the virus.
- In animal models, virus replication is evident as early as 1 hour post-challenge - an animal study showed that SIV can enter the vaginal mucosa within 60 min of intravaginal exposure and is detectable in draining lymph nodes within 18 hours following intravaginal SIV exposure[35]. While cervical explant data suggest that infection may only be first detectable by p24 assays in the tissues about 30 hours after exposure to challenge virus, even when mimicking disrupted genital tract epithelium. Hence, sustained tenofovir levels may be required for at least 48 hours post-inoculation to prevent viral replication.
- To decrease the likelihood of breakthrough infection due to waning levels of tenofovir within this window of opportunity to prevent replication, we have added a second dose of gel to be administered within 12 hours of exposure. While a single dose may provide adequate levels for this 48 hour period, the second dose is considered a valuable component of the dosing strategy to provide sustained levels of tenofovir over the critical first few days post-exposure. This second dose extends in time the “theoretical” protection provided by the presence of tenofovir in genital tract cells and tissues.
- The dosing strategy has been limited to not more than two doses in one day because the available human safety data on tenofovir gel are limited to two doses per day.

2 STUDY SETTING

This study will be conducted at two Clinical Research Sites in KwaZulu-Natal, South Africa and will enrol women at high risk of HIV infection in Durban and Vulindlela.

2.1 CAPRISA Clinical Research Site: Vulindlela

The Vulindlela Clinical Research Site is situated in a rural community with approximately 400,000 residents in the KwaZulu-Natal midlands, about 150 km north-west of Durban. Primary Health Care (PHC) services are provided through seven clinics in the district. These nurse-managed services provide antenatal care, family planning, childhood immunization, STI treatment, minor ailment care, tuberculosis treatment and HIV Voluntary Counselling and Testing (VCT). The closest referral hospitals are Grey’s and Edendale. The CAPRISA Clinical Research Site in Vulindlela adjoins the Mafakathini PHC Clinic.

HIV incidence in Vulindlela

A prospective cohort study (CAPRISA 050) has been conducted in Vulindlela with the aim of estimating rates of HIV seroincidence among women targeted for inclusion in this microbicide trial. Sexually active HIV uninfected women utilizing the Family Planning Clinic (FPC) and Antenatal Clinic (ANC) at the Vulindlela PHC facilities were enrolled in this study. Between March 2004 and April 2006 a total of 782 women were screened and HIV prevalence at baseline in this cohort was 30.2% (95%CI 26.7-33.9%). The average enrolment rate per month was 27 women. After 290 person years of follow up the HIV incidence rate in this cohort was 7.3 (95% CI 4.1-10.3)/100 person years.

ANC HIV surveys

The prevalence of HIV infection in pregnant women in Vulindlela has increased from 32.4% (95%CI 27.6-37.6%) in 2001 to 42.7% (95% CI 38.4-46.8%) in 2004. The age-specific prevalence for the 2001-2004 ANC survey in Vulindlela is presented in Table 2.

During this period of 4 years, most of the ANC attendees were less than 25 years of age; with 56.9% in this age range in 2001, 65.0% in 2002, 58.7% in 2003 and 65.6% in 2004. Of significance is that in 2004, 39% of the ANC attendees were younger than 19 years with the youngest being 12 years of age and the HIV prevalence in this age group was an alarming 26.7%. In 2004, the age specific prevalence was 54.7% among 20-24 year old women and 66.3% among 25-29 year old women. An increase from 31.2% (2001) to

42.9% (2002) to 66.0% (2003) was demonstrated in the 25-29 year old women. These data underscore the high and growing HIV prevalence and incidence rates in young, sexually active women under the age of 30 in this community.

Table 2: Age specific, Crude and Standardised HIV Prevalence 2001-2004 in Vulindlela ANC Attendees

	**2001**		**2002**		**2003**		**2004**	
**Age group**	**n/N**	**HIV Prev %(95%CI)**	**n/N**	**HIV Prev %(95%CI)**	**n/N**	**HIV Prev %(95%CI)**	**n/N**	**HIV prev %(95%CI)**
**<20**	6/41	14.6 (6.1-29.8)	41/ 159	25.8 (19.4-33.5)	13/67	19.4 (11.1-31.2)	54/202	26.7 (20.9-33.5)
**20-24**	2454	44.4 (31.1-58.5)	49/107	45.8 (36.2-55.7)	29/65	44.6 (32.5–57.4)	87/159	54.7 (46.6-62.5)
**25-29**	10/32	31.3 (16.8-50.2)	36/64	42.9 (32.3-54.2)	33/50	66.0 (51.1–78.4)	55/63	66.3 (55.0-76.1)
**30-34**	3/21	14.3 (3.8-37.4)	8/36	22.2 (10.7-40.0)	9/21	42.9 (22.6–65.6)	35/65	53.8 (41.1-66.1)
**>35**	3/19	15.8 (4.2-40.5)	6/23	26.1 (11.1-48.7)	8/22	36.4 (18.1–59.2)	4/41	9.8 (3.2-24.1)
**Missing**	67/182	36.8 (29.9-44.3)	¼	25.0 (1.3-78.1)	-	-	0/2	-
**Crude prevalence**	**113/349**	**32.4 (27.6-37.6)**	**141/413**	**34.1 (29.6-39.0)**	**92/225**	**40.9 (34.5-47.7)**	**235/552**	**42.6 (38.4-46.8)**
**Standardised prevalence***		**27.9 (27.2-28.6)**		**35.5 (34.8-36.3)**		**43.5 (42.7-44.3)**		**47.1 (46.3-47.9*)**

* Standardised to the age structure of the National Department of Health HIV seroprevalence survey population in 2004

2.2 CAPRISA Clinical Research Site: eThekwini

The CAPRISA eThekwini Clinical Research site is located adjacent to the Prince Cyril Zulu Communicable Disease Centre (CDC), a designated PHC of the Durban City Health Department, for the diagnosis and treatment of STIs and tuberculosis. The clinic is conveniently situated in the Warwick triangle in the metropolitan region of Durban which serves as the nucleus of the public transportation with the central bus, “minibus” taxi station and rail station all within a 500 metre radius of the clinic building. This clinic is readily accessible in terms of the transport infrastructure. This clinic provides free STI and tuberculosis treatment. Annually, approximately 40 000 cases of STIs are treated at this clinic, approximately 36 000 of which are new cases. The majority of the STI patients accessing these facilities are self-referred either symptomatic with genital ulceration and/or vaginal discharge syndrome or as contacts of patients with a diagnosis of a STI and include both males and females. Given the high prevalence of HIV infection in South Africa and the strong association between STIs and HIV acquisition, these patients are at an increased risk of acquiring and transmitting HIV through sex[(36)]. Between July and December 2005, as part of a provider initiated HIV testing programme we tested 1190 women for HIV infection. The HIV prevalence was 54.7% with the highest prevalence in the 30-34 and 35-39 age groups (Table 3).

Table 3: Age-Specific Prevalence of HIV Infection Amongst Female eThekwini Clinic Attendees in Durban July 2005- December 2005

Age (years)	Number positive	Prevalence (%) (95% CI)
≤19	50	38.2 (29.9-47.1)
20-24	182	42.6 (37.9- 47.5)
25-29	204	63.4 (57.8-68.6)
30-34	126	78.3 (70.9-84.2)
35-39	53	68.8 (57.1-78.6)
>= 40	36	50.0 (38.1-61.9)
Total	651/1190	54.7 (51.8-57.6)

<u>HIV prevalence and incidence</u>

The CAPRISA 051 study currently being conducted at the eThekwini Clinical Research site in preparation for the proposed microbicide trial (CAPRISA 004) has screened 1259 women attending the STI clinic. The HIV prevalence in this cohort was 59.3% and the average enrolment rate of HIV negative women per month is 19. After 52 person years of follow up the HIV incidence rate in this cohort is 5.8 per 100 women-years (95% CI 0-12.3). A second existing cohort of high risk women (CAPRISA 002) was established by screening a total of 776 women with an HIV prevalence of 59.4%. The retention rate among HIV-negative women who were

followed over 19 months was 87.8% and the HIV incidence rate is 7.9 per 100 person-years (95% CI 4.1-11.6). The age specific HIV incidence rate in the CAPRISA 002 cohort was highest in the 18-19 year age group, but with a wide confidence interval (Table 4).

Table 4: Age-Specific Prevalence of HIV Infection Amongst a Cohort of High Risk Women (CAPRISA 002)

Age group (years)	HIV prevalence (%)	Incident infections / new cases	Person Years	Incidence Rate (95% CI)
18-19	53.4	3	16.2	18.6 (0.0 – 37.6)
20-24	67.7	2	32.6	6.1 (0.0 – 14.5)
25-29	73.0	0	24.8	0
30-39	62.2	7	61.0	11.5 (3.0 – 20.0)
40-49	39.1	5	63.4	7.8 (0.9 – 14.1)
50+	29.0	0	20.2	0
Total	59.4	17	216	7.9 (4.1 – 11.6)

Data on the incidence and prevalence of HIV from the three CAPRISA sero-incidence studies is summarised below (Table 5).

Table 5: Summary of Three CAPRISA Sero-Incidence Studies (CAPRISA 050/051 and CAPRISA 002)

	Vulindlela (CAP050)	eThekwini (CAP051)	Durban (CAP002)
Number screened	782	1259	776
HIV prevalence	30.2	59.3	59.4
Average number enrolled per month	27	19	31
Person-years of follow up	290	52	216
Incidence / 100 person years	7.3	5.8	7.9

3. STUDY OBJECTIVES AND DESIGN

3.1 Primary Objective

- To evaluate the effectiveness and safety of a candidate vaginal microbicide, tenofovir gel, when applied intravaginally by women, in preventing sexually transmitted HIV infection.

3.2 Secondary Objectives

- To assess the impact, if any, of tenofovir gel on the incidence rate of deep epithelial disruption
- To assess the impact, if any, of tenofovir gel on viral load in women who become infected with HIV during the trial.
- To assess tenofovir resistance in HIV seroconvertors in the trial
- To ascertain the impact, if any, of tenofovir gel on pregnancy rates and outcomes
- To assess the impact, if any, of product hold at study exit on HIV infection rates and tenofovir resistance

3.3 Ancillary Objectives

- To assess the impact, if any, of tenofovir gel in preventing sexually transmitted infections including herpes simplex virus type 2 (HSV-2) and human papillomavirus (HPV) infections.

3.4 Study Overview

3.4.1 Design

This is a Phase IIb, two-arm, double-blind, randomised, controlled trial comparing 1% tenofovir gel, with a placebo gel among **1250** sexually active, women at high risk for sexually transmitted HIV infection in South Africa.

3.4.2 Accrual and Follow-up

The anticipated total study duration is approximately 30 months, with accrual requiring approximately 18 months and follow-up continuing until 92 incident HIV infections are observed, which is expected approximately 12 months after the end of the accrual period. Accrual may be altered following reviews by

the Data and Safety Monitoring Board (DSMB). The Protocol Team will be advised as to whether the study should proceed as designed, should proceed with design modifications, or should be discontinued. It is estimated that 92 HIV endpoints are very likely to accrue within 30 months of study initiation. In the event that 92 HIV endpoints are not observed within 30 months of initiation of the trial, the advice of the DSMB will be sought. Specifically, the DSMB will be requested to advise on whether the trial should be stopped for futility or continue beyond 30 months post study initiation until 92 HIV endpoints have been observed.

3.4.3 Enrolment and Randomisation
As described more fully in section 4.4, potential study participants will be screened for eligibility and eligible participants will be enrolled in the study within 30 days of screening. At each of the two sites, eligible participants who provide informed consent to take part in the trial will be assigned randomly to either tenofovir gel, or placebo gel in a 1:1 ratio.

3.4.4 Summary of Assessments
Enrolled participants will complete monthly follow-up visits for the duration of their participation (a minimum of 6 months and a maximum of 30 months) (Appendix I). At each monthly visit, participants will complete an interval medical history, HIV testing, and pregnancy testing. HIV/STI risk reduction counselling messages and condoms will be provided by counsellors trained to administer consistent prevention messages. Similarly, pregnancy prevention counselling will be provided by trained staff. The assigned study product and counselling on product adherence will be provided at monthly visits by trained staff. At each monthly visit, participants will be instructed to bring back any product. The pharmacist (or authorized designee) will record the amount of product returned before destruction. At each monthly visit participants will also undergo an interviewer-administered structured interview to ascertain key HIV risk behaviours. All scheduled and participant-initiated study visits will be documented. Study visits will take place at the CAPRISA Vulindlela Clinical Research Site and the CAPRISA eThekwini Clinical Research Site.

3.4.5 Safety Assessments
While safety is assessed as part of each monthly medical history and examination, detailed safety assessments are undertaken additionally during screening, enrolment and at months 3, 12, 24 (for those participants who have the opportunity to reach 24 months) during follow up, at study exit and additionally if indicated. At these visits, blood samples will be collected for urea, electrolytes, creatinine, liver function tests, full blood count, calcium and phosphate levels (See Appendix II for details). These visits will also include a pelvic examination including naked eye examination of the external genitalia and speculum examination of the vagina and cervix. Colposcopy will be performed if indicated. These visits will also include blood for storage of serum and plasma for the potential post-trial assessments of activity against STIs and cytobrush specimens for storage of cervical cells for potential post-trial assessments for markers of safety, risk exposure, product adherence and tenofovir resistance. For symptoms experienced between scheduled visits, the participant will be instructed to report to the Study Sites as soon as possible.

3.4.6 Outcome Assessment
The primary HIV endpoint is defined as two positive Ribonucleic Acid (RNA) Polymerase Chain Reaction (PCR) tests from independent samples obtained post-randomisation. The HIV testing algorithm at baseline, and at each monthly follow-up visits is included in Appendix III.

<u>At screening</u>, participants will undergo two rapid tests for HIV. Those who are negative on both tests and meet all eligibility criteria as assessed within a 30-day period since screening will be enrolled in the study.

<u>At enrolment</u> participants will not be re-tested for HIV, but plasma samples taken at this time will be stored for future testing among those who seroconvert early in the trial in order to confirm that seroconversion occurred post-randomisation.

<u>At each monthly visit</u>, participants will be tested for HIV with two rapid HIV tests. Participants with two negative rapid tests will continue follow-up in the study. If both tests are not negative i.e. either of these tests is positive or indeterminate, then the participant is considered a suspected seroconvertor. RNA PCR testing will be performed on suspected seroconvertors to confirm HIV status and a follow-up visit will be scheduled

for a week later. If the RNA PCR test is positive, a second blood sample will be drawn to confirm HIV status using RNA PCR during the scheduled visit a week later and another follow-up visit will be scheduled for a week thereafter to present results. Western blots and ELISAs will be performed on all suspected seroconvertors to provide additional confirmatory information on the presence / absence of infection. The primary HIV endpoint of HIV infection is defined as two positive PCR tests from independent samples. Participants will continue using gel product until HIV serostatus is confirmed by the algorithm in Appendix III.

Upon request, participants can be tested for HIV between scheduled study visits if they feel they have been exposed or are experiencing symptoms of HIV infection.

At the end of the study all new HIV infections will be confirmed by RNA PCR on stored plasma for quality assurance purposes and samples of plasma stored from enrolment will also be tested by RNA PCR to confirm that seroconversion occurred post-randomisation. Non-incident cases will be excluded from the analysis for the primary objective.

Participants who become infected with HIV will be offered counselling and referral, as appropriate, to the CAPRISA acute infection study (CAPRISA 002) and/or treatment programmes or other HIV/AIDS care services.

Post trial assessments – all participants will be asked to come back for one more visit 2 months after the study exit visit for outstanding results and one final HIV test and blood specimen collection for confirmatory HIV testing and potential resistance testing.

Note: Any new infections established at this visit will not be included as study endpoints. This post-trial visit is a safety visit that is part of monitoring HIV infection and tenofovir resistance following product withdrawal. All participants identified as HIV infected during this visit will be referred to the CAPRISA 002 Acute Infection study for follow-up.

All HBsAg positive patients will in addition have a blood sample collected for Hepatitis B and liver function tests. Any participants with significant changes in liver function since study exit will be referred to a health care provider for further follow-up.

3.4.7 Clinical management of non-study conditions

Participants who are found to have an STI or other treatable reproductive tract infection at a scheduled or participant-initiated visit will be provided counselling and clinical care in accordance with the South African Department of Health guidelines, free of charge. Participants with STIs will be encouraged to refer their partners for treatment.

Participants who become pregnant during the study will discontinue product use while they are pregnant. Pregnant women will be advised to continue with their follow up visits. When these participants no longer have a positive pregnancy test, the pregnancy outcome will be documented and they will be re-started on their allocated study product.

Participants who are found to be co-infected with Hepatitis B will be closely monitored. Clinical monitoring will include a review of the liver function tests done routinely through the study. Any participant needing further treatment for hepatitis B will be referred to a health care provider for further follow-up.

4 STUDY POPULATION

The study will include up to 1250-sexually active South African women at high risk for HIV infection.

4.1 Inclusion Criteria

While the HIV epidemic affects all age groups, young women have particularly high HIV incidence rates and hence the decision to focus this study on adult women up to 40 years of age. Women must meet all of the

following criteria at enrolment (by self-report, unless otherwise indicated) in order to be eligible for inclusion in the study:

- Age 18-40 years (inclusive)
- Able and willing to provide written informed consent to be screened for, and to enrol in, the study (Appendix IVa and b).
- Able and willing to provide adequate locator information for study retention purposes.
- Sexually active, defined as having had vaginal intercourse at least twice in the past 30 days prior to screening.
- HIV negative on testing performed by study staff within 30 days of enrolment (see algorithm Appendix III).
- Have a negative pregnancy test which was performed by study staff within 21 days of enrolment[a]
- Agree to use a non-barrier form of contraceptive
- Agree to adhere to study visits and procedures.

4.2 Exclusion Criteria

Women who meet any of the following criteria (by self-report, unless otherwise indicated) will be excluded from the study:

- History of adverse reaction to latex.
- Plans any of the following during the next 16 to 30 months (depending the anticipated date of study completion):
  - To travel away from the study site for more than 30 consecutive days.
  - To relocate away from the study site.
  - To become pregnant
  - To enrol in any other study of an investigational product or behaviour modification related to HIV prevention.
- Has a creatinine clearance <50ml/min, as estimated using the method of Cockcroft and Gault[(37)].
- **Has active Hepatitis B infection**
- Has a clinically apparent pelvic examination finding (observed by study staff) involving deep epithelial disruption. Otherwise eligible participants with pelvic examination findings involving deep epithelial disruption may proceed with enrolment after the findings have resolved and the inclusion/exclusion are met.
- Has in the past year participated in any research related to any vaginally applied product/s.
- Has current STI symptoms and/or other reproductive tract infection requiring treatment, as assessed by study staff. Otherwise eligible participants diagnosed during screening with infection(s) requiring treatment may be enrolled provided that treatment has commenced.
- Has any other condition that, based on the opinion of the Investigator or designee, would preclude provision of informed consent, make participation in the study unsafe, complicate interpretation of study outcome data, or otherwise interfere with achieving the study objectives.

4.3 Recruitment, Screening, and Enrolment

4.3.1 Sources for study participants

The high HIV prevalence and incidence rates in Vulindlela and Durban make these populations suitable for microbicide effectiveness trials as these populations; additionally these populations could derive substantial public health benefit in reduced HIV transmission from an effective microbicide. In Vulindlela, potential study participants will be recruited from among clients utilizing the primary health care clinics in Vulindlela, existing CAPRISA studies such as CAPRISA 050 and 051 as well as through community-based outreach activities, as appropriate. At the eThekwini site, potential participants will be enrolled from existing CAPRISA studies such as CAPRISA 051 and 002, from STI clients utilizing the Prince Cyril Zulu CDC and from community outreach activities in Durban. Consenting participants currently enrolled in the HIV seroincidence studies (CAPRISA 050 and 051) will be terminated from those studies and then offered the opportunity to be screened for this study.

---

[a] *Note: Breastfeeding is not exclusionary*

4.3.2 Cohort recruitment and accrual

Eligible participants will be enrolled over approximately 18 months. At regular intervals, the Principal Investigators in consultation with the study team will assess progress in accrual and retention at each of the two sites and may reallocate enrolment numbers and targets across the sites, as deemed necessary to achieve the goals of this trial efficiently.

4.3.3 Screening and enrolment

Eligibility for the study will be assessed in a step-wise manner at the study Screening Part 1 and Screening Part 2 Visits (described in Sections 6.2 and 6.3 respectively). Although all required procedures may be completed in two visits, additional visits may be conducted if needed or the two screening visits may be combined, where appropriate. Regardless of the number of visits required, all screening and enrolment procedures will be completed within a 30-day period. If a participant is not enrolled within 30 days of providing informed consent for screening, the participant will be re-consented for screening and the screening process will be repeated, in which case the results of the last screening prior to enrolment will be considered applicable for trial purposes.

<u>Screening Part 1</u>

Screening visit 1 will be completed in a step wise manner. Firstly, potential participants at the Clinical Research Sites will be invited to screen for the microbicide study and asked to provide written informed consent for screening (Appendix IVa). Potential study participants will be assigned a screening number, receive pre-test counselling, and two rapid HIV tests will be performed. . Post test counselling will be provided and those testing positive or indeterminate on at least one rapid test will be referred to an HIV/AIDS treatment programme. Participants who present with two negative test results from HIV testing conducted within 14 days of volunteering for screening for CAPRISA 004 will proceed with the screening process without repeating HIV rapid testing. HIV testing may be repeated prior to enrolment if >30 days have elapsed between the HIV rapid test result and the date of enrolment. If both HIV test results are negative, the potential participant will be invited to continue with the screening process and will be asked to provide demographic information, behavioural eligibility information, locator information, blood for creatinine levels and undergo urine pregnancy testing. Potential participants will also be evaluated by research staff for STI symptoms and will be offered syndromic treatment as per South African Department of Health guidelines. Participants deemed eligible based on the above procedures will then be invited for the Screening Part 2 visit.

<u>Screening Part 2</u>

Potential participants will be informed of their Screening Part 1 creatinine test results and if they continue to be eligible, they will undergo a physical and pelvic examination. Potential participants with pelvic examination findings involving deep epithelial disruption may only be enrolled after the deep epithelial disruption findings have resolved, provided they meet all other eligibility criteria. Otherwise eligible participants with clinical features suggestive of a STI may be enrolled after STI treatment is initiated.

<u>Enrolment</u>

Women who meet all the study eligibility criteria will be requested to provide their written informed consent for participation in the trial and thereafter enrolled in the study. Consent for specimen storage will also be sought (Appendix IVc). Blood will be drawn for haematology, liver function tests, blood chemistry tests, serology, hepatitis B assays and PBMC, serum and plasma archive (Appendix II).

At enrolment and throughout the trial, enrolled participants will be provided with:

- HIV risk reduction counselling and supplies of male condoms
- Contraception counselling and provision of contraceptive methods as needed
- Supplies of the assigned study product with counselling on product use, importance of product adherence and importance of not sharing product.
- Instructions to contact study staff with questions about the study, requests for additional counselling, requests for additional condoms and study product, requests for contraception, as needed, and/or reports of AEs.

4.4 Co-Enrolment Guidelines

Participants in this study may not take part in other concurrent research studies that would interfere with the objectives of this microbicide study. The determination of whether participation in another study would be exclusionary for a given participant will be made by the Principal Investigators. Approved co-enrolment in other concurrent protocols will be documented.

4.5 Participant Retention

The target retention rate will be 90% per annum. The Protocol Team will track retention rates and take any required action to address below-target retention rates. If volunteers do not adhere to scheduled pre-enrolment visits, screening may be discontinued at the discretion of the protocol team. Once a participant is enrolled in the study, study staff will make every reasonable effort to retain her in follow-up. This may include obtaining and checking locator data, home visits, issuing telephonic and in-person reminders of scheduled visits, and maintaining a scheduler of enrolled participants as part of a strategy to achieve the target.

4.6 Participant Withdrawal

Participants may voluntarily withdraw from the study for any reason at any time. Designated study staff also may withdraw participants from the study in order to protect their safety and/or if they are unwilling or unable to comply with required study procedures, after consultation with the Principal Investigators. Participants also may be withdrawn if the study sponsors, South African Medicines Control Council (MCC), United States (US) FDA, the University of KwaZulu-Natal Biomedical Research Ethics Committee, or FHI Protection of Human Subjects Committee (PHSC) may terminate the study prior to its planned end date.

Every reasonable effort will be made to complete a final evaluation of participants who withdraw or are withdrawn from the study. Study staff will record the reason(s) for all withdrawals in participants' study records.

5 STUDY TREATMENT CONSIDERATIONS

5.1 Product Formulation

5.1.1 Tenofovir gel

Tenofovir gel is a clear, transparent, viscous gel at concentrations of 1% (w/w) formulated in purified water with edentate disodium, citric acid, glycerin, methylparaben, propylparaben, hydroxyethylcellulose (HEC), and pH adjusted to 4-5. The gel will be administered as a 4mL dose. (See the Investigator's Brochure for detailed information on tenofovir gel)

5.1.2 Placebo Gel

The placebo gel (known as the 'universal' placebo gel) is formulated to minimize any possible effects — negative or positive — on study endpoints. It is isotonic to avoid epithelial cell swelling or dehydration. It is formulated at a pH of 4-5 but has minimal buffering capacity. When mixed with an equal volume of semen, the placebo gel induced only a trivial decrease in semen pH (from 7.8 to 7.7). The placebo gel contains HEC as a gelling agent, and its viscosity is comparable to that of tenofovir gel. HEC does not have anti-HIV properties. The gel contains sorbic acid as a preservative. Sorbic acid has no anti-HIV activity and is readily metabolized by human cells.

The placebo gel formulation was found to be non-irritating in a 10-day test for vaginal irritation in rabbits (BioSyn, Inc., personal communication). The formulation also showed minimal toxicity toward human vaginal epithelial cell monolayers in vitro (Thomas Moench, personal communication[b]). When tested in a mouse HSV-2 vaginal challenge model, the placebo afforded no protection when compared to no treatment or when compared to pre-treatment with phosphate buffered saline. The placebo also did not enhance susceptibility to HSV-2 when administered 12 hours before vaginal challenge. In contrast, N-9 and other detergent microbicides tested in the same protocol caused a 10-25 fold increase in susceptibility (Thomas Moench, personal communication[b]). In a phase I clinical trial conducted in the United States of America (USA) to

[b] Thomas Moench, MD. ReProtect, Inc, 703 Stags Head Road, Baltimore, MD 21286 USA

assess the safety of the placebo gel, no serious or unexpected AEs were reported following twice daily intravaginal application for 14 days in healthy sexually abstinent women[38]

5.2 Product Use Regimen

Trained staff will instruct participants in proper methods of storing and applying the assigned study product. This instruction will take place in advance of randomisation to minimize any potential bias. Participants will be instructed to insert one dose (the entire contents of one applicator) of product into the vagina up to 12 hours before each act of vaginal intercourse (intercourse may take place immediately after product insertion) and insert a second dose as soon as possible after coitus but within 12 hours. In the event that product is not inserted prior to coitus, the participants will be advised on the importance of inserting the post-coital dose as soon as possible within 12 hours after coitus. Irrespective of the number of coital acts in a day, participants will be advised not to use more than two applications in a 24 hour period. They will also be advised to:

- Only apply the assigned product vaginally.
- Not douche or otherwise clean the vagina, or insert other objects or vaginal products, for 2 hours after gel insertion. If a women plans to douche after coitus, she will be advised to insert the gel after douching.
- Not use other participant's study product.
- Not distribute their study product to other women.
- Not alter their study product in any way
- Properly store their study products (in a cool dry place out of direct sunlight)
- To use study product whether or not a condom is used.

As part of risk reduction counselling, participants will also be informed of the increased risk of HIV acquisition associated with anal sex compared with vaginal sex and will be encouraged not to engage in anal sex and to use a condom when anal sex cannot be avoided.

5.3 Product Management

5.3.1 Supply

CONRAD will provide the study products (tenofovir gel and placebo gel). All study gel will be produced, filled and packaged under Good Manufacturing Practices (GMP) conditions. The delivery volume, microbial limit, chemical and physical properties of the pre-filled applicators will be verified by the packager prior to shipping the clinical supplies. CONRAD will supervise the clinical supply operational procedures and review the GMP documents before authorizing shipment of the supplies to the clinical site.

The study products will be packaged in single-use opaque applicators containing approximately 4 ml of study gel. Each applicator will be individually wrapped and labelled. The products will be labelled such that the participant, investigator, monitor, data managers/analysts and sponsor (except CONRAD's Product Development Coordinator) will be blinded as to the identity of the product contained therein.

Ten individually wrapped applicators will be packaged in a box with an outside label and will be sealed with tamper-evident tape. Six randomisation groups (3 placebo and 3 tenofovir gel) will be prepared. Each gel applicator overwrap and outer box label will contain an alphanumeric variable assigned to each of the six groups, which the pharmacists on site will be able to decipher. The pharmacists on site will be blinded as to which of the three alphanumeric variables is active product and which is placebo.

The Study Pharmacist will obtain study products from CONRAD according to ordering instructions provided by CONRAD. The Study Pharmacist will maintain full accountability records in accordance with Good Clinical Practice (GCP) and legal requirements. These will include study participant logs as well as stock records for each randomisation group.

5.3.2 Storage

Drug product will be stored at controlled room temperature with excursions permitted from 15°C-30°C until required for administration. It should be stored away from direct sunlight.

5.3.3 Dispensing
Study product will be dispensed by designated study pharmacists to enrolled study participants as per Standard Operating Procedures (SOPs) in quantities expected to be sufficient until the participant's next monthly follow-up visit. In the event that a participant needs additional supplies between visits, she will be instructed to contact the study site to request additional supplies. Study participants will be asked to return all previously dispensed applicators at each visit. All returned applicators will then be disposed of in accordance with Good Pharmacy Practice.

5.3.4 Accountability
Complete records documenting receipt, inventory, dispensation, return and destruction will be maintained in accordance with Good Clinical Practice and US FDA requirements. These will include study participant logs as well as stock records for each randomisation group. Study participants will be asked to return all previously dispensed applicators at each visit. The number of used and unused applicators returned will be logged for each participant at each study visit.

5.4 Adherence Counselling
Adherence counselling will be provided to study participants upon enrolment and additionally at each study visit. Counselling will address such topics as participant-centred strategies to remember to use the products before and after sex, to ensure the availability of the products both in the home and away from home; and to identify and discuss various challenges and situations that may impede product use. Counselling also will include reminders to contact study staff with questions about product use and requests for additional supplies. For participants who have adherence problems, every effort will be made to identify adherence strategies to increase their rates of product use throughout the course of the study.

5.5 Adherence Assessment
Data on adherence to the product use regimen will be collected monthly via brief interviewer-administered instruments. These instruments will ascertain participants' frequency of sexual intercourse, condom use, product use and timing thereof in relation to coitus. The Protocol Team will monitor adherence rates over time, and adherence counselling methods will be updated and implemented systematically if needed to address lower-than expected rates. Additionally, genital specimens collected during the trial, as per procedures in the study MOP at study months 3, 12, 24 (for those participants who have the opportunity to reach 24 months), study exit and from suspected seroconvertors will be archived for potential post-trial analysis of cellular levels of tenofovir if there is utility in this potential marker of product adherence to enhance interpretation of the results of the trial.

5.6 Discontinuation of Product
Study participants will be discontinued from product use by Principal Investigators and designees in the event that they experience a Serious Adverse Event (SAE) that is judged by the study clinician or designee to be probably or definitely related to product use (see Section 7.3). Participants who become pregnant will discontinue product use and will only resume product use when their pregnancy test reverts to negative. The Principal Investigators and designees also may at their discretion discontinue product use — temporarily or permanently — among participants who:
- Experience an AE judged to be related to product use.
- Have a pelvic examination finding involving deep epithelial disruption that is not resolving.
- Are unable or unwilling to comply with required study procedures (including the strict guidelines on product sharing).
- Otherwise might be put at undue risk to their safety and well-being by continuing product use

Product use will be discontinued when a participant has been deemed to have reached the primary endpoint of HIV infection by the algorithm in appendix III.

For participants who discontinue product use, every effort will be made to complete all protocol-specified follow-up visits and procedures (except study product dispensing procedures) with these participants. The

study clinician or designee will document all changes in product application regimen, and the reason for the change, on applicable Case Report Forms (CRFs).

5.7 Concomitant Medications

Enrolled study participants may continue use of all concomitant medications, including prescription, non-prescription, traditional, and other preparations during this study, except for vaginal products. As noted in Section 5.2, participants will be encouraged to avoid douching and the use of vaginally-applied medications/preparations. All concomitant medications used by participants throughout the course of the study will be reported on applicable CRFs.

6 STUDY PROCEDURES (See Appendix I: Schedule of Evaluations)

Study staff will be trained to conduct study procedures in a standardised manner. SOPs and a Manual of Procedures (MOP) will guide this process.

6.1 Targeted |Recruitment

Study staff may conduct targeted recruitment, by focusing study outreach and recruitment efforts on women likely to be between 18 and 40 years of age. All possible efforts will be made to maintain the confidentiality of eligibility criteria, so as not to encourage artificial responses from volunteers being screened or to encourage them to change their behaviour in order to be eligible for the study.

6.2 Screening Part 1 Visit (up to day -30)

If all the required procedures in Screening Part 1 cannot be completed in a single visit, then multiple visits may be conducted if necessary. For potential participants who do not meet the study eligibility criteria, the screening process will be discontinued when ineligibility is determined.

Screening 1 will be completed in a stepwise manner. The first step includes the provision of introductory study information and obtaining written informed consent for screening procedures. HIV testing including pre and post test counselling will be done and only HIV negative participants will continue with the screening process. If participants have had an HIV test conducted within 14 days of this screening visit and are in possession of their test results, HIV testing will not be repeated as part of this screening visit but may be repeated prior to enrolment if >30 days have elapsed between HIV testing and date of enrolment. The following procedures will be completed:

6.2.1 Administrative, Behavioural, and Regulatory Procedures

- Assignment of a screening number
- Informed consent for screening
- Collection of the following:
    - Demographic information
    - Locator information
    - Behavioural eligibility information
    - Contraceptive eligibility and pregnancy-intentions assessment
    - HIV/STI risk reduction counselling and provision of condoms

6.2.2 Clinical Procedures

- Assessing for STIs and other genitourinary symptoms requiring treatment
- Blood draw

6.2.3 Laboratory Procedures

- HIV rapid testing
- Urine pregnancy testing
- Creatinine level

6.3 Screening Part 2

Screening Part 2 and Screening Part 1 may be combined. If all the required procedures in Screening Part 2 cannot be completed in a single visit, then multiple visits may be conducted if necessary.

6.3.1 Administrative, Behavioural, and Regulatory Procedures

- Update locator information
- Confirm eligibility

6.3.2 Clinical Procedures

- Focused medical and menstrual history and ascertainment of concomitant medications
- Physical examination
- Pelvic examination (and if needed, colposcopy)

6.4 Enrolment Visit (day 0)

The enrolment visit will only be commenced for participants who are found to be eligible. Written informed consent for study participation will be obtained before any enrolment (or "on-study") procedures are conducted.

6.4.1 Administrative, Behavioural, and Regulatory Procedures

- Informed consent for enrolment and stored specimens
- Assign a participant identification (PID) number
- Update locator information
- Behavioural risk assessment
- Contraceptive counselling
- HIV/STI risk reduction counselling and provision of condoms
- Product adherence counselling

6.4.2 Clinical Procedures

- Physical examination
- Pelvic examination if indicated
- Review/update pre-existing conditions
- Inventory and documentation of concomitant medications
- Blood draw

6.4.3 Laboratory Procedures

- Urine pregnancy testing if no negative pregnancy test result in last 21 days prior to enrolment
- Serum, plasma and PBMC archive
- Haematology – full blood count
- Chemistry – creatinine, urea and electrolytes, calcium and phosphate
- Liver function tests
- Hepatitis B assay

6.4.4 Pharmacy Procedures

- Provision of assigned study gel and instructions
- Update accountability log

6.5 Follow-up Visits

Monthly follow-up visits are scheduled throughout the study follow-up period on a 28-day schedule. The visit window around each of these visits is 14 days on either side. In addition to the regular monthly follow-up requirements, additional procedures are done at quarterly visits. For participants who do not complete scheduled visits within the allowable window, the visit will be considered "missed" and relevant CRFs will be completed to document the missed visit. However, for participants who miss quarterly visits, the pelvic examination and behavioural interviews specified to take place at these visits will be conducted at the participants' next visit. The same will apply to participants who miss visits 3, 12 and 24 where certain

assessments are done. Participants who become pregnant during the study will be followed as usual, but product will be withheld until they have a negative pregnancy test. For participants who become HIV infected during the study, product will be discontinued once diagnosis is confirmed by our testing algorithm. HIV seroconvertors in the trial will be maintained in follow-up until 3 months after seroconversion, when a final specimen of blood will be obtained for them for the assessment of viral load and tenofovir resistance assays.

6.5.1 Administrative, Behavioural, and Regulatory Procedures

- Locator information update:
  - At all visits and contacts
- Behavioural and product adherence assessment:
  - At all visits (note: quarterly visits will be more detailed)
  - At study exit
- HIV pre- and post-test counselling:
  - Monthly
  - At study exit
  - Additionally when clinically indicated
- HIV/STI risk reduction counselling:
  - At all visits and contacts
- Distribution of condoms:
  - At all visits and contacts
- Contraceptive counselling
  - All visits
- Product sharing and acceptability assessment
  - At study exit
- Provide test results
  - if applicable

6.5.2 Pharmacy Procedures

- Supply of assigned study product
  - Monthly (except at study exit)
  - Additionally when indicated
- Updating accountability log
  - Monthly (except at study exit)
  - Additionally when indicated
- Collection of residual unused and used study product from participant
  - Monthly
  - study exit

6.5.3 Clinical Procedures

- Interval (i.e., since last visit) medical and menstrual history including detailed intermenstrual bleeding history and AE assessment and concomitant medication review
  - Monthly
  - Additionally when indicated
- Pelvic examination (and, if needed, colposcopy)
  - Quarterly
  - Study exit
  - Additionally when indicated
- Genital specimen collection
  - Month 3, 12, 24 (for all participants having the opportunity to reach 24 months)
  - If indicated, at the time of suspected or confirmed seroconversion
  - study exit
- Blood draw
  - Month 3, 12, 24 (for all participants having the opportunity to reach 24 months) and study exit
  - Additionally when clinically indicated

6.5.4 Laboratory Procedures

- Urine pregnancy test:
  - Monthly
  - Additionally when clinically indicated
- HIV rapid tests
  - Monthly,and at study exit.
  - Additionally when clinically indicated
- HIV confirmatory RNA PCR
  - if indicated
- HIV confirmatory Western Blot and Elisa
  - if indicated
- Safety bloods (see appendix II for tests)
  - Month 3, 12, 24 (for all participants having the opportunity to reach 24 months) and study exit
  - Additionally when clinically indicated
- Hepatitis B assays
  - At study exit
- Serum, plasma and PBMC archive.
  - Month 3, 12, 24 (for all participants having the opportunity to reach 24 months) and at study exit
- Processing and storage of genital specimens
  - month 3, 12, 24 (for all participants having the opportunity to reach 24 months) and exit

6.6 Interim Contacts and Visits

Interim visits may be performed at any time during the study, for a number of reasons, which include, but may not be limited to, the following:

- For administrative reasons, e.g., a participant may have questions for study staff or may need to re-schedule a follow-up visit.
- For product-related reasons, e.g., a participant may need additional study product or want to discuss problems with adherence to product use.
- In response to AEs. When interim contacts or visits are completed in response to participant reports of AEs, study staff will assess the reported event clinically and provide or refer the participant to appropriate medical care
- For interim STI counselling and or treatment in response to STI symptoms.
- For interim HIV counselling and testing in response to presumed exposure to HIV or seroconversion symptoms.
- Contraception counselling and / or provision
- To provide participants with the results of confirmatory HIV test results, per the algorithm in Appendix III.
- For other reasons at participant request.

6.7 Study Exit Visit

6.7.1 Administrative and Behavioural Procedures

- Locator information update
- Behavioural and product adherence assessment
- HIV pre- and post-test counselling
- HIV/STI risk reduction counselling and distribution of condoms
- Product sharing and acceptability assessment

6.7.2 Clinical Procedures

- Interval medical history, AE assessment and concomitant medication review
- Pelvic examination (and, if needed, colposcopy)
- Genital specimen collection
- Safety bloods (see appendix II for tests)

6.7.3 Pharmacy Procedures

- Collection of residual unused and used study product from participant
- Updating accountability log

6.7.4 Laboratory Procedures
- HIV rapid tests
- HIV confirmatory RNA PCR
  - If indicated
- HIV confirmatory Western Blot
  - if indicated
- HIV confirmatory ELISA
- Haematology and Serum Chemistry (see Appendix II for tests)
- Hepatitis B assays
- Safety bloods (see appendix II for tests)
- Serum, plasma and PBMC archive.
- Processing and storage of genital specimens

6.8 Post trial HIV test Visit
6.8.1 Clinical Procedures
- Blood draw for tenofovir resistance testing in suspected seroconvertors
- Genital specimen collection in suspected seroconvertors

6.8.2 Laboratory Procedures
- HIV rapid tests

6.9 Final Contact
To minimise tenofovir resistance, following product hold, a final post-trial contact visit will be conducted. All participants will be scheduled to return two months after product is withdrawn at study exit for HIV testing and for potential HIV resistance testing in suspected seroconvertors. The following participants will have their follow-up from their study exit completed at this visit :

- Participants who require confirmatory HIV testing
- Participants whose final safety bloods are abnormal
- Participants who have an outstanding AE at the time of the final visit
- Participants who are pregnant
- Participants who become HIV infected during the trial
- Enrolled participants who are Hepatitis B positive

7 SAFETY MONITORING AND ADVERSE EVENT REPORTING
7.1 Adverse Events and Reporting Requirements
An AE is defined as any untoward medical or social occurrence in a clinical research participant which may or may not have a causal relationship with the study product. Study product refers to tenofovir gel and the placebo gel, and the above-listed definition of an AE will be applied to both arms of the study beginning from the time of randomisation. New information regarding symptoms or conditions that occur during the screening period, but prior to the randomisation will be recorded in the participant's medical history as pre-existing conditions. All new or worsening symptoms or conditions that occur following randomisation will be considered AEs and will be recorded on the AE CRF.

7.2 Adverse Event Reporting
Study participants will be provided contact telephone numbers and instructed to contact a study clinician to report any AEs they may experience, except for life-threatening events, for which they will be instructed to seek immediate emergency care. Depending on the severity of the event, the clinician will instruct the participant to present to the study site (for more mild events) or to a hospital casualty department (for more serious events) for immediate evaluation. With appropriate permission of the participant, records from all non-study medical providers related to AEs will be obtained and required data elements will be recorded on study CRFs. All participants reporting an AE will be followed clinically, until the AE resolves (returns to baseline) or stabilizes. AEs that are ongoing at the time of study exit will be followed up for up to 30 days after study exit and then, if not resolved, will be referred to a health care provider for further follow-up.

The Investigator must determine the severity of the AE and document it on the appropriate CRF (AE Form). Each adverse event that the participant is aware of should be graded for severity using the following scale (DAIDS severity grading system ICH 6):

- **Mild:** participant was able to perform all normal activities.
- **Moderate:** the participant had to discontinue some activities due to the adverse event.
- **Severe:** the participant was incapacitated by the adverse event and unable to perform normal activities.
- **Life-threatening** participant experienced extreme limitation in activity, significant assistance required; significant medical intervention / therapy required, hospitalisation or hospice - care probable.

An AE **does not** include:

- Pre-existing diseases or conditions present or detected prior to start of study drug administration that do not worsen.
- Medical or surgical procedures (e.g. surgery, endoscopy, tooth extraction, transfusion); the condition that leads to the procedure is an adverse event.
- Situations where an untoward medical occurrence has not occurred (e.g. hospitalization for elective surgery, social and/or convenience admissions).

The Investigator must determine the relationship of the AE to the product under investigation and document on the appropriate CRF (AE Form). For each AE, an assessment of the relatedness to the study drug will be made using the criteria and scale as outlined in the study MOP.

All AEs will be captured regardless of the association or otherwise to the study product and reported on the AE CRF in accordance with study specific procedures. All AE reports will contain at least the date the AE occurred, a brief description of the event, the relationship to study drug, the study drug action taken, the outcome, date resolved, and the seriousness of the event.

7.2 Serious Adverse Event (SAE) Reporting

An SAE includes any experience that is fatal or life-threatening, results in persistent or significant disability/incapacity, requires or prolongs hospitalization, or is a congenital anomaly. A life-threatening AE means that the participant was, in the view of the designated study staff, at immediate risk of death from the condition as it occurred. Notification of deaths will be recorded by reflecting the medical condition that led to the death on the AE CRF and also reported on the SAE report. Reporting SAEs may require additional detailed reports and follow-up, depending upon the study clinician's estimate of a causal relationship between the study product and the AE(s), and whether the AE(s) is identified in nature, severity, and frequency in the Investigator's Brochure or other risk information supplied to the Investigator.

All serious adverse events will be reported to FHI within 24 hours of the study site becoming aware of the problem. Study staff will complete a FHI SAE Report Form and submit it electronically or by fax to:

Director, RA/QA
Family Health International
PO Box 13950
Research Triangle Park, NC 27709 USA
Phone: 1-919-405-1445
Fax: 1-919-544-1308

In cases in which a SAE Report Form cannot be submitted in writing within 24 hours, the designated study staff will report the SAE via telephone and the SAE Report Form will be completed as soon as possible after the verbal report. A study SOP will outline the detailed procedures for the reporting of SAEs to the ethics committees and regulatory bodies, in fulfilment of their reporting requirements.

7.3 Safety Monitoring

Designated study staff will be responsible for continuous close safety monitoring of all study participants. The study statisticians will prepare routine study progress reports for review by the Protocol Team. In addition, the study statisticians will prepare routine study progress reports which include reports of AEs experienced by study participants (blinded to treatment assignment) for review by the Protocol Safety Review Team

(PSRT). The membership, scope of responsibility, role and modus operandi of the PSRT will be outlined in the study MOP and will include safety monitoring of enrolled participants infected with Hepatitis B. PSRT members will meet in-person and/or via teleconference regularly throughout the period of study implementation. Any deaths of study participants or other SAEs must be reviewed and a decision taken by the PSRT with regard to whether a DSMB review is warranted. In addition to monitoring performed by the PSRT, the study DSMB will review the study data during the period of study implementation. Following its review of the trial, the DSMB may recommend that the study proceed as designed, proceed with design modifications, or be discontinued.

7.4 Data, Safety, Monitoring Board

An independent Data Safety Monitoring Board will be assembled for this study. The details for the operation and responsibilities of the DSMB will be defined in a separate DSMB Operational Plan, which will be included in the MOP. The Operational Plan will delineate the composition, duties, responsibilities and procedures of the DSMB, data required at each meeting as well as the analyses that will be conducted, i.e. interim comparisons of the efficacy and safety of the study products.

Interim monitoring reports will be generated by an independent statistician (who is not otherwise involved in the study) and submitted to the DSMB (some data that go to the DSMB will be "open" and available to the study team, like grouped data listed below). All reports created by the independent statistician will be distributed to the members of the DSMB only and will not be available to any study staff. DSMB reports will be kept in a locked file with restricted access.

A full interim review of the available data will be undertaken during the conduct of the study. These interim reviews will be triggered once 22 of the anticipated primary endpoints are reached and again once 68 of the anticipated primary endpoints are reached. The data to be reviewed will include:

- Accrual data
- Baseline data
- Retention data
- Safety data
- Quality assurance data
- Effectiveness data (The type I error rate will be controlled over the two planned interim analyses plus the final analysis using O'Brien-Fleming type boundaries with the Lan-Demets spending function.)
- Any other data requested by the DSMB

After each DSMB meeting, the Chairperson will issue a written report describing all recommendations. The DSMB could recommend that the study should proceed as designed, should proceed with design modifications, or should be discontinued due to any reason (safety, efficacy, futility, etc.).

8 STATISTICAL CONSIDERATIONS

8.1 Review of Study Design

This is a two-arm, double-blind, randomised, placebo controlled trial comparing 1% tenofovir gel with a placebo gel, conducted among up to 1250 women at high risk for HIV infection in South Africa. A total study duration of approximately 30 months is planned, with accrual requiring approximately 18 months and follow-up continuing until 92incident HIV infections are observed.

8.2 Endpoints

8.2.1 Primary Endpoints

The primary study objective is to evaluate the effectiveness and safety of a candidate vaginal microbicide, tenofovir gel, when applied intravaginally by women, in preventing sexually transmitted HIV infection. The primary endpoint of HIV infection will be assessed as described in Appendix III.

8.2.2 Secondary endpoints

Consistent with the study objectives listed in section 3.2, the following secondary endpoints will be assessed:

- deep epithelial disruption observed on pelvic examination

- pregnancy rates
- pregnancy outcomes, which will be assessed in the following categories: induced abortion; spontaneous abortion; stillbirth; live birth with a congenital anomaly; or live birth without a congenital anomaly.

For women who become infected with HIV during the trial, additional secondary endpoints will be assessed:

- viral load in the first available HIV positive blood specimen, and at 3 months after the first HIV positive specimen
- Tenofovir resistance assessed by resistance assay test performed on virus isolated from the first available HIV positive blood and genital specimen and 3 months after the first HIV positive specimen.
- Tenofovir resistance assessed by resistance assay test performed on virus isolated from participants testing HIV positive at the post trial assessment visit scheduled two months after study exit following product hold

8.2.3 Ancillary endpoint

Consistent with the ancillary objective listed in section 3.3, the following ancillary endpoints will be assessed

- The presence of STIs including HSV_2 and HPV, diagnosed through retrospective analysis of stored samples, including but not limited to, antibody detection, viral nucleic acid and viral culture.

8.3 Accrual, follow-up, and sample size

The HIV incidence in the current longitudinal studies are 7.9 per 100 person years in the CAPRISA 002 study, 5.8 per 100 person years in the eThekwini CAPRISA 051 study and 7.3 per 100 person years in the Vulindlela CAPRISA 050 study. We assume that the infection rate will be slightly lower than this in the placebo arm of the study, and have therefore assumed an incidence rate of **7** per 100 person years as the rate in the placebo group.

We plan to enrol up to 1250 women over approximately 18 months and to continue follow-up until 92 incident infections are observed (unless the DSMB recommends discontinuing the trial early, see section 7.4). This number of events is expected to provide 90% power to detect a 50% effect (using a two-sided alpha=0.05 significance level test). Assuming an HIV incidence rate of 7 per 100 person years and 10% annual loss to follow-up, the required number of incident infections (92) will be reached approximately 12 months after the end of the enrolment period, resulting in a total study duration of approximately 30 months. Sample size and power were calculated using NQUERY.

The chance of detecting various other levels of effectiveness levels ranging from 30% to 60% at the two-sided, α=0.05 significance level are provided in Table 6.

Table 6: Power to Detect Different Levels of Product Effectiveness at the Two-Sided α =0.05 Significance Level Assuming 68 Events are Observed

**Actual product effectiveness (reduction in risk for active gel relative to placebo gel)**	**Power to detect an effect (p<0.05)**
60%	**97%**
50%	**90%**
40%	**74%**
30%	**41%**

The power to detect an effect of tenofovir gel on the first secondary safety objective, the rate of deep epithelial disruptions, is provided in Table 7.

Table 7: Power to Detect Differences in Deep Epithelial Disruption Rates (Assumes Two-Sided Alpha of 0.05, 10% Annual Loss to Follow-up, 18 months of Recruitment and Total Study Duration of 30 Months, Sample Size of up to 1250)

Baseline Rate	Power to Detect a:		
(per 100 person years in placebo group)	2-fold increase	3-fold increase	4-fold increase
1	**45%**	**80**%	**>99**%
2.5	**83%**	>99%	>99%
5	**98%**	>99%	>99%

The sample size will be re-assessed approximately 2 months before the expected enrolment of the last participant, based on the observed event rate pooled across treatment arms. If it is determined at this time that additional participants will likely be required to yield the required number of incident HIV events (i.e.92) by the end of 30 months of follow-up, then consideration will be given to enrolling additional women, up to a maximum of 1500 women.

The Principal Investigators and protocol statisticians will periodically review the overall product adherence and HIV infection rate data (pooled across treatment arms) to determine if any protocol changes with respect to duration of follow-up should be implemented. For example, if recruitment rates are lower than expected and overall adherence or infection rates decrease with longer study participation, then the protocol may be amended to limit the maximum number of months of follow-up for participants.

8.4 Random Assignment and allocation concealment

A randomisation plan describing procedures for randomisation, allocation concealment, and blinding will be established prior to initiating the trial. A summary of the procedures is as follows: Enrolled participants will be assigned at random to one of the two study treatment arms in equal proportions. However, to facilitate blinding without greatly complicating product distribution logistics, each participant will be randomly assigned on one of six different groups (designated by an alphanumeric variable e.g., A, B, C, D, E and F) in a 1:1:1:1:1:1 allocation ratio. Three groups will correspond to the placebo gel and three to tenofovir gel.

The randomisation statistician, who is not otherwise involved in the study will randomly assign three of the alphanumeric variables to placebo and three to the active gel. This information will be sent to CONRAD for drug packaging and will be stored in a locked file cabinet (paper copy) and in a secured computer directory (electronic copy) at the CONRAD office. Except for key individuals involved in drug packaging, the treatment code (e.g., which 3 alphanumeric variables correspond to active drug and which 3 correspond to placebo) will not be known to anyone else. These treatment codes may only be revealed to the DSMB for planned interim analyses. Individual treatment assignments may also be revealed to the study investigators in the event that emergency unblinding of a study participant is required (see Section 8.5 for details). Any instances of unblinding, intentional or otherwise, will be documented in the study files. .

The randomisation list used to assign individual study participants to one of the six groups will be generated by a randomisation statistician who is not otherwise involved in the study. This statistician will use a randomly permuted block design, stratified by site. Two or more pre-specified block sizes will be recorded on a formal randomisation request form, but they will not be written in the protocol or communicated to the clinical staff in order to reduce the chance of the clinical staff anticipating the assignment of the next subject. Electronic copies of the randomisation schedule and the programs used to generate the randomisation schedule will be limited in access and password protected. Paper copies of the randomisation schedule will be locked in a secure location at the CAPRISA office, where no unauthorized study staff will have access to them.

The randomisation statistician will provide the study pharmacy at each site with sealed, opaque randomisation envelopes, sequentially labelled by participant identification number (PID). These envelopes will be assigned in sequential order to eligible study participants. Upon opening the envelope the pharmacist will add his or her name and signature as well as the time and date the envelope was opened. The treatment letter to which the participant was assigned will be known only to the pharmacist.

8.5 Blinding

Both study staff and participants will be blinded to treatment assignments. Blinding will be maintained until all data are entered into the study database, all primary study endpoint data have been cleaned and verified, preliminary analyses have been performed, and the data are ready for final analysis. If study data that are not part of the primary or secondary outcomes are still outstanding at the time of the proposed locking of the database, the database could be locked, and participants treatment assignments revealed, provided that the study statisticians and Principal Investigators agree that these data are not needed to perform the primary or secondary analyses. If necessary, the DSMB Chair may be asked to consider and make a determination on this request.

It is not anticipated that unblinding will be necessary for the provision of medical treatment or to otherwise protect the safety of study participants. In the event that an Investigator is concerned that a participant might be put at undue risk by continuing product use, the Principal Investigators or designee may discontinue product use by this participant, without knowledge of the actual treatment assignment. However, in the unlikely event that a study staff member feels that specific product knowledge is necessary to protect a participant's safety, he/she will notify the Principal Investigators and the protocol statisticians to consider and jointly rule upon the request. The DSMB Chair may also be asked to consider and make a determination on this request. If deemed necessary, then the procedures outlined in the randomisation, allocation concealment and unblinding plan will be followed for requesting and documenting unblinding of individual study participants.

All study participants will be administered a brief unblinding assessment at their study exit visits in which they will be asked to report which study product they think they received. A sample of study staff will complete a similar assessment after the close of each study site.

8.6 Data Analysis

An expanded Statistical Analysis Plan (SAP), covering both the final analysis and the planned interim analysis, will be finalised before the first participant is enrolled. The following is a summary of the planned analyses. Any deviations to be made from this summary plan will be documented in the detailed SAP.

All primary analyses will be performed on an intention to treat (ITT) basis. For the ITT analysis, patients will be analysed according to the treatment arm they were randomised to, even if the participant was off product or received the product they were not assigned for a period of time while in the study. The only participants excluded from this primary analysis population will be women without a post-randomisation HIV test result, and women whose stored baseline blood samples were later found to be HIV positive. All primary and secondary analyses will be two-sided and will be performed at the 0.05 level of significance (adjusted to account for the two planned interim analyses). Additional analyses could be performed on other study populations, for example an as treated or a per protocol population. These analyses will be described in the SAP.

Any key decisions regarding the timing of outcomes, the appropriateness of test statistics or model assumptions, the eligibility of participants to be included in the various populations or any other statistical issues will be made in a blind review meeting. At the blind review at least the following people will be present: protocol statisticians; Principal Investigators; medical officer; and data manager. Only after this group documents that all data are sufficiently clean and all decisions regarding individual participant outcomes have been made will the study be unblinded to the true randomisation assignments

To assess effectiveness and safety of tenofovir gel the cumulative probability of HIV will be calculated for each treatment group using the Kaplan-Meier method. The difference in survival curves will primarily be evaluated with a logrank test, stratified by site. The type I error rate will be controlled over the two planned interim analyses, as well as the final analysis, using O'Brien-Fleming type boundaries with the Lan-Demets spending function. Secondarily, proportional hazards regression models will be used to estimate the hazard rate ratio, along with a 95% confidence interval, controlling for site and selected baseline prognostic variables (to be identified in the SAP).

Date of HIV infection will be estimated as the midpoint between the last negative HIV test date and the first confirmed positive HIV test date. Participants who do not become HIV infected before their last study visit will be censored on the day of their negative HIV test. Time to HIV infection, in days, will be computed as the difference between the estimated date of HIV infection and the randomisation date, plus one. Time to censoring will be computed as the difference between the date of censoring and the randomisation date, plus one.

Kaplan Meier estimates of the cumulative probability of deep epithelial disruption will be compared between the tenofovir gel and the placebo group using logrank tests, stratified by site. AEs occurring during the study will be summarized in frequency tables (including both the number of each type of AE and the number of distinct participants with each type of AE), by body system and by treatment group. Details of secondary analyses, including pregnancy rates and outcomes, as well as viral load levels and tenofovir resistance in HIV seroconvertors will be outlined in the SAP.

8.7 Data Management

Data will be collected on one-ply case report forms (CRFs) which will be developed by the study team. All site study staff will be trained in the correct completion of CRFs. If data entered on the CRFs are taken from an external source (e.g., laboratory reports, patient records), the source documents will be maintained in the participant's medical chart or study file at the site, and will be available for review. The CRFs will be faxed into the database management system which is DataFax version 3.7.002 (or higher) running on Sun Solaris OS 5.8 (or higher). DataFax has optical character recognition (OCR) which will read the check boxes and numerical fields on the CRFs and store them in the study database. Any fields not recognized by the OCR system will be entered manually by the Data Encoders. Data encoders will verify all data by cross-checking the faxed version to what is entered into the database.

Queries arising during validation of the data will be recorded in quality control (QC) Reports sent to the sites on a regular basis. Any queries resulting in a change to the database will be documented and attached to the original CRF. The data management centre staff will perform periodic quality control and validation checks on the data. Database files will be password-protected and access to the files will be limited to authorised study staff members only. All data will be backed up at regular intervals, and backups will be stored in secure areas with limited access.

The original CRFs and the DataFax version of the CRFs and related documents will be stored securely at the sites and both during and after the completion of the study. At all sites the forms will be stored in locked cupboards in a secure room with restricted access. Upon completion of the study, the close-out site monitoring visit and finalisation of the database for analysis, the original forms will be bound and kept for long term storage. CRFs will not be destroyed without written permission from the sponsor.

A detailed data management plan will be included in the study MOP.

9 HUMAN SUBJECTS CONSIDERATIONS

9.1 Regulatory and Ethical Review

This study will be conducted under the oversight of the South African MCC in accordance with International Conference on Harmonization (ICH) standards of Good Clinical Practice (GCP). CAPRISA will be responsible for reporting study-related information to the South African MCC.

The study also will be conducted under the oversight of the University of KwaZulu-Natal Biomedical Research Ethics Committee in South Africa and the FHI Protection of Human Subjects Committee in the USA. The study will only be initiated after it has been approved by both ethics committees. The study will be conducted in accordance with all conditions of approval by the ethics committees.

9.2 Informed Consent

Written informed consent will be obtained from each study participant in English or Zulu prior to screening and enrolment, in accordance with 21 CFR Part 50 and ICH GCP guidelines. Participants will be provided

with copies of their informed consent forms if they are willing to receive them. An impartial witness is required for the entire informed consent process in any participant who is illiterate or whose literacy is limited. Documentation of the presence of a witness will be achieved through their signature on the informed consent document. Illiterate participants will indicate their consent via use of their mark (finger/thumb print) on the informed consent documents.

9.3 Risks

The study clinical procedures are similar to those experienced by women in routine gynaecological examinations. Study participants may experience discomfort when having pelvic examinations and/or undergoing phlebotomy for this study. During phlebotomy, participants may feel dizzy or faint, and/or develop a bruise, swelling, or infection where the needle is inserted. Participants may become embarrassed, worried, or anxious when completing their HIV-related interviews and/or receiving HIV/STI counselling. They also may become worried or anxious while waiting for their HIV test results or after receiving HIV-positive test results. Trained counsellors will be available to help participants deal with these feelings.

Study personnel will make every effort to protect participant privacy and confidentiality, but it is possible that participants may disclose their HIV status to non-study participants and could be treated unfairly or discriminated against, or could have problems being accepted by their families and/or communities. Participants also could have problems in their partner relationships associated with use or attempted use of condoms and/or the investigational products.

Data on participant risk behaviours and the occurrence of other potential social harms will be collected from all participants. The Protocol Team will monitor trends in risk behaviours over time based on these data, as well as the occurrence of social harms, and initiate any required follow-up action.

Available evidence on the safety and tolerability of 1% tenofovir gel from the HPTN 050 study indicates that the product is safe and well tolerated in both HIV negative (N=60) and HIV positive (N= 24) women. Administration of tenofovir gel intravaginally at 0.3% and 1% concentrations resulted in minimal local irritation and little or no systemic adverse effects were identified. Although 92% reported at least one AE, the majority (87%) were mild and limited to the genitourinary tract. The most common AEs experienced were genital pruritus (23%), applicator site bruising (17%), applicator site erythema (17%), vaginal discharge (15%), irregular menses (13%) and metrorrhagia (11%). Four severe AEs were reported, with only one, lower abdominal pain, thought to be product-related[31]. Therefore the risks associated with tenofovir gel are believed to be substantially less than those identified for systemic tenofovir use. In the HPTN 050 Phase I study of tenofovir gel, serum PK analysis in a subset of participants demonstrated that there is no clinically significant systemic toxicity. Fourteen of 24 women with PK results had low, but detectable, serum tenofovir levels.

It is not known what effect tenofovir gel could have on the HIV virus or HIV disease progression in HIV infected participants or their partners. There is a theoretical risk that tenofovir absorbed systemically from tenofovir gel could result in mutations of the HIV virus in participants who become infected with HIV during the study, or their partner, if the partner is infected with HIV. Limited resistance data from the HPTN 050 study show that no new resistance mutations evolved in plasma or cervicovaginal lavage specimens after 14 days of tenofovir gel use[31]. No participant had high level tenofovir mutations (e.g., K65R).

Some of the possible side effects of the study gel are dryness, burning, itching, cervical ulceration, abrasion, ecchymosis, erythema, sub epithelial and/or petechial haemorrhage, inflammation, or pain in the genital area.

The following side effects have been associated with the use of oral TDF[39]: upset stomach, vomiting, gas, loose stools, dizziness, abdominal pain, lack of energy, kidney damage or failure, inflammation or swelling and possible damage to the pancreas, shortness of breath, rash, low phosphate, allergic reaction, change in bone growth and strength, and exacerbations of Hepatitis B Virus (HBV) in patients co-infected with HIV and HBV. These patients will need to have hepatic function closely monitored and anti-Hep B therapy resumed if appropriate. The updated Investigators Brochure (3rd edition, dated 11 January 2008) also includes a warning for possible lactic acidosis/severe hepatomegaly with steatosis including fatalities that have been

reported with the use of nucleoside analogs. Viread is also now indicated for the treatment of chronic hepatitis B in adults at a dose of 300mg daily. Optimal duration of treatment is not known[40]. Since systemic absorption of the gel formulation is considerably lower than the oral or intravenous formulations, these side effects are unlikely in this study which will be using a gel formulation. Regardless, study participants will be monitored for AEs related to those observed for the oral formulation.

9.4 Benefits

There may be no direct benefits to participants in this study. However, participants and others may benefit in the future from information learned from this study. Specifically, information learned in this study may lead to the development of a safe and effective vaginal microbicide that prevents HIV infection.

Study participants will receive HIV and STI counselling and testing, a physical examination, and gynaecological assessments. Contraception will also be available to study participants. They will be provided syndromic STI treatment free-of-charge, and will be offered STI treatment for their partners. For other medical conditions identified as part of the study screening and/or follow-up procedures, participants will be referred to other sources of care available in their community. Study participants will also receive condoms and risk reduction counselling and will be reimbursed for transport and refreshment costs for each scheduled visit.

9.5 Access to HIV-related care

9.5.1 HIV counselling and testing

HIV counselling will be provided to all potential study participants who consent to undergo HIV screening to determine their eligibility for this study, and to all enrolled participants at each follow-up HIV testing time point. HIV test results will be provided with post-test counselling. Condoms will be provided to participants throughout the duration of their participation in the trial.

9.5.2 Care for participants identified as HIV-infected

Potential study participants who volunteer to undergo HIV testing as part of the study screening process may discover that they are HIV positive. Study staff will provide all HIV test results with post-test counselling. Potential study participant who have been identified as HIV positive will be referred to local AIDS treatment services. Such services include the CAPRISA–based as well as other local facilities that provide medical and psychosocial AIDS care and support.

HIV-uninfected study participants who become infected during follow-up will be referred to one of the long-term CAPRISA Acute Infection cohort studies, which have excellent provisions for care, antiretroviral therapy and support for those infected with HIV. For those who do not wish to continue in any of these studies post-seroconversion, they will be referred to their preferred AIDS care provider which could include the CAPRISA AIDS treatment programme, government or non-governmental AIDS care services for ongoing clinical management and care.

9.6 Community involvement and consultation:

The CAPRISA community programme (CP) has, through a consultative process, established CAPRISA Community Research Support Groups (CRSGs) at both the CAPRISA Sites where this study will be conducted. The CRSG membership includes local community leaders, traditional leaders, leadership of local HIV/AIDS organisations, previous study participants, local health service provider representatives and HIV positive local community members. The CAPRISA CP in partnership with the CRSG's will involve the community and local community based organisations in preparation for this trial. Specifically, the CAPRISA CP will inform, educate and mobilise the community to enhance community input into the research process. The local CRSGs in Vulindlela and eThekwini play an active role as an interface between the researchers and community members serving as advocates for the community's best interests and ensuring that the researchers are aware of any concerns within the community about the research being conducted. The CRSGs also play an important role in reviewing study educational materials, consent forms and Zulu translations of documents which will be shared with study participants.

9.7 Confidentiality

Every effort will be made to protect participant privacy and confidentiality to the extent permitted by law. Study-related information will be stored securely at the study site. All participant information will be stored in lockable file cabinets in areas with access limited to study staff. Data collection, process, and administrative forms, laboratory specimens, and other reports will be identified by a coded number only, to maintain participant confidentiality. All records that contain names or other personal identifiers, such as locator forms and informed consent forms, will be stored separately from study records identified by code number. All databases will be secured with password-protected access systems. Forms, lists, logbooks, appointment books, and any other listings that link PID numbers to other identifying information will be stored in a separate, locked file in an area with limited access.

Participants' study data, as identified by PID number only, will not be released without their written permission, except as necessary for review and monitoring by:

- Authorized study representatives
- South African MCC
- US FDA
- University of KwaZulu-Natal Biomedical Research Ethics Committee
- FHI's Protection of Human Subject Committee
- FHI Monitors and Auditors

9.8 Study Discontinuation

This study may be discontinued at any time by the South African MCC, US FDA, FHI PHSC, University of KwaZulu-Natal Biomedical Research Ethics Committee, or the Protocol Team (e.g., in response to recommendations from the DSMB or the study sponsor).

10 LABORATORY CONSIDERATIONS

The study laboratory plan will include the procedures for specimen management (e.g. chain of custody, handling, labelling and transport), assay procedures, proficiency testing and quality assurance procedures and specimen storage procedures.

10.1 Laboratory Specimens

The following types of specimens will be collected for testing:

- Urine for pregnancy testing
- Blood for haematology (full blood count [FBC]) and chemistry (liver and renal function testing, calcium and phosphate) (Appendix II).
- Blood for HIV testing by rapid tests confirmatory RNA PCR assays, Western blots and ELISAs
- Blood for HBV testing
- Blood and genital specimens from suspected seroconvertors for virus and tenofovir resistance assays
- Blood for PBMC, plasma and serum archive.
- Genital specimens for archive

All the above specimens will be collected with Good Clinical and Laboratory Practice standards and as described in the SOPs for collection of specimens.

10.2 On site testing

The study laboratory plan will detail the procedures to be followed for on-site testing as well as proficiency testing for all on-site testing (i.e. urine pregnancy tests and HIV rapid tests).

10.3 Collection and shipping of specimens

All specimens (bloods, urine and genital) will be collected according to methods described in the MOP and SOPs for proper collection, processing, labelling, and transport of specimens to the laboratories conducting the assays.

10.4 Specimen Storage for Quality Assurance and Potential Future Research Testing

Serum, plasma, PBMC and genital specimens will be stored for potential post-trial assessments for activity against STIs, markers of safety, risk exposure, product adherence and tenofovir resistance. In addition, stored plasma will be used for retrospective RNA PCR or Western blot testing to confirm whether early incident cases of HIV infection during the trial occurred post-randomisation. Where possible, stored specimens will be re-tested to assess the validity of unusual or unexpected assays results. For those participants who do not consent to long-term storage of their specimens, any residual specimens will be destroyed at the end of the study after all protocol-required and quality assurance testing has been completed.

10.5 Laboratory Quality Control and Quality Assurance Procedures

The laboratories involved in the study will follow the quality assurance and quality control procedures outlined in the study laboratory plan. For the on-site tests, the quality assurance personnel from the CAPRISA laboratory will conduct periodic visits to the Clinical Research Sites in Vulindlela and eThekwini to assess the implementation of on-site quality control procedures, including maintenance of laboratory testing equipment, use of appropriate reagents, proficiency testing records and quality checks of on-site testing procedures.

11 ADMINISTRATIVE PROCEDURES

11.1 Protocol Compliance

The study will be conducted in full compliance with the protocol. Amendments to the protocol will be required to follow an SOP which stipulates the levels of approval required prior to submission to regulatory bodies and the steps to be followed prior to implementation of a protocol amendment.

11.2 Protocol violations

A "protocol violation" is broadly defined as any departure from the procedures described in the study protocol. Protocol violations that may impact subject safety, affect the integrity of study data, affect subject's willingness to participate in the study, and/or provide evidence of wilful or knowing misconduct or non-compliance on the part of the site investigator(s) will be documented and reported, at minimum, to FHI. Protocol violations may be identified by any of the study staff or by the study monitor. The procedures for documenting protocol violations will be specified in the monitoring plan

Some examples of protocol violations include:

- Omission or inadequate administration of informed consent
- Inclusion/exclusion errors, including legal age limit
- Treatment errors: incorrect dispensation of study product
- Missing or incorrectly timed study procedures and assessments
- Failure to discontinue product use due to protocol criteria

In an emergency, the Investigator may make departures from the protocol to eliminate an apparent immediate hazard for a particular participant. In such a case, he/she will notify the Ethics Committee and FHI in writing as soon as possible and document reasons for the violation (unless solely caused by participant non-compliance such as not attending for study visits).

11.3 Quality Assurance and Study Monitoring

Quality assurance in the trial will be undertaken according to the Study Quality Assurance Plan which will be part of the study MOP. The Quality Assurance Plan will include ongoing monitoring of study progress and safety by the Protocol Team, study monitoring in accordance with ICH GCP guidelines by trained FHI monitoring staff, and independent quality assurance audits by FHI Regulatory Affairs/Quality Assurance staff and/or other outside QA contractors. The Investigators will allow study monitors to inspect study facilities and documentation (e.g., informed consent forms, clinic and laboratory records, other source documents, CRFs), as well as observe the performance of study procedures. The Investigators will also allow inspection of all study-related documentation by authorized representatives of the South Africa MCC, the FDA, and the study sponsors. A site visit log will be maintained at the study site to document all visits.

11.3.1 Study Monitoring

Study monitoring will be conducted by representatives of FHI who have received adequate training as monitors and specifically on this study. Monitoring shall commence shortly after enrolment of the first participants and at regular intervals thereafter. A site visit log will be maintained at the study site to document all visits. Monitor findings will be documented per FHI SOP. The Principal Investigators will be notified of the visit findings. If the monitor discovers issues related to safety, he/she is to report their findings immediately to the Principal Investigators or designee as well as to the project manager at FHI.

11.3.2 Auditing

FHI will conduct site visits and evaluation after at least 5 months of study conduct and then again about 6 months before the projected end of the study to evaluate study systems for quality control, monitoring of the study, documentation of the study, and overall control of the study.

11.4 Study Records

Complete, accurate, and current study records will be maintained and stored in a secure manner, throughout the study. All study records will be maintained for at least 5 years after the termination of the trial and extended to 2 years following the date of marketing approval for the study product for the indication in which it was studied and until there are no pending or contemplated marketing applications or at least 2 years have elapsed since the formal discontinuation of the development of the investigational product. Gilead Sciences or Conrad will inform the Principal Investigators if this storage period needs to be extended.

11.5 Use of Information and Publications

Presentation and publication of the results of this study will be governed by CAPRISA's publication policy.

12 REFERENCES

1. UNAIDS, WHO. AIDS Epidemic Update: December 2003: Joint United Nations Programme on HIV/AIDS and World Health Organization; 2003.
2. Department of Health, RSA. National HIV and syphilis antenatal sero-prevalence survey in South Africa 2003. Pretoria: Department of Health, Health Systems Research, Research Coordination and Epidemiology; 2004.
3. Lurie M, Williams BG, Gouws E. Circular Migration and Sexual Networking in rural KwaZulu/Natal: Implications for the Spread of HIV and other Sexually Transmitted Diseases. Health Transition review. 1997;7:15-24.
4. Abdool Karim Q, Abdool Karim SS. South Africa: Host to a new and emerging HIV epidemic. Sexually Transmitted Infections. 1999;75:139-40.
5. UNAIDS. AIDS Epidemic Update. December 2005. Geneva, Switzerland; 2005.
6. Abdool Karim Q, Stein Z. Women and HIV/AIDS: A global perspective. In: Goldman M, Hatch M, editors. Women and Health: Academic Press; 2000.
7. Nicolosi A, Correa Leite ML, Musicco M, Arici C, Gavazzeni G, Lazzarin A. The efficiency of male-to-female and female-to-male sexual transmission of the human immunodeficiency virus: a study of 730 stable couples. Italian Study Group on HIV Heterosexual Transmission. Epidemiology. 1994;5(6):570-5.
8. Williams BG, Gouws E, Colvin M, Sitas F, Ramjee G, Abdool Karim SS. Patterns of Infection: using age prevalence data to understand the epidemic of HIV in South Africa. South African Journal of Science. 2000;96:305-12.
9. Abdool Karim Q, Abdool Karim SS. Epidemiology of HIV infection in South Africa. AIDS. 1999;13:S4-S7.
10. Purnima M, Aggleton P. Gender and HIV/AIDS: What Do Men Have to Do With It? Current Sociology. 2001;49(6):23-37.
11. CDC. Update: barrier protection against HIV infection and other sexually transmitted diseases. MMWR. 1993;42:589-97.
12. Stone AB, Hitchcock PJ. Vaginal microbicides for preventing the sexual transmission of HIV. AIDS. 1994;8:S285-S93.
13. Elias CJ, Coggins C. Female controlled methods to prevent sexual transmission of HIV. AIDS. 1996;10:S43-S51.
14. Elias CJ, Heise LL. Challenges for the development of female-controlled vaginal microbicides. AIDS. 1994;8(1-9).
15. IWGVM. Recommendations for the development of vaginal microbicides. AIDS. 1996;10:1-6.
16. Roddy RE, Zekeng L, Ryan KA, Tamoufe U, Weir SS, Wong EL. A controlled trial of nonoxynol 9 film to reduce male-to-female transmission of sexually transmitted diseases. New England Journal of Medicine. 1998;339(8):504-10.
17. Richardson BA, Lavreys L, Martin HLJ, Stevens CE, Ngugi E, Mandaliya K, et al. Evaluation of a low-dose nonoxynol-9 gel for the prevention of sexually transmitted diseases: a randomized clinical trial. Sexually Transmitted Diseases. 2001;28(7):394-400.
18. Van Damme L, Ramjee G, Alary M, al. e. Effectiveness of COL-1492, a nonoxynol-9 vaginal gel, on HIV-1 transmission in female sex workers: a randomized controlled trial. Lancet. 2002;360(971-977).
19. De Clercq E, Holy A, Rosenberg I, Sakuma T, Balzarini J, Maudgal PC. A novel selective broad-spectrum anti-DNA virus agent. Nature. 1986 Oct 2-8;323(6087):464-7.
20. Balzarini J, Aquaro S, Perno CF, Witvrouw M, Holy A, De Clercq E. Activity of the (R)-enantiomers of 9-(2-phosphonylmethoxypropyl)-adenine and 9-(2-phosphonylmethoxypropyl)-2,6-diaminopurine against human immunodeficiency virus in different human cell systems. Biochemical and biophysical research communications. 1996 Feb 15;219(2):337-41.
21. Balzarini J, Holy A, Jindrich J, Naesens L, Snoeck R, Schols D, et al. Differential antiherpesvirus and antiretrovirus effects of the (S) and (R) enantiomers of acyclic nucleoside phosphonates: potent and selective in vitro and in vivo antiretrovirus activities of (R)-9-(2-phosphonomethoxypropyl)-2,6-diaminopurine. Antimicrobial agents and chemotherapy. 1993 Feb;37(2):332-8.
22. De Clercq E, Holy A, Rosenberg I. Efficacy of phosphonylmethoxyalkyl derivatives of adenine in experimental herpes simplex virus and vaccinia virus infections in vivo. Antimicrobial agents and chemotherapy. 1989 Feb;33(2):185-91.
23. De Clercq E. The acyclic nucleoside phosphonates from inception to clinical use: historical perspective. Antiviral research. 2007 Jul;75(1):1-13.
24. Gilead Sciences Inc. Investigators Brochure: Tenofovir Gel (GS-1278). Foster City, California Gilead Sciences, Inc; Second Edition, 31 March 2005.
25. Otten RA, Smith DK, Adams DR, Pullium JK, Jackson E, Kim CN, et al. Efficacy of postexposure prophylaxis after intravaginal exposure of pig-tailed macaques to a human-derived retrovirus (human immunodeficiency virus type 2). Journal of Virology. 2000;74:9771-5.
26. Tsai C-C, Follis K, Sabo A, Beck T, Grant R, Biscofberger N, et al. Prevention of SIV infection in macaques by (R)–9-(2 phosphonylmethoxypropyl) adenine. Science. 1995;270:1197-9.

27. Tsai C-C, Emau P, Follis KE, Beck TW, Benebiste R, Bischofberger N, et al. Effectiveness of Postinoculation (R)-9-(2-Phosphonylmethoxypropyl) Adenine Treatment for Prevention of Persistent Simian Immunodeficiency Virus SIVmne Infection Depends Critically on Timing of Initiation and Duration of Treatment; 1998.
28. Miller C, Rosenberg Z, Bischofberger N. Use of topical PMPA to prevent vaginal transmission of SIV. 9th International Conference on Antiviral Research; 1996; Japan; 1996.
29. Otten RA SD, Adams DR, Pullium JK, Jackson E, Kim CN, Jaffe H, Janssen R, Butera S, Folks TM. . Efficacy of postexposure prophylaxis after intravaginal exposure of pig-tailed macaques to a human-derived retrovirus (human immunodeficiency virus type 2). Journal of Virology. 2000;74:9771-5.
30. Shattock M. Protection of macaques against rectal SIV challenge by mucosally-applied PMPA. Microbicides 2006 Conference; 2006; Cape Town, South Africa; 2006. p. Abstract OA15.
31. Mayer KH, Maslankowski L, Gai F, El-Sadr W, Justman J, Kwiecien A, et al. Safety and tolerability of vaginal tenofovir gel in abstinent and sexually active HIV-infected and uninfected women. AIDS. 2006;20:543-51.
32. Hillier SL. Safety and acceptibiity of daily and coitally dependent use if 1% tenofovir over six months of use [Abstract BO12-655]. Microbicide 2008. New Delhi, India; 2008.
33. Shaw J-P. In vitro Metabolism of 14C-PMPA in Human and Animal Tissues.; 1996.
34. Vourvahis M, Tappouni H, Patterson K, Chen Y-C, Rezk N, Fiscus S, et al. A Pharmacologic Basis for the Use of Tenofovir In Pre- and Post-Exposure Prophylaxis: Intra (IC) and Extracellular (EC) Genital Tract (GT) Pharmacokinetics (PK) and Pharmacodynamics from First Dose to Steady State in HIV-1 Infected Men (M) and Women (W). CROI; 5-9 February 2006; Denver, Colorado; 5-9 February 2006.
35. Hu J, Gardner MB, Miller CJ. Simian Immunodeficiency virus rapidly penetrates the cervicovaginal mucosa after intravaginal inoculation and infects intraepithelial dendritic cells. . Journal of Virology. 2000;74(13):6087-95.
36. Kharsany ABM, Mahaber Y, Goga R, Sturm AW. Sexually transmitted diseases and human immunodeficiency virus infection among women attending a STD clinic. International Congress of Sexually Transmitted Disease Research; 11-14 July 1999; Denver Colorado; 11-14 July 1999.
37. Cockcroft DW, Gault MH. Prediction of creatinine clearance from serum creatinine. . Nephron. 1976;16(1):31-41.
38. Schwartz JL, Ballagh SA, Kwok C, Weiner DH, Mauck C, Callahan MM. Fourteen-Day Safety and Acceptability Study of Universal Placebo Gel. . Microbicides 2006 2006 April 24th -26th; Cape Town, South Africa; 2006.
39. Gilead Sciences Inc. Package Insert: Viread (tenofovir disoproxil fumarate). California: Gilead Sciences Inc.; June 2004.
40. Gilead Sciences Inc. Package Insert: Viread (tenofovir disoproxil fumarate). California: Gilead Sciences Inc.; January 2008.

APPENDICES

I. Schedule of Study Visits and Procedures
II. Safety Laboratory Evaluations to be Performed in Study Months 1-3
IIIa. HIV Antibody Testing Algorithm for screening
IIIb. HIV Antibody Testing Algorithm for Primary Endpoint Ascertainment at Follow-up Visits
IIIc: HIV Antibody Testing Algorithm for Suspected Acute Illness
IVa. Informed Consent Form for Screening Participants
IVb Informed Consent Form for Enrolling Participants
IVc Informed Consent Form for Specimen Storage and Possible Future Research Testing

Appendix I: Schedule of Evaluations

	Screening		Enrol	Monthly Follow-up																													Exit	
Procedure	Screening part 1 (up to 30 days)	Screening part 2 (up to 30 days)	Enrolment * (Day/month 0)	Month 1	Month 2	Month 3	Month 4	Month 5	Month 6	Month 7	Month 8	Month 9	Month 10	Month 11	Month 12	Month 13	Month 14	Month 15	Month 16[b]	Month 17[b]	Month 18[b]	Month 19	Month 20	Month 21	Month 22	Month 23	Month 24	Month 25	Month 26	Month 27	Month 28	Month 29	Study exit	Final contact
	V0	V1	V2	V3	V4	V5	V6	V7	V8	V9	V10	V11	V12	V13	V14	V15	V16	V17	V18	V19	V20	V21	V22	V23	V24	V25	V26	V27	V28	V29	V30	V31	V32	V33
Administrative, Behavioural and Regulatory Procedures																																		
Informed consent for screening	X																																	
Obtain screening number	X																																	
Informed consent for storage			X																															
Informed consent for enrolment			X																															
Demographic information	X																																	
Locator information	X	X	X	X	X	X	X	X	X	X	X	X	X	X	X	X	X	X	X	X	X	X	X	X	X	X	X	X	X	X	X	X	X	
Eligibility assessment	X	X	X																															
Counselling and condom supplies	X		X	X	X	X	X	X	X	X	X	X	X	X	X	X	X	X	X	X	X	X	X	X	X	X	X	X	X	X	X	X	X	
Document concomitant medication			X	0	0	0	0	0	0	0	0	0	0	0	0	0	0	0	0	0	0	0	0	0	0	0	0	0	0	0	0	0	X	
Obtain random assignment and PID			X																															
Baseline data collection			X																															
Sexual behaviour & product adherence assessment				X	X	X	X	X	X	X	X	X	X	X	X	X	X	X	X	X	X	X	X	X	X	X	X	X	X	X	X	X	X	
Product sharing & acceptability assessment																																	X	
Clinical Procedures																																		
Physical examination		X	X																															
Demonstration of product use			X																															
Focused medical and menstrual history		X		X	X	X	X	X	X	X	X	X	X	X	X	X	X	X	X	X	X	X	X	X	X	X	X	X	X	X	X	X	X	
Pelvic examination		X	0	0	0	X	0	0	X	0	0	X	0	0	X	0	0	X	0	0	X	0	0	X	0	0	X	0	0	X	0	X	X	
Genital specimen collection				0	0	X	0	0	0	0	0	0	0	0	X	0	0	0	0	0	0	0	0	0	0	0	X	0	0	0	0	0	X	
Colposcopy		0	0	0	0	0	0	0	0	0	0	0	0	0	0	0	0	0	0	0	0	0	0	0	0	0	0	0	0	0	0	0	0	
Scheduled blood draws for assays in lab	X		X	0	0	X	0	0	0	0	0	0	0	0	X	0	0	0	0	0	0	0	0	0	0	0	X	0	0	0	0	0	X	
Provide test results	X	X	(X)	X	X	X	X	X	X	X	X	X	X	X	X	X	X	X	X	X	X	X	X	X	X	X	X	X	X	X	X	X	X	X
Pharmacy procedures																																		
Provide study product & instructions			X	X	X	X	X	X	X	X	X	X	X	X	X	X	X	X	X	X	X	X	X	X	X	X	X	X	X	X	X	X		
Update accountability log			X	X	X	X	X	X	X	X	X	X	X	X	X	X	X	X	X	X	X	X	X	X	X	X	X	X	X	X	X	X	X	
Collection of used and unused product				X	X	X	X	X	X	X	X	X	X	X	X	X	X	X	X	X	X	X	X	X	X	X	X	X	X	X	X	X	X	
Perform laboratory evaluations:																																		
Urine pregnancy test	X		0[a]	X	X	X	X	X	X	X	X	X	X	X	X	X	X	X	X	X	X	X	X	X	X	X	X	X	X	X	X	X		
HIV serology (rapid tests)	X			X	X	X	X	X	X	X	X	X	X	X	X	X	X	X	X	X	X	X	X	X	X	X	X	X	X	X	X	X	X	X
HIV RNA PCR, Western blot and ELISA[e]	0			0	0	0	0	0	0	0	0	0	0	0	0	0	0	0	0	0	0	0	0	0	0	0	0	0	0	0	0	0	0	
PBMCs, Plasma and serum archive[c, e]			X			X									X												X						X	
Hepatitis B assays[e]			X																														X	
Creatinine level[e]	X					X									X												X						X	
Haematology[e] (full blood count) & Blood chemistry (LFT, ALT, U & E, CaPO4)			X			X									X												X						X	X[f]
Genital specimen for archive[c]				0	0	X	0	0	0	0	0	0	0	0	X	0	0	0	0	0	0	0	0	0	0	0	X	0	0	0	0	0	X	
Viral load in seroconvertors[d]																																		
Resistance testing in seroconvertors[d]																																		X
Amount of blood collected (mls):	85[e]	0	80	80[e]	80[e]	155	80[e]	80[e]	80[e]	80[e]	80[e]	80[e]	80[e]	80[e]	155	80[e]	80[e]	80[e]	80[e]	80[e]	80[e]	80[e]	80[e]	80[e]	80[e]	80[e]	155	80[e]	80[e]	80[e]	80[e]	80[e]	165	15

[a]If no negative pregnancy test result in last 21 days prior to enrolment

[b] The visit schedule for month 16, 17, and 18 visit will be repeated for each quarter after 24 months if necessary until study exit

[c]The stored serum, plasma and cytobrush specimens will be used for potential post-trial assessments for markers of safety, risk exposure, product adherence and tenofovir resistance. In addition, stored plasma will be used for retrospective RNA PCR testing to confirm whether incident cases of early HIV infection during the trial occurred post-randomisation. [d]Performed on first positive HIV rapid specimen and repeated at 3 months later.

[e]Blood volumes: Confirmatory tests (WB and ELISA) (20ml) (only if indicated), Creatinine (5 mls); Safety bloods (20 mls), HBV assays (10 mls); Storage (50 mls), 60 ml tenofovir resistance assays (only if indicated)

0 = if indicated

[f]HBsAg positive participants will have a liver function test performed

Appendix II: Safety Laboratory Evaluations

Performed at enrolment and at Study Months 3, 12, 24 and study exit

HEMATOLOGY TESTS
Full (Complete) blood count

Blood Chemistry
LIVER FUNCTION TESTS
Alkaline phosphatase
ALT
AST
Total bilirubin

RENAL FUNCTION TESTS
Urea
Creatinine
Serum electrolytes (Na+, K+, PO4-, Ca++)

Serum amylase

Appendix III: HIV Antibody Testing Algorithm

Screening

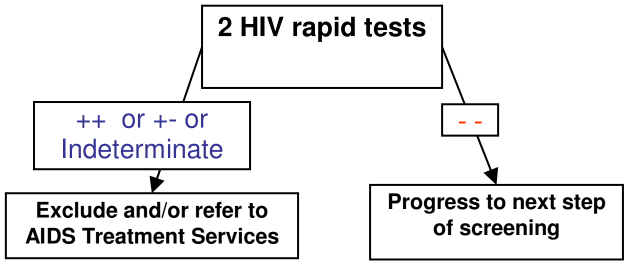

Monthly visits

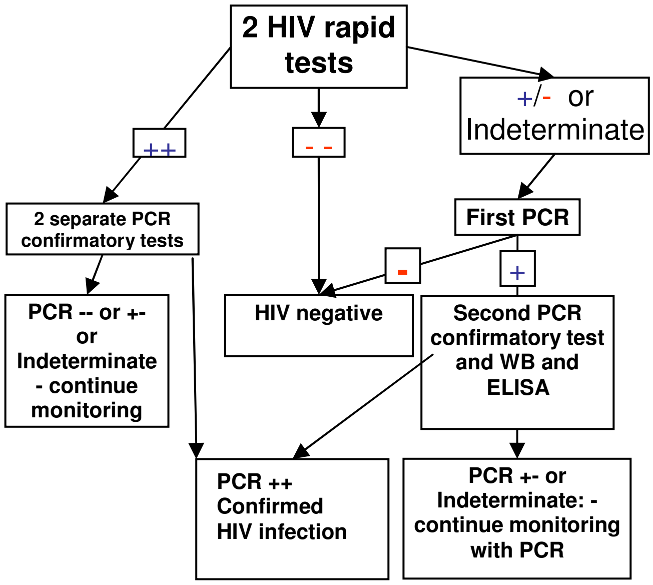

Appendix IVa: Informed Consent Form for Screening Participants (separate document)

CAPRISA 004

Phase IIb trial to assess the safety and effectiveness of the vaginal 1% tenofovir gel for the prevention of HIV infection in women in South Africa, Protocol Version 1.1, 20 April 2007

Version 1.8
27 March 2009

INFORMED CONSENT FORM FOR SCREENING

If the volunteer cannot read, this form must be read to the volunteer exactly as written, in the volunteer's language of choice, and a witness must sign this form to confirm that the correct information was given to the volunteer and that the volunteer freely consents to be in this study.

Biomedical Research Ethics Committee
Nelson R Mandela School of Medicine
Approved Version
2 0 APR 2009

Principal Investigators:
Professor Salim S Abdool Karim
Professor Quarraisha Abdool Karim
2nd Floor Doris Duke Medical Research Institute
Nelson R Mandela School of Medicine
Private Bag 7, Congella 4013
Durban, South Africa
PHONE: 031-260 4550

INTRODUCTION
You are being asked to volunteer for screening tests to find out if you are eligible for the research study named above. The purpose of this research study is to find out if a vaginal gel of an antiretroviral, called tenofovir gel can protect women from HIV. HIV is the virus that causes AIDS. This research study is for women who are sexually active and could therefore get HIV or other sexually transmitted infections (STIs). The screening tests include interview questions, urine and blood tests, a physical examination, and an examination of your genital tract. We will stop the screening procedures outlined below as soon as we find out that you are not eligible to participate in this trial.

YOUR PARTICIPATION IS VOLUNTARY
In order to be sure that you are informed about being in this research, we are asking you to read (or have read to you) this Consent Form in the language of your choice.

If you decide to participate, you will be asked to sign this Consent Form (or make your mark in front of a witness). We will give you a copy of this form to keep.
This Consent Form might contain some words that are unfamiliar to you. Please ask us to explain anything you may not understand.

Before you learn about the screening tests, it is important that you know the following:
- Your participation is entirely voluntary.
- You may decide not to have the screening tests, or to withdraw from the screening tests at any time. You will not lose the benefits of your routine medical care.
- If you decide not to have the screening tests, you can still join another research study, if one is available and you qualify.
- You are only being asked to have the screening tests at this time. Even if you agree to have the screening tests, you do not have to join the research study.

PURPOSE OF THE SCREENING TESTS
The purpose of the screening tests is to find out if you are eligible for this research study. Some people may not be able to join this tenofovir gel study because of information found during the screening tests.

PURPOSE OF THE STUDY FOR WHICH YOU ARE BEING SCREENED
The purpose of the study is to find out if tenofovir gel can protect women at high risk from getting infected with HIV from their sex partner/s.

Biomedical Research Ethics Committee
Nelson R Mandela School of Medicine
Approved Version
2 0 APR 2009

The study of tenofovir gel is "experimental." This means that we do not yet know all the effects of the gel, and we do not know if it protects women from getting HIV. Because we do not know whether tenofovir gel prevents HIV infection, the South African Medicines Control Council (MCC) ~~and the US Food and Drug~~ Administration (FDA) have not approved tenofovir gel for use in the general population for preventing HIV infection. The MCC however has given permission for this study to be conducted to test whether tenofovir gel can protect against HIV infection and to establish how safe it is to use for long periods of time. There is no guarantee that this study will show that tenofovir gel works in preventing HIV infection.

An earlier study with 84 women showed that tenofovir gel was safe to use in women who used Tenofovir gel once or twice per day. Tenofovir is detected in the blood at very low levels within 15 minutes of the gel application and is no longer detectable about 6 hours later. A recently completed study of 200 women from the USA and India reported that both daily and coitally dependent use of tenofovir gel was acceptable and safe.

This screening will determine who can be in the tenofovir gel study.

About 1250 women from Vulindlela and Durban will take part in this study. Women will be in the study for a minimum of 6 months to a maximum of 30 months, depending on when they join.

You will not be eligible to take part in the CAPRISA 004 study if you are currently taking part in another study investigating a product that is used in the vagina or any experimental product to prevent HIV infection. You will also not be eligible to take part in the CAPRISA 004 study if you have in the past 12 months participated in another study investigating a product that is used in the vagina or any experimental product to prevent HIV infection.

If you are eligible to participate in this study:

- It is important for your own safety that you do not participate in any other trial or study of vaginal gels or experimental products.
- The product that we are testing has not been proven for safety beyond 3 months. If a person uses gels or other study products from different studies at the same time and then has a problem that requires medical attention, we will not know whether the CAPRISA gel or other study product caused the problem.
- Also if a participant is using study products from different studies at the same time, we cannot assess which of the products is working.
- The study staff will use a set of questions to establish if you are taking part in another study. We will confirm what you tell us by checking your SA ID number with other studies in CAPRISA and other organisations doing HIV prevention research such as the South African Medical Research Council (MRC) to see if you are already participating in other HIV related research.
- If we find that you are in another study of an experimental vaginal product or any other HIV prevention interventions that involves an experimental product, the study staff will counsel you on why it may not be safe for you to participate in more than one study at the same time. Based on this information the study staff will make the decision and inform you on whether you are eligible to participate in CAPRISA 004.
- If you are eligible to join the CAPRISA 004 trial we will continue to check with other research organizations such as the MRC and other CAPRISA studies to establish if you have joined other research studies
- If we find that you are participating in another study of an experimental vaginal product or any other HIV prevention interventions that involves an experimental product, the study staff will counsel you on why it may not be safe for you to participate in more than one study at the same time. Based on this information the study staff will make the decision and inform you on whether you are eligible to continue participation in CAPRISA 004.
- When we check with other studies and organisations only information needed to verify your identity will be shared. No study related information will be disclosed such as your health status or behaviour.
- If we find that you are in another study, or have participated in another study in the past 12 months, that uses a vaginal product then you will not be eligible to take part in the CAPRISA 004.

PROCEDURES FOR SCREENING

If you agree to have the screening tests, you may have at least 2 visits at this facility. Your first visit will take place after you read, discuss, and sign this form. . If you are eligible after your first visit, your second visit will take place about 1 to 2 weeks from now.

Biomedical Research Ethics Committee
Nelson R Mandela School of Medicine
Approved Version

2 0 APR 2009

FIRST VISIT (TODAY)
If you agree to be in the screening, here is what will happen today. This process will take about one hour.

You will be counselled about HIV. If you've had a HIV test in the last 14 days of this screening visit and have your results to confirm this, you will not need to have a HIV test done today but if your are eligible for enrolment into this study and more than 30 days have elapsed since you had your HIV test we will need to repeat your HIV test prior to enrolment.

If you need to have an HIV test done then we will take some blood from your finger to test your blood for HIV. You will be told your result as soon as it is available today. You will talk with the study staff about the meaning of your result and how you feel about it.

If the test shows that you have HIV, you cannot take part in the tenofovir gel study. We will refer you to available sources of medical care and other services you may need. We will also tell you about other studies you may be eligible for at this organization.

Sometimes HIV tests are not clearly positive but also not negative. If that happens to you today, we will draw more blood from your arm with a needle to do additional HIV tests at the CAPRISA lab in Durban. The results from these additional tests will only be available in about one to two week's time. You will be given an appointment to return to this clinic to get this result. If we are not sure if you have HIV, you cannot participate in this study.

You must receive your HIV test results to be in the tenofovir gel research study

If your test results show that you do not have HIV you will continue with the screening.

You will answer questions about yourself, including where you live, your health, and your sexual practices.

You will give urine for a pregnancy test and learn the results. If you are pregnant you cannot take part in the tenofovir gel study. You will be referred for care as needed. You will be counselled on contraception and will be required to be on a non-barrier form of contraception which we could provide you with if needed.

We will take about 5 ml (1 teaspoon) of blood with a needle from your arm to check how well your kidneys are working. The blood will be sent to a laboratory in Durban to be tested, and it will take 1 or 2 weeks to learn the results. You will need to come back here to get the results.

You will be screened and counselled about other infections that may be passed during sex. These other infections may cause a discharge or ulcers in your genital tract and include syphilis, gonorrhea and chlamydia. If you are having health problems that may be due to these infections, we will give you medicine today to treat them.

You do not have to take any of these tests if you do not want to. However, if you want to find out if you are eligible for the tenofovir gel study, you will need to have these tests done.

SECOND VISIT (1 TO 2 WEEKS FROM TODAY)
If you are eligible after your first visit, you will come back to this clinic in 1 or 2 weeks for a second screening visit. The second visit will take about 90 minutes. Here is what will happen at that visit.

We will tell you your test results from your first visit, and what they mean.

If the tests show there are problems with your kidneys, you may not be eligible to participate in the tenofovir gel study. You will be referred to other sources of medical care.

You will be asked about any medications you are currently taking, previous medical problems you may have had and will have a physical examination, including an examination of your genital tract. During this examination, the study staff will look through a speculum for sexually transmitted infections. If you have any of these infections or abnormalities, we will tell you about them and give you medicine to treat them, if needed. If you have an infection that your partner may also have, you may bring him here for treatment that he may need too. This will be at no cost to you or your partner. If you do not have any of these infections, the staff will talk with you again about HIV and other infections passed during sex, and how to avoid these.

Biomedical Research Ethics Committee
Nelson R Mandela School of Medicine
Approved Version
20 APR 2009

ADDITIONAL VISITS
There is a chance that you will need to have more than 2 visits for the screening. All screening tests must be done within 30 days. If all tests are not done within 30 days, and you still want to find out if you are eligible for the tenofovir gel study, you will have to start the screening tests over from the beginning.

IF YOU ARE ELIGIBLE
We will look at the results of all these tests and questions to see if you are eligible for the tenofovir gel research study. If you are eligible, we will fully explain the study to you and answer any questions you have. If you decide to take part in the tenofovir gel research study, you will be asked to sign another consent form.

RISKS AND/OR DISCOMFORTS
You may feel discomfort or pain when your blood is drawn. You may feel dizzy or faint. You may have a bruise, swelling, or infection where the needle goes into your arm.

You may feel discomfort or pain during the examination of your genital tract.

You may become embarrassed, worried, or anxious when talking about your sexual practices, ways to protect against HIV and other infections passed during sex, and your test results.

You may become worried or anxious while waiting for your test results.

If you have HIV or other infections, you may become worried or anxious. A trained counsellor will help you deal with any feelings or questions you have.

We will make every effort to protect your privacy and confidentiality while you are having the screening tests, and if eligible for enrolment, all your follow-up visits. Your visits here will take place in private. However, it is possible that your partner, other family members or others may learn of your participation in this study for example through observing you visit the CAPRISA facility, or carrying a study bag or seeing where you store study product or through other means. These individual(s) may think you have HIV, or are at "high risk" for getting infected with HIV or they may get upset or angry with you for other reasons such as you are eligible for study participation and they are not, or you are receiving money for study participation, or you have access to gel and they do not or they have less control over you, or you have better access to information and better quality health services than they have. This may result in these people and possibly others treating you unfairly or discriminating against you for example making it difficult for you to get or keep a job, or being accepted by your family or community members or threaten you with violence. If you ever experience any of this discrimination or violence please you should feel free to contact the study staff telephonically or come to the clinic. You do not need to wait for your scheduled study visit to report this to study staff or seek assistance from staff. All of our staff have been trained to provide you with appropriate counselling and support. In addition we have a list of resources that can provide you with additional support or resources that are available to you to adequately assist you in dealing with these experiences.

While we respect your right to disclose your study participation with others it is hard to predict whether they will be supportive or not or threaten you with violence. Most participants who have chosen to disclose their study participation to others have had a lot of support. When deciding whether or not to participate in this study you are in the best position to assess your own personal situation about disclosure of study participation and or potential risk to yourself from others learning about your participation.

Additionally you will be given the option of using a study bag or a supermarket shopping bag for transporting study product to and from site.

BENEFITS
You may get no direct benefit from the screening tests. However, you may have a free physical exam including a genital examination. You will get counselling and testing for HIV and other infections.
We will give you treatment for any curable infections that are passed during sex, including syphilis. You can bring your partner here for free treatment for these infections if he needs it.
If you are infected with HIV, you will be referred for medical care, counselling, and other services available to you.
We will give you condoms and show you how to use them.
We will tell you about contraceptive methods and refer you to other family planning services if you wish.
You may speak with a study counsellor who may help with questions about the study and your health.

REASONS WHY YOU MAY BE WITHDRAWN FROM THE SCREENING TESTS WITHOUT YOUR CONSENT

You may be removed from the screening tests without your consent for the following reasons:

- The research study is stopped or cancelled.
- We establish that you are enrolled in another study of a vaginal or other experimental product for preventing HIV infection
- The study staff feels that having the screening tests would be harmful to you.
- You are not willing to find out your HIV test result.
- You are not able to attend clinic visits or complete the screening tests.
- Other administrative reasons.

Biomedical Research Ethics Committee
Nelson R Mandela School of Medicine
Approved Version
2 0 APR 2009

COSTS TO YOU

There is no cost to you for the screening tests.

COMPENSATION

You will be given a total of R150-00 for your transport and time for these screening visits, R50-00 for Screening Visit 1 and R100-00 for Screening Visit 2.

CONFIDENTIALITY

If, in the course of this study, we find you are encountering harm, require medical or non-medical care, or are posing a risk/harm to others, we may be obliged to share this information with relevant authorities. We will share information needed to verify your identity with other research organisations such as the MRC to ensure that you are not enrolled in other studies of vaginal gels or products. We may also ask you for a thumb print for this purpose. Otherwise all information you share with us will be kept confidential. You will be identified by a unique code. Personal information from your records will not be released without your written permission. You will not be personally identified in any publication about this study. However, your records may be reviewed by the SA MCC, the University of KwaZulu-Natal Biomedical Research Ethics Committee (BREC), FHI Protection of Human Subjects Committee (PHSC) or other regulatory bodies study monitors, study sponsors, and the companies that make the gel that is being tested in this research study. These agencies are government or sponsor appointed regulatory oversight or monitoring bodies responsible for ensuring that this trial is conducted in accordance with the study protocol and in compliance with international and local guidelines for the conduct of research involving human participants.

RESEARCH-RELATED INJURY

Based on what we know now, it is unlikely that you will be injured as a result of having the screening tests. If you are injured, the study staff will give you immediate necessary treatment for your injuries at the CAPRISA Clinical Research Site. You will not have to pay for this treatment. You will be told where you can get additional treatment for your injuries. There is no program for monetary compensation or other forms of compensation for such injuries. You do not give up any legal rights by signing this consent form.

PROBLEMS OR QUESTIONS

If you ever have any questions about the screening tests, or if you have a research-related injury, you should contact Professor Quarraisha Abdool Karim or Professor Salim S Abdool Karim at 031-260 4550, CAPRISA, Second Floor Doris Duke Medical Research Institute, Durban or Dr Janet Frohlich at 033-260 6851, CAPRISA Vulindlela Clinical Research Site, Mafakathini or Dr Koleka Mlisana at 031-260 1917 at the eThekwini Site.

If you have questions about your rights as a research participant, you should contact the Chairperson of the Biomedical Research Ethics Committee of the University of KwaZulu-Natal at 031-260 1074 or David Borasky at FHI at +091-919-544-7040 ext. 295.

Or you can write to the South African Medicines Control Council (MCC): The Registrar: SA Medicines Control Council, Department of Health, Private Bag X828, PRETORIA, 0001 Fax: (012) 323-4474, e-mail: labusa@health.gov.za

SIGNATURES

If you have read this consent form in the language of your choice, or had it read and explained to you, and you understand the information, and you voluntarily agree to have the study tests, please sign your name or make your mark below.

Participant Name (print)	Participant Signature	Date
Study Staff Conducting Consent Discussion (print)	Study Staff Signature	Date
Witness Name (Print)	Witness Signature	Date

The section below is to be completed by the person who administered the informed consent

Was a copy of the signed copy given to the volunteer: ☐ Yes ☐ No

If no, why not:

Biomedical Research Ethics Committee
Nelson R Mandela School of Medicine
Approved Version
20 APR 2009

Appendix IVb: Informed Consent Form for Enrolling Participants (separate document)

Biomedical Research Ethics Committee
Nelson R Mandela School of Medicine
Approved Version

2 0 APR 2009

CAPRISA 004
Phase IIb trial to assess the safety and effectiveness of the vaginal 1% tenofovir gel for the prevention of HIV infection in women in South Africa, Protocol Version 1.2, 05 January 2009

Version 1.10
27 March 2009

INFORMED CONSENT FORM FOR ENROLMENT

If the volunteer cannot read, this form must be read to the volunteer exactly as written, in the volunteer's language of choice, and a witness must sign this form to confirm that the correct information was given to the volunteer and that the volunteer freely consents to be in this study.

PRINCIPAL INVESTIGATORS:

Professor Salim S Abdool Karim
Professor Quarraisha Abdool Karim
2nd Floor Doris Duke Medical Research Institute
Nelson R Mandela School of Medicine
Private Bag 7, Congella 4013, Durban, South Africa
PHONE: 031-260 4550

Introduction

You are being asked to volunteer in the research study named above. The purpose of this research study is to find out if a vaginal gel, called tenofovir gel, can protect women from HIV. HIV is the virus that causes AIDS. This study is for women who are sexually active and could therefore get HIV or other sexually transmitted infections (STIs).

Your participation is voluntary

In order to be sure that you are informed about being in this research, we are asking you to read (or have read to you) this Consent Form in the language of your choice.

You will be asked to sign this Consent Form (or make your mark in front of a witness). We will give you a copy of this form to keep.

This Consent Form might contain some words that are unfamiliar to you. Please ask us to explain anything you may not understand.

Before you learn about the study, it is important that you know the following:

- Your participation is entirely voluntary.
- You may decide not to take part or to withdraw from the study at any time. You will not lose the benefits of your routine medical care at this site.
- If you decide to not take part in this research study, you can still take part in another research study, if one is available and you meet the study requirements.

PURPOSE OF THE STUDY

The purpose of the study is to find out if the gel formulation of an anti-retroviral drug called tenofovir can protect women at high risk from getting infected with HIV from their sex partner/s. Tenofovir gel is inserted into the vagina before and after sex.

The study of tenofovir gel is "experimental." This means we do not yet know all the effects of the Tenofovir gel, and we do not know if it works to protect against HIV. The Medicines Control Council (MCC) and the United States Food and Drug Administration (FDA) have not approved tenofovir gel for use in the general population for preventing HIV infection. However, the MCC has given permission for this study to be conducted to test if the tenofovir gel can protect against HIV and to establish how safe it is for long periods of time. There is no guarantee that the tenofovir gel works in preventing HIV infection in this study.

About 1250 women from Vulindlela and Durban will take part in this study.

Biomedical Research Ethics Committee
Nelson R Mandela School of Medicine
Approved Version
2 0 APR 2009

An earlier study with 84 women showed that tenofovir gel was safe to use in women who used Tenofovir gel once or twice per day. Tenofovir is detected in the blood at very low levels ~~within 15 minutes of the gel~~ application and is no longer detectable about 6 hours later. A recently completed study of 200 women from the USA and India reported that both daily and coitally dependent use of tenofovir gel was acceptable and safe.

To find out if Tenofovir gel works to prevent HIV, women who join this study will be put into two groups. One group of women will be given the gel that contains tenofovir. The other group of women will be given a different gel that does not contain tenofovir. This gel without tenofovir is called a "placebo." The placebo gel looks, smells, and feels like the gel with tenofovir but does not contain tenofovir. We know that the placebo gel does not protect women from getting HIV.

We do not know if the tenofovir gel will protect women against HIV. We think it might but we will not know until after studies like this one are completed.

All women in this study should use condoms each time they have sex, whether they are in the placebo group or the tenofovir group.

All women are put into one of these two groups by chance, like flipping a coin. This means that you will have an equal chance of being put in either group. You cannot choose your group and you will not be told which group you are in. Study staff will also not know which group you are in.

You will receive the same gel throughout the whole study.

PROCEDURES
If you agree to take part in the study, here is what you will do.

Your first visit will continue today, after you read, discuss, and sign this form. This visit will take about 3 hours. If you are menstruating, you may also need to come back 2 to 7 days after your menstrual period to complete your enrolment.

After enrolment, you will be in the study for a minimum of 6 months to a maximum of 30 months, depending on when you join. You will have a study visit every month while you are in the study. Visits will take about 60 to 90 minutes each. You will have one more visit about two months after you reach your final study visit. This is a safety visit two months following study product withdrawal to check for HIV infection and resistance.

All study visits will take place at the CAPRISA Vulindlela or eThekwini Clinical Research Sites.

During this study you may also be asked to participate in other sub-studies. You do not have to participate in any sub-studies if you do not want to. Separate consent will be sought for each sub-study that you wish to participate in.

While you are in this study, we ask that you do not:
- Insert any object or product into your vagina within two hours of putting in the gel.
- Take part in another research study on HIV prevention including vaginal gels or products.

Please remember that:
The products that we are testing have not been proven for safety beyond 3 months and we do not know if they can prevent HIV infection. Therefore:
  - It is important for your own safety that you do not participate in any other trial or study involving vaginal gels or other HIV prevention products
  - If a person uses gels or other study products from different studies at the same time and then has a problem that requires medical attention, we will not know whether the CAPRISA allocated gel or the other study product caused the problem.
  - If a participant is using study products from different studies at the same time, we cannot assess which study product is working.
  - The study staff will check if you are taking part in another HIV prevention study involving a gel or another experimental product by asking you questions.

Biomedical Research Ethics Committee
Nelson R Mandela School of Medicine
Approved Version
2 0 APR 2009

- o We will check what you tell us with other research organisations such as the South African Medical Research Council (MRC) to see if you are already participating in other research. If you join the CAPRISA 004 trial we will continue to check within CAPRISA and other research organizations such as the MRC to see if you have joined other research studies.
- o When we check with other studies and organizations such as the MRC only information needed to verify your identity and eligibility to take part in CAPRISA 004 will be shared. No other study related information such as data relating to your health status or behaviour will be disclosed.
- o If we find that you are in another study of an experimental product or any other HIV prevention interventions that involves an experimental product, the study staff will counsel you why it may not be safe for you to join more than one study at the same time.
- o If we find that you are in another study of an experimental vaginal product or any other HIV prevention interventions that involves an experimental product, the study staff will counsel you why it may not be safe for you to join more than one study at the same time and you will not be eligible to take part in the CAPRISA 004.
- o If we find that you participated in another study of an experimental vaginal product or any other HIV prevention interventions that involves an experimental product in the past 12 months, then you will not be eligible to take part in the CAPRISA 004.

You are asked to:

- Use your study assigned gel only in your vagina.
- Tell study staff if you join a research study including vaginal gels after you join this study.
- Tell study staff about any medications you take while you are in the study.
- Be on a reliable form of contraception that we will provide if required or refer you to an appropriate clinic or hospital

Enrolment Visit (Today)

During today's visit, we will provide you with any test results you have not received during your screening visits.

You will be given a unique study number that we call a participant identification number.

You will be assigned to a study gel group. You will be given tubes of your assigned gel that you can start using after you have been enrolled into this study. You will be shown how to use the gel. You will insert one applicator full of gel into your vagina up to 12 hours before each act of vaginal sex and again as soon as possible up to 12 hours after vaginal sex. You cannot use more than two gels in a 24 hour period even if you have sex more than once in a day.

We will take about 10 ml or about 2 teaspoons of blood to check for Hepatitis B infection. Hepatitis B is a virus that can cause liver problems. The blood will be sent to a laboratory for testing and you will be provided with your test results. If your laboratory results are abnormal you will be referred to Grey's or King Edward VIII Hospital for further care. If your hepatitis B test result is positive you will not be able to participate in this study. The reason for this is that the company that makes tenofovir recently expanded its use for treatment of Hepatitis B. In addition they have indicated in the package insert that if tenofovir is withdrawn it could worsen Hepatitis infection. Although this may not be true for the gel, in the interest of your safety we have decided to not enrol you if you have Hepatitis B infection.

An additional 10 teaspoons of blood will be taken from you at this visit for storage and may be used during, or at the end of the study, to recheck or conduct additional tests to help us understand how the gel works better. If you have not agreed to your specimens being stored for future testing not related to CAPRISA 004, these specimens will be destroyed after all the CAPRISA 004 tests have been completed.

During the study:

While you are enrolled in the study, you will have monthly study visits at this clinic. This is what will happen at those visits, including today's enrolment visit:

- You will answer questions about yourself, including your health, and your sexual practices.
- You will tell the study staff if you had any health problems since your last visit.

Biomedical Research Ethics Committee
Nelson R Mandela School of Medicine
Approved Version
2 0 APR 2009

- You will be examined by the study nurse or doctor for signs of illness. If needed, you will be given treatment for infections passed during sex, and be referred for medical care and other services.
- You will be counselled about other infections that may be passed during sex, and how to avoid these.
- We will ask you to update information on where you live and how to keep in contact with you.

Also, at each monthly study visit the following will happen:

- You will give study staff any gel that you did not use that month as well as all used applicators.
- You will tell study staff how many gels you used in the past month, how often, and when you have been using the gel including in relation to sexual activity.
- You will be given more gel and condoms for the upcoming month and counselled on how to use them.
- You will be asked about your contraceptive use and provided a method, if needed.
- You will be told about any test results from the previous month's visit if applicable.
- You will give urine at each visit for a pregnancy test. If you become pregnant, you will stop using the gel. Study staff will talk with you about your choices. If you become pregnant in between study visits, you should tell the study staff right away.
- You will be tested for HIV infection every month. This is described in detail below.

Once every 3 months:

- You will have an examination of your genital tract with a speculum to check if there are any effects of the gel.

At the following study visits: months 3, 12, your final study visit and, depending on when you were enrolled in the study, at your 24 month visit, about 20 ml or about 4 teaspoons of your blood will be taken to check if your blood, kidney and liver is fine. In addition about 11 teaspoons more blood will be collected at these visits and the following genital specimens will be collected during the scheduled pelvic examinations: vaginal aspirate, cervico-vaginal lavage (CVL), a swab and endocervical cytobrush. Vaginal aspirate specimens will be obtained by inserting an instrument similar to a syringe into the uppermost part of the vagina and collecting the secretions that accumulate naturally in the vagina from the cervix. CVL entails introducing about a tablespoon of water into your vagina and immediately removing it using a plastic syringe. The cytobrush specimens will be collected using a little brush to take a small sample of cells from your cervix (the neck of your vagina; which is at the upper end of your vagina). Swab specimens will be collected inserting a swab that looks like an ear bud. This swab will be rolled over the surface of the vaginal wall and endocervix to collect secretions. These procedures will be conducted by trained clinical staff at the time of pelvic examination and will take about 5 minutes to complete. Every measure will be taken to minimise discomfort these procedures may cause. These specimens will be stored and may be used during, or at the end of the study, to recheck or conduct additional tests to help us understand how the gel works better. If you have not agreed to your specimens being stored for future testing not related to CAPRISA 004, these specimens will be destroyed after all the CAPRISA 004 tests have been completed.

HIV Testing

You will be tested for HIV infection every month. You will first talk to the study staff about the HIV test and through a finger-prick a small amount of blood will be drawn from your finger for HIV testing.
You will know your HIV test results on the same day you have the test. If you are HIV negative on these test results you will continue in the study. Sometimes HIV tests are not clearly positive but also not negative and if this HIV test at the site shows that you may have become infected at a scheduled or unscheduled study visit, we will draw about 4 teaspoons of blood from your arm with a needle into a blood collection tubes to undertake different HIV tests at the CAPRISA laboratories in Durban until we know the result for sure. If tests conducted using this blood draw show that you may have HIV infection, we will need to repeat the test and will need to collect another blood specimen from your arm to do a different test to confirm this result. These additional HIV tests take about 1-2 weeks so you will be given an appointment to return to this clinic to get these results. If this second test shows that you have HIV another blood specimen will be taken to repeat the test once more before we finally confirm if you have HIV.

It is possible, both naturally and in the presence of tenofovir, to have instances where the existing HIV test results may be indeterminate, negative or undetectable and yet infection could be present. We will need to use some additional HIV tests to enable us to detect HIV infection in these instances. It is important to be able to identify participants who are HIV infected as rapidly as possible to minimise exposure to the product

Biomedical Research Ethics Committee
Nelson R Mandela School of Medicine
Approved Version
20 APR 2009

as rapidly as possible and thereby minimise emergence of resistance. To undertake these tests we will need to take an additional 1.2 teaspoons or about 60 ml of blood and genital specimens (as described above) from you if we think you are infected with HIV. The collection of genital specimens in potential seroconvertors is for drug levels and resistance testing of the virus in the genital compartment. This will assist us in determining whether resistance is present in the transmitted virus or whether resistance was acquired.

If your HIV test results from the additional tests do not help us determine your HIV status we will continue undertaking HIV tests until we can confirm your result for sure as either HIV infected or not.

You will continue using your assigned study gel in the meantime while the different tests are being done. If the final HIV test result is positive, you will be stopped from using gel. If the HIV tests show that you are not infected with HIV, you will continue with gel use.

You must receive your HIV test results to stay in the study. You will talk with study staff about the meaning of your results and how you feel about them.

If you become infected with HIV during the study

It is possible that you could become infected with HIV during the study. If this happens we will refer you to the CAPRISA Acute Infection Study (CAPRISA 002). This is a study for people who have just become infected. If you choose to join CAPRISA 002 you will be provided additional information about this study and will need to provide informed consent to join this study. You will be in follow-up in CAPRISA 002 if you choose to join until you require to go onto antiretroviral treatment. At this point you will be referred to the CAPRISA AIDS Treatment (CAT) Programme where you will be able to access antiretroviral treatment. If you choose not to join CAPRISA 002 you can be referred to the CAT Programme where you will receive care and be initiated on ARV treatment when eligible. If you choose not to join CAPRISA 002 or CAT we will refer to other sources of care, treatment, or support..

If you become infected with HIV during the study and this has been confirmed with the procedures described above and you have been put on product hold, you will need to come back to the clinic for a final study visit about 3 months later. At this final study visit about 12 teaspoons of blood be taken from you at the time of infection for HIV testing as described above.

If you become infected with HIV during the study you will be required to stay in the study for three months but you will no longer be able to use the gel because we are not sure about the effects of the gel in HIV infected women.

At any time during the study:

You are asked to tell the study staff about medical problems you have during the study, especially genital problems. You can also contact the study staff between regular visits to report these problems. The study staff will examine you as needed. They will either provide or refer you for medical care that you may need.

If you are having health problems that may be caused by infections passed during sex, you will be given an examination of your genital area and inside your vagina. You will get treatment for curable infections passed during sex if you need it. If you have any infections that your partner may also have, you can bring him here for treatment that he may need too. This will be at no cost to you or your partner.

You can have extra counselling and testing for HIV if needed between regular visits. If you wish, your partner can have HIV counselling with you.

Last study visit:

At your last study visit you will do all the things described above plus the following.

You will have two teaspoons of blood taken for Hepatitis B testing. You will be asked to return to this site after two months for your results. If there results are abnormal you will be referred to Grey's or King Edward VIII Hospitals for further care.

You will have an examination of your vagina and a study doctor or nurse will collect specimens from your vagina and genital tract for storage.

Biomedical Research Ethics Committee
Nelson R Mandela School of Medicine
Approved Version

2 0 APR 2009

You will be asked questions about what you thought of the gel.

Additional visit two months after study exit and product hold
All participants will be asked to return to the study site two months after this study exit visit and after stopping study product use for additional safety tests to be undertaken. As this is the first tenofovir gel trial of extended use we do not know what will happen after you stop using the study gel. By undertaking an HIV test at this point and if your HIV tests indicate that you may be infected with HIV about 12 teaspoons of blood will be taken for tenofovir resistance testing (see section on risks below for more information on resistance) it will help us understand this better and ensure your continued safety. If you had Hepatitis B and were enrolled into the trial we will also take about 2 teaspoons of blood to check how well your liver is working. You will receive your HIV test results at this visit. If you are infected or potentially infected at this visit you will be referred to the CAPRISA 002 study to confirm your HIV test results and for ongoing follow-up and care if indicated.

Contact Procedures:
Once you join the study, it is very important for us to stay in touch with you and find out how you are doing. We will ask for your name, address, phone number, and other contact information at your first study visit. We also will ask for the names and contact information of people we can contact if we cannot reach you.

We will ask you to update this information at each study visit. We will use your contact information to remind you of scheduled study visits. If you miss a visit, we may call or send letters or visit your home to find you. We also will try to reach you through the contact people that you list for us. If we talk to these people, we will not tell them why we are trying to reach you.

RISKS AND/OR DISCOMFORTS
Some women who have used these gels in other studies had some vaginal irritation and burning, mild vaginal bleeding, genital sores, pain when urinating, abdominal pain, nausea and diarrhoea. You also may have these. You may also have other symptoms we do not know about yet.

It is possible that genital sores may increase the chance of getting HIV and other STIs. If you have any pain or discomforts after using the gel, tell the study nurse or doctor.

There is also a chance that the gels could be absorbed from the genital tract into the blood. It is not known whether this causes any harm.

There is also a possibility that if you become infected with HIV and continue using the gel that it could cause the virus to resist being treated by some ARVs. This is called “drug resistance”. Resistance is a complex phenomenon and it can occur naturally as the virus replicates itself or in the presence of an ARV drug but can only occur in someone who is already infected with HIV. We do not know if drug resistance will be an issue for women using tenofovir and will monitor this in women who become infected in CAPRISA 004. We also do not know whether women who become infected after exposure to Tenofovir Gel will respond differently to anti-retroviral treatment.

You may be allergic to the condom material. “Allergic” means that you have itching, swelling or skin irritation where the condom touches your skin. If this happens, tell the study nurse or doctor.

You may feel discomfort or pain during the exam of your genital tract.
You may feel discomfort when your blood is drawn. You may feel dizzy or faint. You may have a bruise, swelling or infection where the needle goes into your arm.

You may become embarrassed, worried, or anxious when discussing talking about your sexual practices, ways to protect against HIV and other infections passed during sex, and your test results.

You may become worried or anxious while waiting for your test results.

If you have HIV or other infections, you may become worried or anxious. A trained counselor will help you deal with any feelings or questions you have.

Biomedical Research Ethics Committee
Nelson R Mandela School of Medicine
Approved Version

2 0 APR 2009

However, it is possible that your partner, other family members or others may learn of your participation in this study for example through observing you visit the CAPRISA facility, or carrying a study bag or seeing where you store study product or through other means. These individual(s) may think you have HIV, or are at "high risk" for getting infected with HIV or they may get upset or angry with you for other reasons such as you are eligible for study participation and they are not, or you are receiving money for study participation, or you have access to gel and they do not or they have less control over you, or you have better access to information and better quality health services than they have. This may result in these people and possibly others treating you unfairly or discriminating against you for example making it difficult for you to get or keep a job, or being accepted by your family or community members or threaten you with violence. If you ever experience any of this discrimination or violence please you should feel free to contact the study staff telephonically or come to the clinic. You do not need to wait for your scheduled study visit to report this to study staff or seek assistance from staff. All of our staff have been trained to provide you with appropriate counselling and support. In addition we have a list of resources that can provide you with additional support or resources that are available to you to adequately assist you in dealing with these experiences.

While we respect your right to disclose your study participation with others it is hard to predict whether they will be supportive or not or threaten you with violence. Most participants who have chosen to disclose their study participation to others have had a lot of support. When deciding whether or not to participate in this study you are in the best position to assess your own personal situation about disclosure of study participation and or potential risk to yourself from others learning about your participation.

Additionally you will be given the option of using a study bag or a supermarket shopping bag for transporting study product to and from site.

There are other possible risks related to using the gel that we do not know about. One purpose of this study is to understand what the other risks might be.

To reduce risk and discomforts, we will make sure that women are healthy before they join the study. We will also check their health throughout the study. To help reduce risk, you will be asked to:

- come for all scheduled study visits
- tell study staff about any concerns you have with the study gel or if you have any side effects
- use the gel only as prescribed
- return all your used and unused gel to the clinic each month

PREGNANCY

It is not known if the gels have any effect on pregnancy in women, or whether the gel has any effect on the foetus. Because of this, pregnant women may not join this study.

You must have a negative pregnancy test before you join this study. At the time of joining the study you must be on a non-barrier method of contraception such as birth control pills or Depo-Provera or Nuristerate. We can provide some types of contraception through the study pharmacy. The study staff will counsel you and provide you with contraception if needed, or refer you to another clinic where you can get contraception.

If you become pregnant during the study, tell the study nurse or doctor right away. You will stop using the gel but will continue with your scheduled study follow-up visits. The study nurse or doctor will talk with you about your choices.

BENEFITS

You may get no direct benefit from being in this study. But you may get some personal satisfaction from being part of a research study on HIV prevention. You or others may benefit in the future from information learned in this study.

You may have a free physical exam including a genital exam.

You will be provided with free contraception.

You will get counselling and testing for HIV and other infections.

Biomedical Research Ethics Committee
Nelson R Mandela School of Medicine
Approved Version
2 0 APR 2009

We will give you treatment for any curable infections that are passed during sex, including syphilis. You can bring your partner here for free treatment for these infections if he needs it.
If you are infected with HIV, you will be referred for medical care, counselling, and other services and research studies available to you. Your participation in this research is voluntary. Some of these studies will help us monitor tenofovir resistance as well as the implications of tenofovir gel exposure on anti-retroviral treatment outcomes in women who become infected in CAPRISA 004.

If you become pregnant, you will be referred for medical care and other services that you and your baby may need.

We will give you condoms and show you how to use them.

You may speak with a study counsellor who may help with questions about the study and your health.

NEW FINDINGS
You will be told any new information learned during the course of the study that might cause you to change your mind about staying in the study. At the end of the study, you will be told when the study results may be available and how to learn about them.

REASONS WHY YOU MAY BE WITHDRAWN FROM THE STUDY WITHOUT YOUR CONSENT
You may be removed from the study without your consent for the following reasons:
- The investigator decides that continuing in the study would be harmful to you.
- You are unable to use the assigned gel as instructed, or to keep study appointments.
- You have a bad effect from the gel.
- You become pregnant.
- You need a treatment not allowed on this study.
- The study is cancelled by the SA MCC, University of KwaZulu-Natal Biomedical Research Ethics Committee (BREC) or Family Health International's Protection of Human Subjects Committee (PHSC), or the company that makes the gels.
- If after enrolling in CAPRISA 004 we find that you have joined another study, the CAPRISA 004 study team will make a decision on whether this poses any harm to you and its implications for CAPRISA 004. They will inform you of the decision that will be in your best interest in terms of minimizing harm as well as for CAPRISA 004. They will counsel you as to why it may not be safe for you to join more than one study at the same time.
- If you joined CAPRISA 004 after being enrolled in another study of an HIV prevention product you will be terminated from CAPRISA 004 as you will not be eligible for inclusion in CAPRISA 004.
- If you join another study after enrolling in CAPRISA 004 the study team of the study you joined after joining CAPRISA 004 will be informed of your participation in CAPRISA 004. Each team will make an independent decision about your continued participation in either study based on assessment of potential harm to yourself and the implications for each study.
- Other administrative reasons.

ALTERNATIVES TO PARTICIPATION
There are no vaginal gels known to prevent HIV infection. The only known way to prevent HIV infection during sex is to use a condom every time you have sex. The benefits of medical male circumcision for women are not known. However, there may be other research studies that are testing ways to prevent HIV infection in this area that you may be eligible to participate in. However, if you choose to participate in an alternative study on HIV prevention that involves an experimental study product you will not be eligible for participation in CAPRISA 004.

COSTS TO YOU
There is no cost to you for taking part in this study.

COMPENSATION
You will be compensated R150-00 for your transport and time for each scheduled visit. For staff initiated interim visits you will be compensated R50-00.

CONFIDENTIALITY

If, in the course of this study, we find you are encountering harm, require medical or non-medical care, or are posing a risk/harm to others, we may be obliged to share this information with relevant authorities. We will share information needed to verify your identity with other research organizations to ensure that you are not enrolled in other studies of vaginal gels or products. We may also ask you for a thumb print for this purpose. Otherwise all information you share with us will be kept confidential. You will be identified by a unique code. Personal information from your records will not be released without your written permission. You will not be personally identified in any publication about this study. However, your records may be reviewed by the SA MCC, the BREC, the PHSC or other regulatory bodies, study monitors, study sponsors, and the companies that make the gel that is being testing in this research study. These agencies are government or sponsor appointed regulatory oversight or monitoring bodies responsible for ensuring that this trial is conducted in accordance with the study protocol and in compliance with international and local guidelines for the conduct of research involving human participants.

RESEARCH-RELATED INJURY

Based on what we know now, it is unlikely that you will be injured as a result of being in this study. If you are injured as a result of being in this study, you will be given the immediate necessary treatment for your injuries at the CAPRISA Clinical Research Site. You will not have to pay for this treatment. You will be told where you may receive additional treatment for your injuries. There is no program for monetary compensation or other forms of compensation for such injuries. You do not give up any legal rights by signing this consent form.

PROBLEMS OR QUESTIONS

If you ever have any questions about the your participation in this study you should contact Professor Quarraisha Abdool Karim or Professor Salim S Abdool Karim at 031-260 4550, CAPRISA, Second Floor Doris Duke Medical Research Institute, Durban or Dr Janet Frohlich at 033-260 6851, CAPRISA Vulindlela Clinical Research Site, Mafakathini or Dr Koleka Mlisana at 031-260 1917 at the eThekwini Site.

If you have questions about your rights as a research participant, you should contact the Chairperson of the Biomedical Research Ethics Committee of the University of KwaZulu-Natal at 031-260 1074 in Durban or David Borasky at FHI +091-919-544-7040 ext. 295.

Or you can write to the South African Medicines Control Council (MCC): The Registrar: SA Medicines Control Council, Department of Health, Private Bag X828, PRETORIA, 0001 Fax: (012) 323-4474, e-mail: labusa@health.gov.za

Biomedical Research Ethics Committee
Nelson R Mandela School of Medicine
Approved Version
2 0 APR 2009

SIGNATURE PAGE:
If you have read the informed consent (or if you have had it read to you) and understand the information, and you voluntarily agree to join this study, please sign your name below. In addition you will asked to sign a declaration stating that you are not enrolled in any study involving an investigational product.

Volunteer's name (print as in ID book or birth certificate)	Volunteer's signature	Date
Name of staff member who administered consent (print)	Staff member's signature	Date
Witness' name (print)	Witness' signature	Date

The section below is to be completed by the person who administered the informed consent

Was a copy of the signed copy given to the volunteer: ☐ Yes ☐ No
If no, why not:

Was the enrolment comprehension assessment completed: ☐ Yes ☐ No

If no, why not:

Biomedical Research Ethics Committee
Nelson R Mandela School of Medicine
Approved Version
20 APR 2009

I .... (Full name as in ID book) confirm that I am not currently enrolled in any HIV prevention trial including vaginal gels or other products. I understand that for my own safety I should tell the CAPRISA 004 staff if I intend to join another study on vaginal gels or products. I understand that enrolling in more than one study on a vaginal gel or other study product at the same time might affect my health in a way that it may be difficult to tell if the reaction I am having is as a result of participating in CAPRISA 004 or of participating in another study. I understand that study staff will continue to check with other research organisations such as the South African Medical Research Council (MRC) if I am participating in other HIV prevention research studies.

______________________   __/__/____

Participant's Signature   Date

______________________   ______________   __/__/____

Staff Name   Staff Signature   Date

Biomedical Research Ethics Committee
Nelson R Mandela School of Medicine
Approved Version
20 APR 2009

Appendix IVc: Informed Consent Form for Specimen Storage and Possible Future Research Testing (Separate document)

CAPRISA 004

Phase IIb trial to assess the safety and effectiveness of the vaginal 1% tenofovir gel for the prevention of HIV infection in women in South Africa Protocol Version 1.1, 20 April 2007

Version 1. 4
29 July 2007

INFORMED CONSENT FOR SPECIMEN STORAGE FOR POSSIBLE FUTURE RESEARCH

If the volunteer cannot read, this form must be read to the volunteer exactly as written, in the volunteer's language of choice, and a witness must sign this form to confirm that the correct information was given to the volunteer and that the volunteer freely consents to be in this study.

Biomedical Research Ethics Committee
Nelson R Mandela School of Medicine
Approved Version

0 6 SEP 2007

PRINCIPAL INVESTIGATORS:
Professor Salim S Abdool Karim
Professor Quarraisha Abdool Karim
2nd Floor Doris Duke Medical Research Institute
Nelson R Mandela School of Medicine
Private Bag 7, Congella 4013
Durban, South Africa
PHONE: 031-260 4550

SPECIMEN STORAGE

Introduction

You have decided to take part in the research study listed above. While you are in this study, there may be some remaining blood and genital specimens taken from you for the purposes of this study that might be useful for future research. You are being asked to agree to the storage of your blood and vaginal specimens for possible future research. This is research that will be conducted in the future that may or may not be related to the tenofovir gel study.

This consent form gives you information about the collection, storage, and use of your blood and genital specimens for possible future research. The study staff will talk to you about this information. Please ask if you have any questions. If you agree to the storage of your blood and genital specimens for possible future research, you will be asked to sign this consent form. You will get a copy of this form to keep.

You can still take part in the tenofovir gel research study even if you decide not to sign this form. If you decide not to sign this form the specimens described below will be collected from you and after all the tenofovir gel study related testing has been completed all remaining specimens will be destroyed.

HOW WILL YOU GET THE BLOOD AND VAGINAL SPECIMENS FROM ME?

The study staff will take blood from you when you come for the following study visits: at enrolment, , 3 month, 12 month, 24 month and your last study visits. At each of these visits we will take about sixty ml or about 1 teaspoons of blood with a needle from your arm for the study. This blood is needed to carry out the regular tests for the research study. If you agree to have your specimens stored for possible future research, we will store the remainder of this blood after the tests for this study has been completed for possible future research that is not part of the tenofovir gel research study.

In addition, as part of the tenofovir gel research study, you will have a pelvic examination when you come for the following study visits: at the 3 month, 12 month, 24 month, and your last study visits. The following genital specimens will be collected during these scheduled pelvic examinations: vaginal aspirate, cervico-vaginal lavage (CVL), a swab and endocervical cytobrush. These specimens will be stored and may be used during, or at the end of the study, to recheck or conduct additional tests to help us understand how the gel works better. If you have not agreed to your specimens being stored for future testing not related to CAPRISA 004, these specimens will be destroyed after all the CAPRISA 004 tests have been completed.

If you agree to have your specimens stored for possible future research, these genital specimens will be kept and used for possible future research that is not related to the tenofovir gel research study.

HOW WILL YOU USE MY STORED BLOOD AND VAGINAL SPECIMENS?
Researchers at CAPRISA and elsewhere will use your blood and vaginal specimens to look for HIV and other infections, or for damage caused by such infections, or your body's response to infection.

Researchers may also look at your genes (DNA), since genes can affect the way your body responds to infections in important ways. Your genes might make you more or less likely to get infected, or make your responses to infection or to treatment stronger or weaker. If you become infected with HIV your genes might also affect how fast or slow you develop AIDS.

We do not plan to contact you or your regular doctor with any results from tests done on your stored blood or vaginal specimens. This is because research tests are often experimental and we don't think the results will be useful for making decisions about your health. Additionally these tests will be done in a way that will make it extremely difficult to link the test results to you. Your blood will not be sold or used directly to produce commercial products. You will not be compensated for use of your stored specimens.

All future research studies using your samples will be reviewed first by the CAPRISA Scientific Review Committee and a special committee at the Nelson R Mandela School of Medicine Biomedical Research Ethics Committee.

HOW LONG WILL YOU KEEP MY BLOOD AND GENITAL SPECIMENS?
There is no time limit on how long your blood and vaginal specimens will be stored.

HOW WILL MY BLOOD AND GENITAL SPECIMENS BE STORED?
Your blood and genital specimens will be stored at special facilities that are designed to store blood samples safely and securely. The storage facilities are based at the CAPRISA research Laboratory, Doris Duke Medical Research Institute, Nelson R Mandela School of Medicine. The storage facilities are designed so that only approved researchers can have access to the blood and genital specimens. Some employees of the storage facilities will need to have access to your blood specimens in order to store them and to keep track of where they are, but these people will not have information that directly identifies you.

DOES STORAGE OF MY BLOOD AND VAGINAL SPECIMENS BENEFIT ME?
It is unlikely that there will be any direct benefits to you from tests done on your stored specimens. There may be benefits to society of doing research on stored blood and genital specimens. These benefits may include learning more about HIV infection.

WHAT ARE THE RISKS?
There are few risks related to storing your blood and genital specimens. When future tests are done on the stored blood and vaginal specimens, there is a small but possible risk to your privacy.

WHAT ABOUT CONFIDENTIALITY?
In order to keep your information private, your blood and genital specimens will be labelled with a code. Your personal information (name, address, phone number) will not be placed on the specimens. Only the research clinic where you come for study visits will be able to link the code with your personal information.

In the future, when researchers are given your stored specimens to study, they will be given only the code; they will not be given your personal information.

The results of tests done on your stored specimens will not be included in your health records. Every effort will be made to keep your personal information confidential, but we cannot guarantee absolute confidentiality. Your personal information may be disclosed if required by law.

WHAT ARE MY RIGHTS?
Allowing your blood and genital specimens to be stored for future research is completely voluntary. You may decide not to have any blood or genital specimens stored for future research. You can still be in this research study or any future study if you decide not to allow your specimens to be stored for future research. However, specimens will still be collected from you and stored in order to complete what is needed for the tenofovir gel research study.

Even, if you decide now that your blood and genital specimens can be stored for possible future research, you may change your mind at any time. If you change your mind, you must contact your study doctor or nurse and let them know that you do not want your blood and genital specimens used for future

Biomedical Research Ethics Council
Nelson R Mandela School of Medic
Approved Version
06 SEP 2007

research. Then no additional specimens will be collected from you for storage. Your blood and genital specimens in storage will be destroyed after all study related testing has been completed, included quality assurance testing.

WHAT DO I DO IF I HAVE QUESTIONS?

If you ever have any questions about the storage of your blood or genital specimens you should contact Professor Quarraisha Abdool Karim or Professor Salim S Abdool Karim at 031-260 4550, CAPRISA, Second Floor Doris Duke Medical Research Institute, Durban or Dr Janet Frohlich at 033-260 6851, CAPRISA Vulindlela Clinical Research Site, Mafakathini or Dr Ayesha Kharsany at 031- 260 1917 at the eThekwini Site.

If you have questions about your rights as a research participant, you should contact the Chairperson of the Bio-medical Research Ethics Committee of Nelson R Mandela School of Medicine, Faculty of Health Sciences, University of KwaZulu-Natal at 031-260 4604/4495 or David Borasky at FHI +091-919-544-7040 ext. 295.

Or you can write to the South African Medicines Control Council (MCC): The Registrar: SA Medicines Control Council, Department of Health, Private Bag X828, PRETORIA, 0001 Fax: (012) 323-4474, e-mail: labusa@health.gov.za

SIGNATURES

Please carefully read the statements below and think about your choice. No matter what you decide it will not affect your care or your participation in the tenofovir gel research study.

I agree to have blood and genital specimens taken for the purpose of storage and testing for future research related to HIV and other infections.

_____ Yes

_____ No

______________________	______________________	____________
Participant Name (Print)	Participant Signature	Date
Study Staff Conducting Consent Discussion (print)	Staff Signature	Date
Witness Name (print) (If participant is unable to provide a signature)	Witness Signature	Date

The section below is to be completed by the person who administered the informed consent

Was a copy of the signed copy given to the volunteer: ☐ Yes ☐ No

If no, why not:

____

____

Biomedical Research Ethics Committee
Nelson R Mandela School of Medicine
Approved Version
0 6 SEP 2007
